# Using Risk Assessment and Habitat Suitability Models to Prioritise Invasive Species for Management in a Changing Climate

**DOI:** 10.1371/journal.pone.0165292

**Published:** 2016-10-21

**Authors:** Shauna-Lee Chai, Jian Zhang, Amy Nixon, Scott Nielsen

**Affiliations:** 1 Ecosystem Management, Alberta Innovates-Technology Futures, Vegreville, Alberta, Canada; 2 Department of Renewable Resources, University of Alberta, Edmonton, Alberta, Canada; 3 Alberta Biodiversity Monitoring Institute, University of Alberta, Edmonton, Alberta, Canada; Fudan University, CHINA

## Abstract

Accounting for climate change in invasive species risk assessments improves our understanding of potential future impacts and enhances our preparedness for the arrival of new non-native species. We combined traditional risk assessment for invasive species with habitat suitability modeling to assess risk to biodiversity based on climate change. We demonstrate our method by assessing the risk for 15 potentially new invasive plant species to Alberta, Canada, an area where climate change is expected to facilitate the poleward expansion of invasive species ranges. Of the 15 species assessed, the three terrestrial invasive plant species that could pose the greatest threat to Alberta’s biodiversity are giant knotweed (*Fallopia sachalinensis*), tamarisk (*Tamarix chinensis*), and alkali swainsonpea (*Sphaerophysa salsula*). We characterise giant knotweed as ‘extremely invasive’, with 21 times the suitable habitat between baseline and future projected climate. Tamarisk is ‘extremely invasive’ with a 64% increase in suitable habitat, and alkali swainsonpea is ‘highly invasive’ with a 21% increase in suitable habitat. Our methodology can be used to predict and prioritise potentially new invasive species for their impact on biodiversity in the context of climate change.

## Introduction

Climate change is likely to favour invasive species through increased disturbance events (such as fire, flood, storms and drought), more hospitable climates for invasive species to become established and decreased resistance of native communities to invasion [1–5]. Traits of invasive plant species are also more likely to benefit from climate change than traits of native species [6,7]. These traits include short generation time, good dispersal ability, broad environmental tolerance and rapid growth [7]. Thus, some native plant communities will likely receive numerous non-native species due to climate change, and while some non-natives will exert relatively benign impacts on native communities, others will pose a significant threat to their survival [8–10].

Managers of biological invasions require pre-emptive information on invasive species distributions so that risks can be assessed, and suitable strategies can be formulated in a timely manner [11]. In a changing climate, risk assessments need to consider the availability of suitable habitat [9]. Numerous risk assessment tools have been developed to quantify risk posed by invasive species (for example, [12–14]). However, none of these risk assessments consider by themselves the impact of climate change on the suitable habitat available to a species [9]. Recently, the development of habitat suitability models of invasive species ranges has proven valuable to informing policies and decision-making, and identifying new potential areas of invasion [3,15,16]. By incorporating habitat suitability models with risk assessment, managers can assess the risk posed by invasive species acting under climate change, especially for new invasive species that could potentially arrive. With increased realisation that climate change influences risk assessments, recent attempts have been made to incorporate invasive species demographic rates, dispersal and other spatially explicit criteria with habitat suitability models to assess risk by *existing* invasive species [17–19]. No study has however incorporated the full risk assessment process (which includes assessing species traits, ecological impacts, distribution and feasibility of control) for *new* introductions with habitat suitability modeling considering climate change. We describe a method for invasive species prioritisation that combines habitat suitability modeling under climate change with risk assessment to biodiversity that can be applied to different jurisdictions. We demonstrate the method for a high latitude region of North America, the province of Alberta, Canada where climate change may facilitate the northward expansion of invasive species ranges from further south [20–22]. Our objective is to predict and prioritise invasive species that threaten biodiversity, and are not currently present in Alberta, but could expand their range due to climate change.

## Methods

### Study species and region

The study area is the high latitude Canadian province of Alberta, located 49–60°N and 110–120°W, with an area of 66.2 million hectares. The climate is characterised by a short summer and long, cold winter, with mean coldest monthly temperature (in January) ranging from -25.1°C to -11.7°C [22]. Historically, Alberta’s climate has been relatively inhospitable to a number of invasive species that inhabit neighbouring jurisdictions. In anticipation of climate warming and associated poleward expansion of invasive species ranges [20,21], we chose to examine species present in states south of Alberta’s border (Montana, North Dakota, South Dakota, Wyoming, Idaho, Washington, Oregon), and in provinces to the east (Manitoba, Saskatchewan, Ontario) and west of Alberta’s border (British Columbia). We did not examine species already present in Alberta, since pre-empting and eradicating new invasions is the most efficient approach to managing invasive species [23].

**Fig 1:**
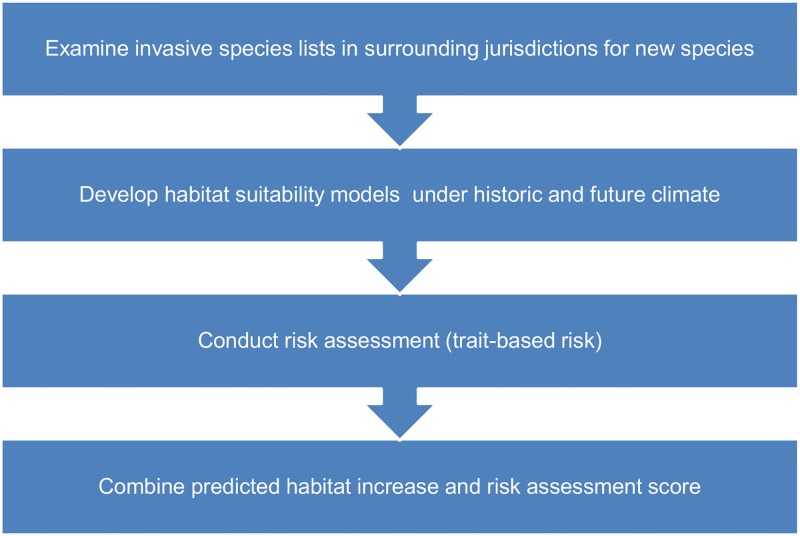
Summary of methods used to predict and prioritise invasive species in a changing climate.

We selected 15 non-native plant species (not yet present in Alberta) by examining regulated lists in nearby jurisdictions, and assessed the potential risk to Alberta based on their traits and projected change in suitable habitat between baseline and future climates in Alberta (Fig 1 and Table 1).

**Table 1:** Study species and species location data used in habitat suitability modeling. The data sources were the Global Biodiversity Information Facility (GBIF) and Atlas Florae Europaeae (AFE).

Common name	Scientific name	Number of occurrence records	Data source
African rue	*Peganum harmala*	827	GBIF
alkali swainsonpea	*Sphaerophysa salsula*	100	GBIF
autumn olive	*Elaeagnus umbellata*	897	GBIF
black swallow-wort	*Vincetoxicum nigrum*	977	GBIF
gorse	*Ulex europaeus*	62,305	GBIF
knapweed, brown	*Centaurea jacea* (sensu lato)	62,232	GBIF
knotweed, giant	*Fallopia sachalinensis*	4,348	GBIF
medusahead	*Taeniatherum caput-medusae*	1,832	GBIF
puncturevine	*Tribulus terrestris*	4,065	GBIF
saltlover	*Halogeton glomeratus*	208	GBIF, AFE
Scotch broom	*Cytisus scoparius*	77,275	GBIF
Scotch thistle	*Onopordum acanthium*	10,701	GBIF
Syrian bean-caper	*Zygophyllum fabago*	262	GBIF
tamarisk, Chinese	*Tamarix chinensis*	326	GBIF
thistle, globe	*Echinops sphaerocephalus*	3,084	GBIF

### Habitat suitability modeling

Species distribution modeling is commonly used for projecting the suitable habitat of invasive plants with climate change (e.g., [24]). These models use a correlative approach between observed species locations and climate/environmental variables to predict habitat suitability outside of the observations. The assumptions are that species distribution is in equilibrium with the current climate, and that climate is a strong determinant of species distribution [25]. The limitations of this approach, especially for invasive species, is that today’s distribution of species locations may not be in equilibrium with current climate, that is, they may not be distributed over their full potential climate niche due to factors such as dispersal limitation, competition, predation, and human management [26]. It may take centuries or millennia for invasive species to stabilise [10]. Notwithstanding this caveat, habitat suitability models have been shown to be highly predictive in determining climate niches for a variety of species [26–28].

We used habitat suitability models to assess the climate change-related risk in Alberta of each study species. We developed spatial projections of potentially suitable habitat for each species in Alberta under baseline (1961–1990) and future climates at a 4 km resolution. Future climate in Alberta was modeled using climate projections for the time frame 2041–2070 (2050s), because we expect to see pronounced effects of climate change by this timeframe [29], and it is still a reasonable timeframe over which management planning can occur.

#### Species data

We obtained species location data from the Global Biodiversity Information Facility (GBIF), limiting our search to records with geographic coordinates [30]. Location data for one species (*Halogeton glomeratus*) was supplemented with records requested from the Atlas Florae Europaeae. These data were obtained through personal communication with Alexander Sennikov, Secretary of the Committee for Mapping the Flora of Europe on August 30, 2013. The final data set contained between 100 and 87,687 records for each species (Table 1). Location data from both native and invaded ranges were used to model habitat suitability because the combined data set is more relevant for potential invasions into Alberta in the context of climate change, than location data from the native range alone [15], [31]. Using both native and introduced ranges also compensated for uneven coverage of data across parts of Eastern Europe and Asia, as this decreased the chance of having unrepresented climates in the model.

#### Environment data

We used a combination of climate (baseline or future) and soil variables to model habitat suitability for the 15 study species in Alberta. Baseline climate (1961–1990) was comprised of 19 bioclimatic variables at 2.5 arc minute (nearly 4.6 km) resolution from Worldclim [32]. Future climate (2041–2070) was based on CliMond 10′ gridded climate data from CSIRO Mk3.0, A2 scenario [20] which is similar to RCP 8.5 and describes a high emission scenario [33]. We also used a global data set of derived soil properties (0.5-degree grid, [34]).

To reduce multi-collinearity, Pearson’s correlation and variance inflation factor (VIF) were used for variable selection [35]. A pairwise Pearson correlation coefficient (absolute value) of 0.7 was used as a threshold of correlation, and a VIF > 10 was used as an indication of collinearity.

#### Species distribution modeling

We used maximum entropy to predict habitat suitability for each of the 15 study species in both current and future Alberta climates [36,37]. This approach is appropriate for habitat suitability based on “presence-only” data, such as data from herbaria where species absences are not explicitly recorded [38]. The analyses were conducted using MaxEnt (Version 3.3.3k; [36]) and R package ‘dismo’ [39]. To model each species’ distribution using presence-only location data, each set of observations was divided into a training dataset (75% of data), used to develop the model, and a testing dataset (25% of data), used to evaluate the performance of the model. Model performance was evaluated using the area under the curve (AUC) of a receiver operating characteristic (ROC; [40]). An AUC value of 0.5 implies random predictive discrimination, while values above 0.7, 0.8 and 0.9 represent good, very good and excellent discrimination, respectively [40,41].

We used the models to predict distributions of suitable habitat for the 15 species under both current and future climates for Alberta. Habitat suitability was initially predicted as a continuous variable, then converted into categories, representing low risk (*suitable low risk habitat*) and high risk (*suitable high risk habitat*) habitat using model specific probability thresholds [42]. The low threshold, identifying low risk habitat, was chosen using the least training presence threshold [43]; the higher threshold, identifying habitat most likely to be at risk of invasion (i.e., suitable high risk habitat), was defined by sensitivity-specificity sum maximization (e.g., [44]). We used the *suitable high risk habitat* to help determine the climate change risk.

### Risk assessment

After reviewing existing risk assessment systems, we applied the *Invasiveness Ranking System for Non-native Plants of Alaska* [45] to our study species for Alberta. We selected this risk assessment because it focuses on biodiversity impacts (rather than agricultural impacts), allows assessment of species not yet present in the area of interest, and contains a climate matching pre-screening component to gauge the availability of matching climates between the area of interest and the known species distribution (CLIMEX Regional Match, [46]). Species were assessed using 21 criteria grouped around four attributes: ecological impact, invasive characteristics, dispersal ability, and feasibility of control. Scores received from assessing these four attributes were summed to produce the risk assessment score. We completed risk assessments for each of the 15 species studied (S1 Text, [45]), and our assessments were peer-reviewed by species experts from outside of Alberta. Risk assessment score was calculated based only on known traits with some traits of species unknown. The score ranges from 0–100 with higher scores assigned to species with more invasive characteristics. A score of ≥ 80 categorizes ‘Extremely Invasive’ species, a score of 70–79 categorizes ‘Highly Invasive’ species, and both of these categories are composed of species that are potentially very threatening to Alberta. A score of 60–69 categorizes ‘Moderately Invasive’ species, and a score of 50–59 categorizes ‘Modestly Invasive’ species; both of these categories pose significant risks to ecosystems. A score of 40–49 categorizes ‘Weakly Invasive’ species, and a score < 40 categorizes ‘Very Weakly Invasive’ species. These last 2 categories generally have not been shown to significantly alter ecosystem processes and communities, and do not require as much attention as the other species. (See [45] for further details on how the risk assessment score is calculated).

### Prioritising invasive species for management in a changing climate

We prioritised new invasive species to Alberta using both habitat suitability models under climate change and risk assessments. Using both measures allowed us to identify the worst invaders (from risk assessment) that are predicted to undergo the largest increase in suitable habitat in the province due to climate change (habitat suitability models). Such species were prioritised highly. Other species that possess a low risk assessment score and are predicted to have no increase (or even a decline) in suitable habitat in the province were relatively low on the list of priority invasive species.

## Results

### Habitat suitability

**Fig 2:**
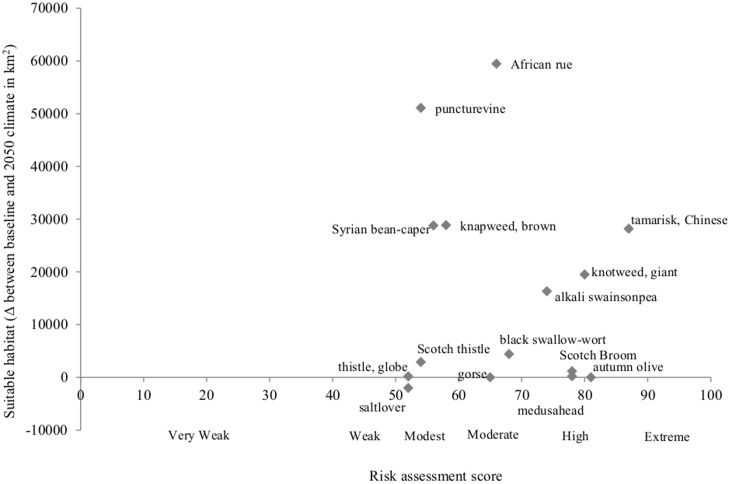
Combining risk assessment and change in suitable high risk habitat between baseline climate and future climate for 15 potentially new invasive species to Alberta.

Projections for most species (14 of 15) showed an increase in *suitable low risk habitat* in Alberta in the 2050s climate compared with 1975 (baseline climate), as well as an increase in *suitable high risk habitat* for 12 of 15 species. Species with the largest increases in suitable *high* risk habitat were: African rue (*Peganum harmala*; 59,472 km^2^) and puncturevine (*Tribulus terrestris*; 51,082 km^2^; Fig 2). Species distribution maps under baseline and future climate for the 15 species are provided in S1 Fig. One species, saltlover (*Halogeton glomeratus*) was projected to have a 46% decline in suitable high risk habitat between the two time periods, perhaps because its distribution was more strongly correlated with precipitation variables than any other species assessed.

Five species were predicted to change from having no *suitable high risk habitat* in Alberta’s current climate to having up to 28,800 km^2^ in *suitable high risk habitat* in the 2050s. These are: Syrian bean-caper (*Zygophyllum fabago*), medusahead (*Taeniatherum caput-medusae*), globe thistle (*Echinops sphaerocephalus*), Scotch broom (*Cytisus scoparius*) and black swallow-wort (*Vincetoxicum nigrum*). Two species, gorse (*Ulex europaeus*) and autumn olive (*Elaeagnus umbellata*), remained without any *suitable high risk habitat* in Alberta in the 2050s, but are predicted to show an increase in *suitable low risk habitat*.

Predictive performance of the habitat suitability models ranged from 0.682 to 0.984, with most species showing excellent discrimination (AUC > 0.9; [29,30]; S1 Table).

### Risk assessment

Tamarisk (*Tamarix chinensis*), autumn olive (*Elaeagnus umbellata*) and giant knotweed (*Fallopia sachalinensis*) were the three species that had the highest risk assessment scores based on their traits and were categorised as ‘extremely invasive’ (sensu [45], Fig 2).

Alkali swainsonpea (*Sphaerophysa salsula*), medusahead (*Taeniatherum caput-medusae*) and Scotch broom (*Cytisus scoparius*) were categorised as ‘highly invasive’. Most of the other species assessed (nine species) had moderate rankings of risk, being categorised as either ‘moderately’ or ‘modestly invasive’. Detailed, peer-reviewed risk assessments based on species traits are in S2 Text. Our risk assessment scores can be applied elsewhere in jurisdictions outside of Alberta that have a similar climate to Alberta, or where there is a similar climate between the existing species range and the jurisdiction in question.

### Using risk assessment and habitat suitability models to prioritise invasive species for management in a changing climate

We examined results from both risk assessment and habitat suitability modeling to prioritise invasive species impacts on biodiversity in a changing climate. Different jurisdictions may place varying levels of emphasis on *risk assessment score* versus *suitable habitat change*, but having both measures to compare will provide the empirical basis on which to prioritise and rank new invasive species that may arrive due to climate change. For Alberta, we prioritised those species that have the highest risk assessment score and are predicted to undergo the largest increase in suitable habitat under climate change (Fig 2). The top three new potential terrestrial invasive plant threats to Alberta were estimated to be Chinese tamarisk (*Tamarix chinensis*), giant knotweed (*Fallopia sachalinensis*), and alkali swainsonpea (*Sphaerophysa salsula*). Chinese tamarisk is ‘extremely invasive’ with a 64% increase in suitable habitat between baseline and future projected climate. Giant knotweed is ‘extremely invasive’ with 21 times the suitable habitat between baseline and future projected climate. Alkali swainsonpea is ‘highly invasive’ with a 21% increase in suitable habitat. These species were all ranked as either ‘extremely’ or ‘highly invasive’ and had the greatest increases in suitable habitat in Alberta between baseline and future projected climate, as projected by habitat suitability models (Fig 2). Relatively low on the priority ranks would be species such as globe thistle (*Echinops sphaerocephalus*), saltlover (*Halogeton glomeratus*) and Scotch thistle (*Onopordum acanthium*) that are modestly invasive and are predicted to have little or no increase in suitable habitat in Alberta due to climate change are relatively low priority invasive species.

## Discussion

Climate change is projected to increase suitable habitat in Alberta for potentially new invasive plant species. The methods we present can be used to predict and prioritize potentially new invasive plant species to an area with a view to formulating pre-emptive management strategies for invasive species in response to climate change [47]. Pre-emptive interventions could include surveillance monitoring to enhance the chances of early detection and rapid response for new invasive species, and targeted localized eradication of high priority invasive species at the early stages of establishment, especially following extreme climate events [1].

We used both risk assessment to categorize species invasive traits, and habitat suitability modeling to quantify the change in suitable habitat projected from climate change for 15 potentially new species to Alberta. We combined both risk assessment and results from habitat suitability modeling to prioritise invasive species impacts on biodiversity. Using either risk assessment, or habitat suitability modeling alone would have produced different, less comprehensive, and perhaps misleading results (Table 2). If only the risk assessment were used to quantify the risk of invasive species, species such as autumn olive that are projected to have little or no suitable high risk habitat in the province would have been prioritized highly. Conversely, if only habitat suitability modeling were used to quantify the risk of invasive species, species such as African rue, brown knapweed and puncturevine that are potentially only modestly to moderately invasive in Alberta would have been prioritized highly. Using both risk assessment and habitat suitability modeling prioritizes the potentially extreme invaders that are projected to have the largest increases in suitable habitat in the province due to climate change.

**Table 2:** Differences in prioritization of new invasive species to Alberta when using risk assessment or habitat suitability modeling alone, compared with combining both risk assessment and habitat suitability modeling.

Risk assessment (trait-based)	Increase in area between baseline and future climate (habitat suitability models)	Combining risk assessment and habitat suitability models
tamarisk, Chinese	African rue	tamarisk, Chinese
autumn olive	puncturevine	knotweed, giant
giant knotweed	knapweed, brown	alkali swainsonpea

Our methods (summarised in Fig 1) are suitable for large scale areas such as provinces or states due to the availability of species records and the scale of environmental data available on climate change. One drawback is that all areas with suitable high risk habitat are treated equally. The intersection of high quality habitat or conservation areas with invasive species distribution models could be used to further distinguish between habitat areas, placing higher priority on those invasive species projected to occupy these important habitats.

### Which non-native species should we consider invasive in the context of biodiversity conservation?

Management of new species that arrive in a jurisdiction as a result of climate change can range from eradication to tolerance to acceptance, and deciding on a management response should be done on a case by case basis, and by prioritizing risk [5]. Blanket removal of non-native species would require increasingly unsustainable efforts and promote ecosystems that are not suitable to emerging climatic conditions [48]. In managing non-native species for conservation purposes under climate change, the management objective should be focused more on managing change by addressing the ‘worst offenders’ that would drive ecological change, than retaining past community composition at all costs [10].

New strategies to cope with invasive species under climate change will include the incorporation of climate change scenarios into planning and management for invasive species, including into risk assessments as illustrated here. Management strategies will also need to be formulated across wider geographic areas (regional perspectives) and longer time frames (e.g., and scenario planning), which require increased coordination across jurisdictions [47]. Data and information on invasive species impacts should be shared across scales and jurisdictions to facilitate risk assessments [7,16,18].

## Conclusion

Climate change in Alberta will result in more suitable habitat for 14 of the 15 potentially new invasive species identified in this report. Of the 15 species assessed, the top three species with the highest risk assessment score which also showed the greatest increase in *suitable habitat* in Alberta due to climate change were: Chinese tamarisk (*Tamarix chinensis*), giant knotweed (*Fallopia sachalinensis*) and alkali swainsonpea (*Sphaerophysa salsula*). Our methodology can be adopted across different jurisdictions to appraise current prediction and prioritization of invasive species for conservation purposes, in the context of climate change.

## Supporting Information

S1 Fig Species distribution models for 15 study species in Alberta in the 1975 (baseline/current climate) and 2050 climate under climate change. (DOCX)

S1 Table Predictive performance of species distribution modeling. An AUC value of 0.5 implies random predictive discrimination, while values above 0.7, 0.8 and 0.9 represent good, very good and excellent discrimination respectively.
(DOCX)

S1 Text Blank Form—Invasiveness ranking system for Alberta. (DOCX)

S2 Text Non-native plant invasiveness ranking form for 15 potentially new invasive species to Alberta. (DOCX)
