# Supplementary material for: Using Risk Assessment and Habitat Suitability Models to Prioritise Invasive Species for Management in a Changing Climate
Source: PLoS One. 2016 Oct 21;11(10):e0165292. doi: 10.1371/journal.pone.0165292 (PMC5074526; doi:10.1371/journal.pone.0165292)

**S1 Fig. Species distribution models for 16 study species in Alberta in the 1975 (baseline/current climate) and 2050 climate under climate change**.
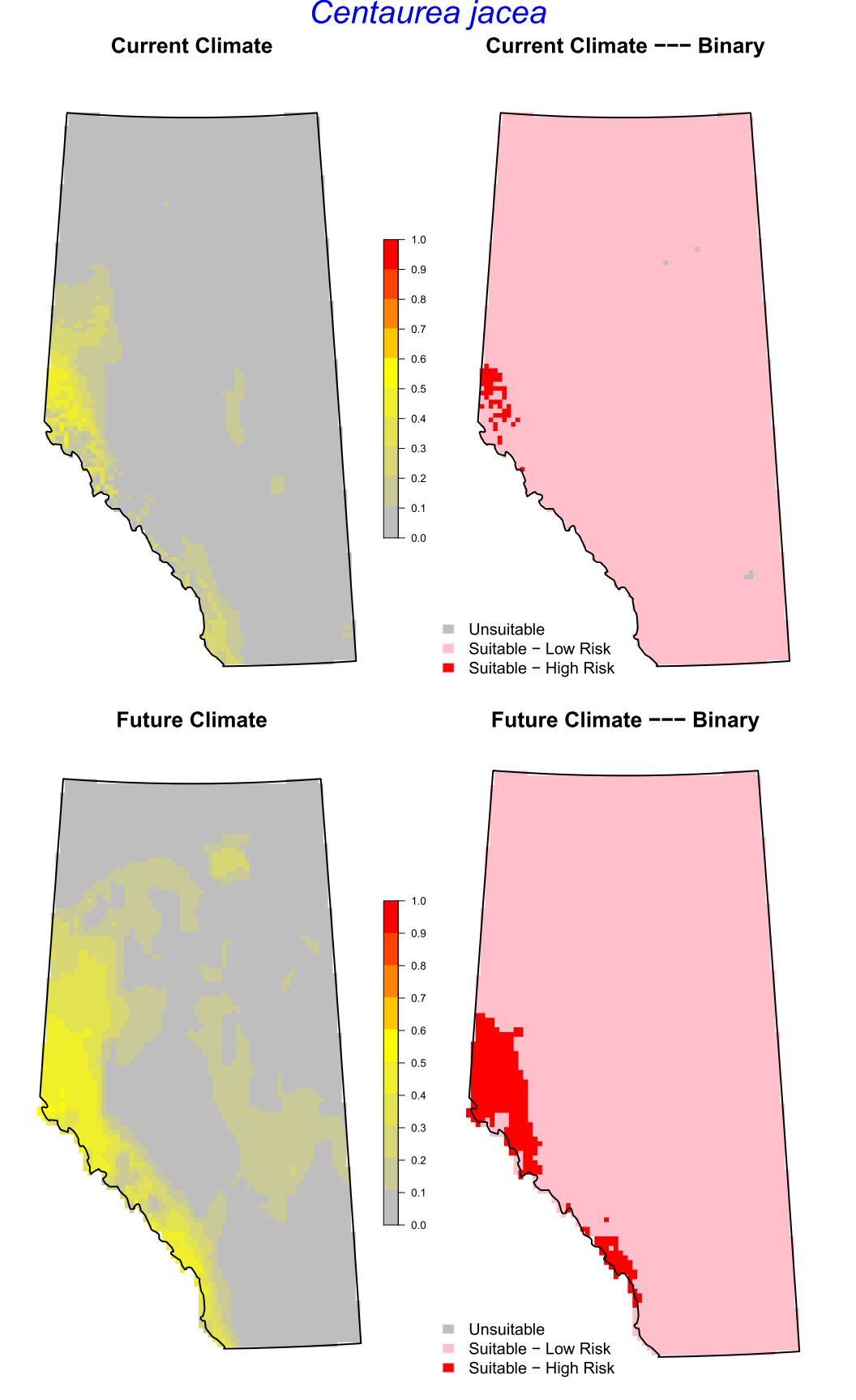

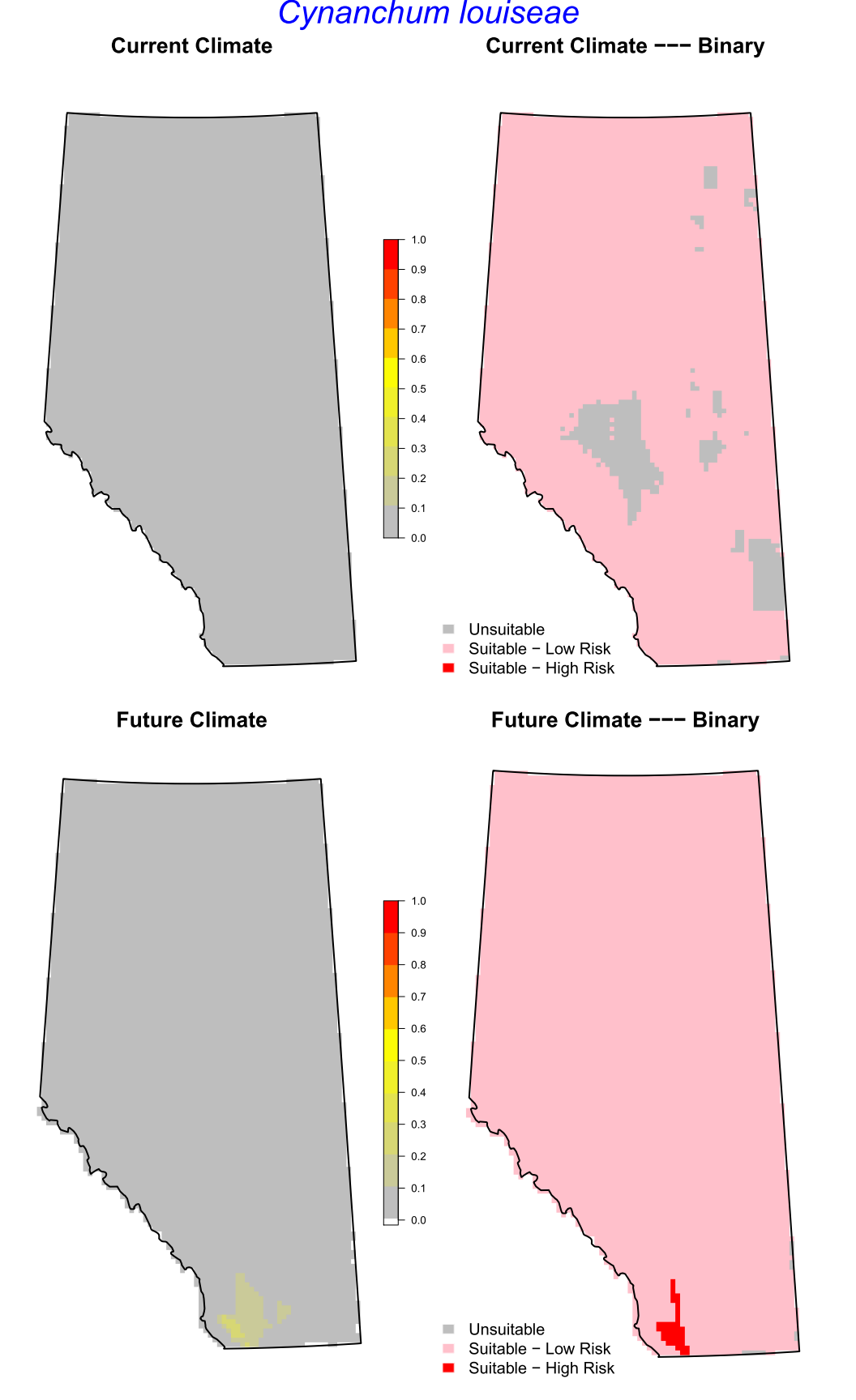

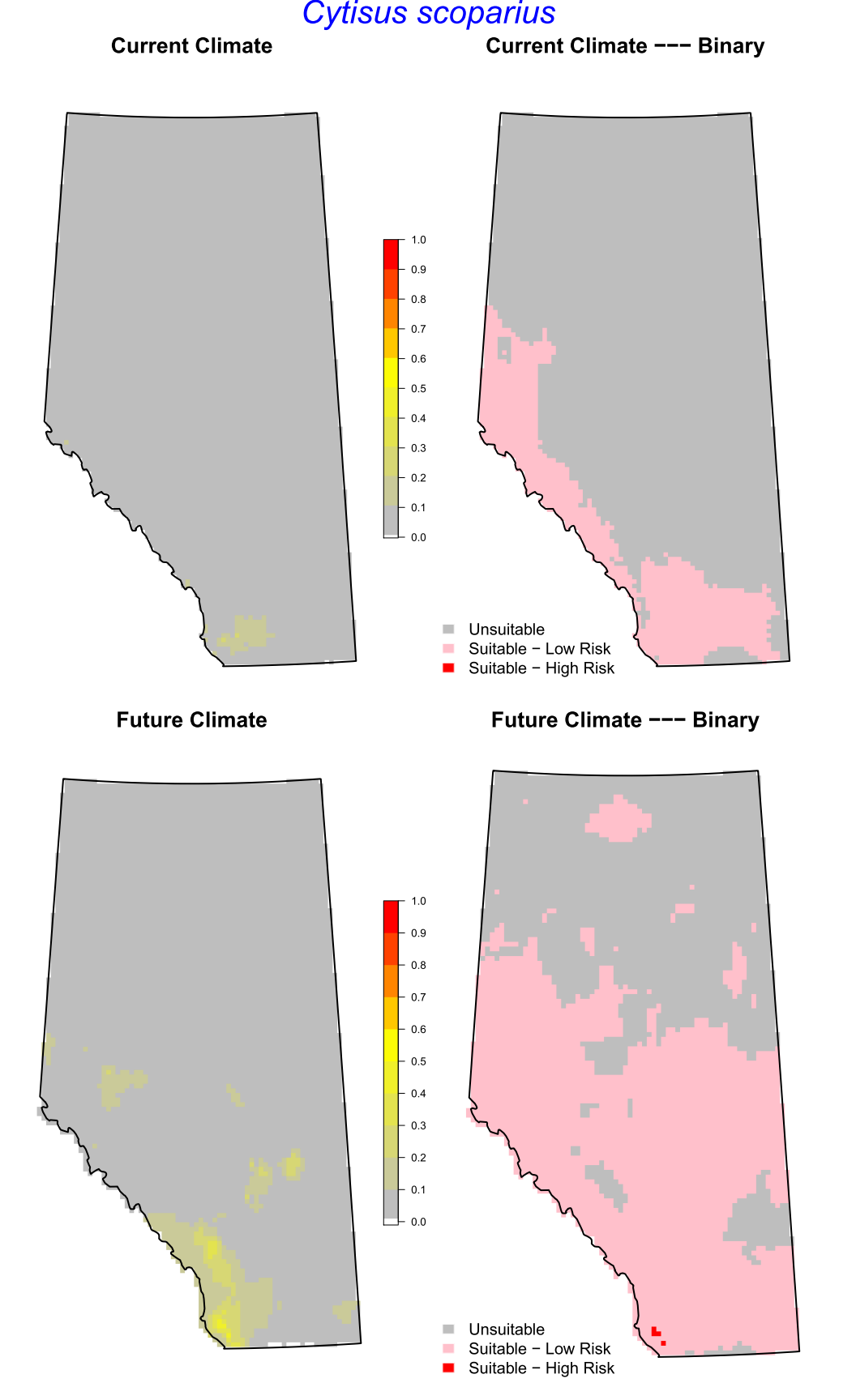

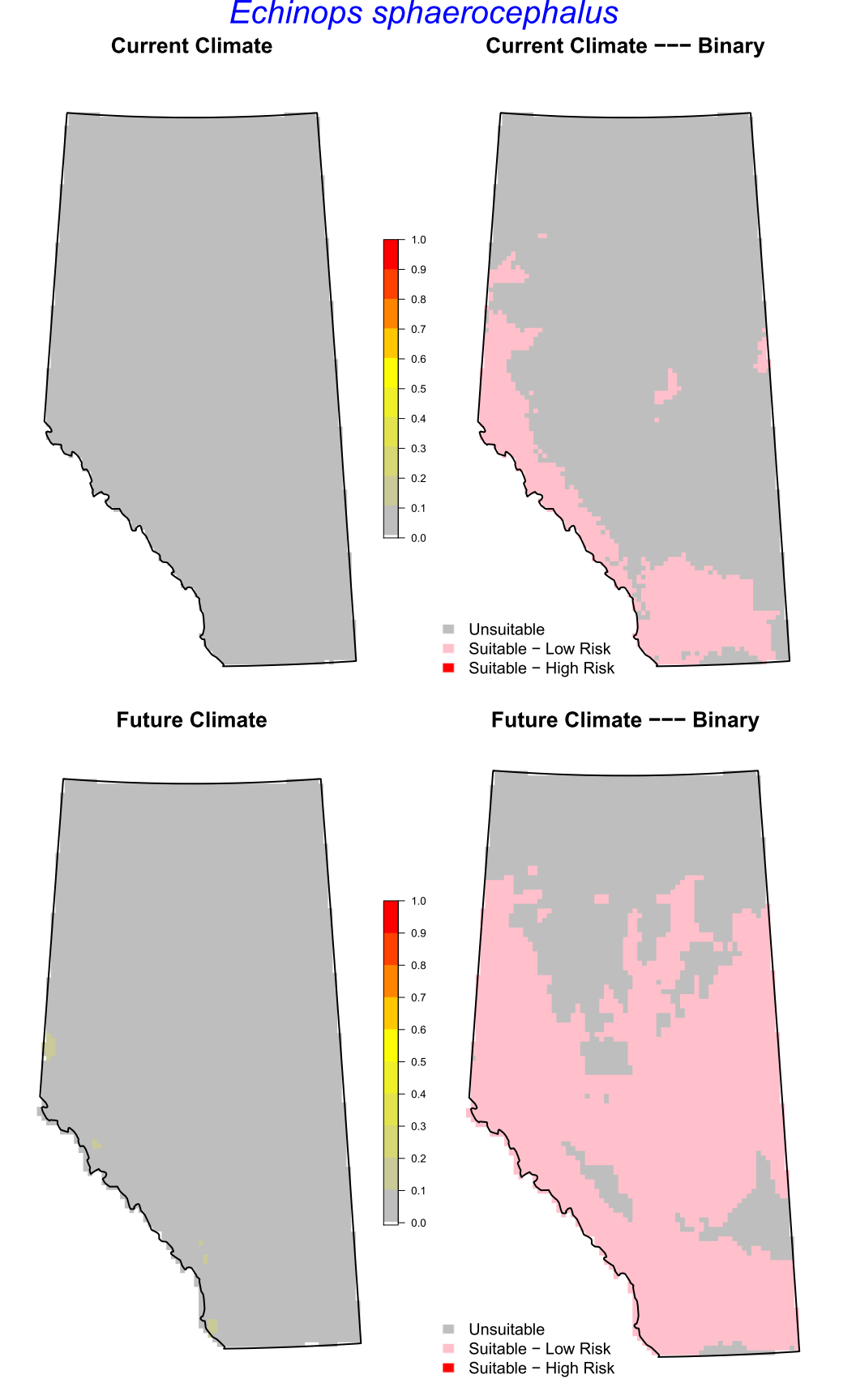

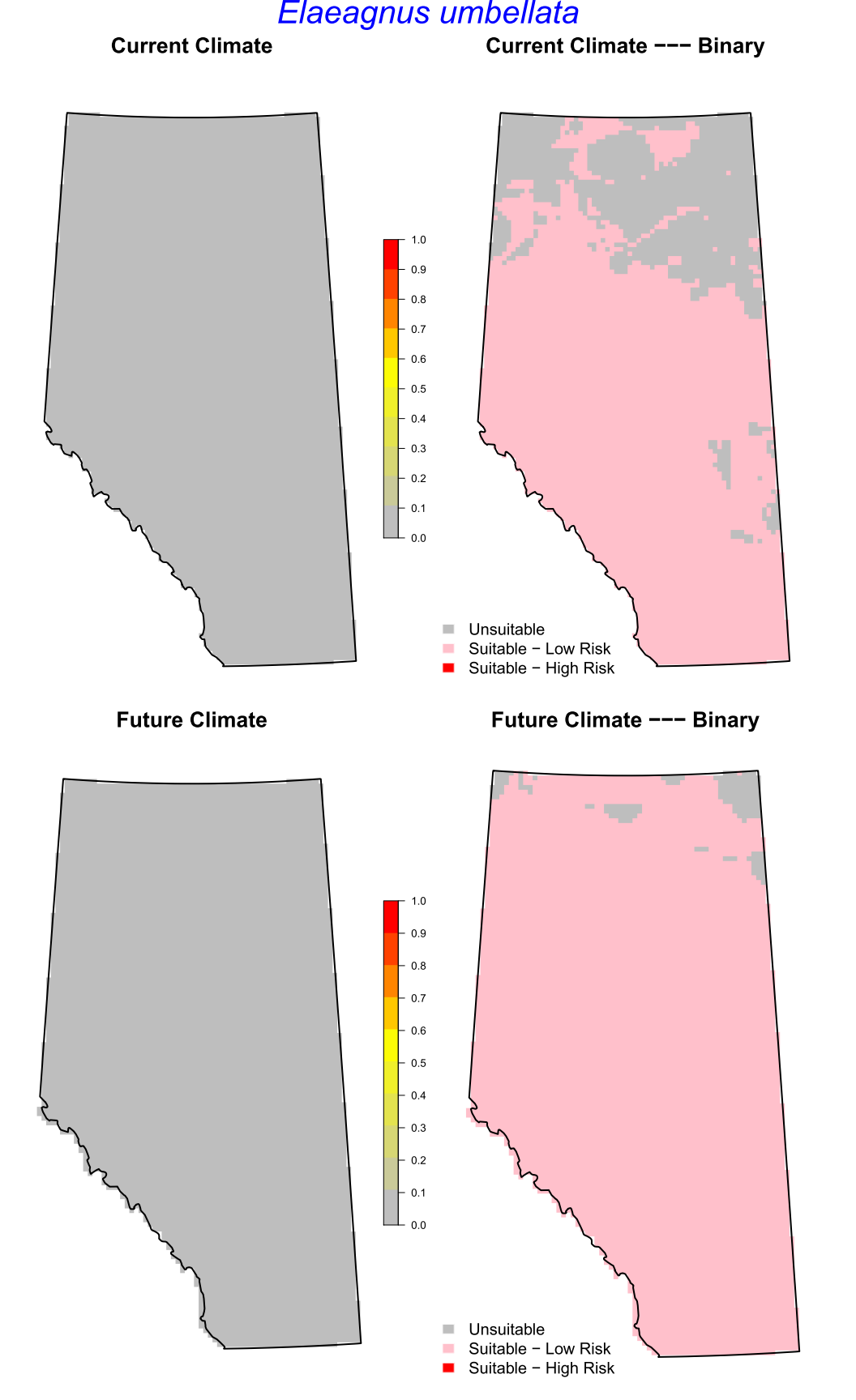

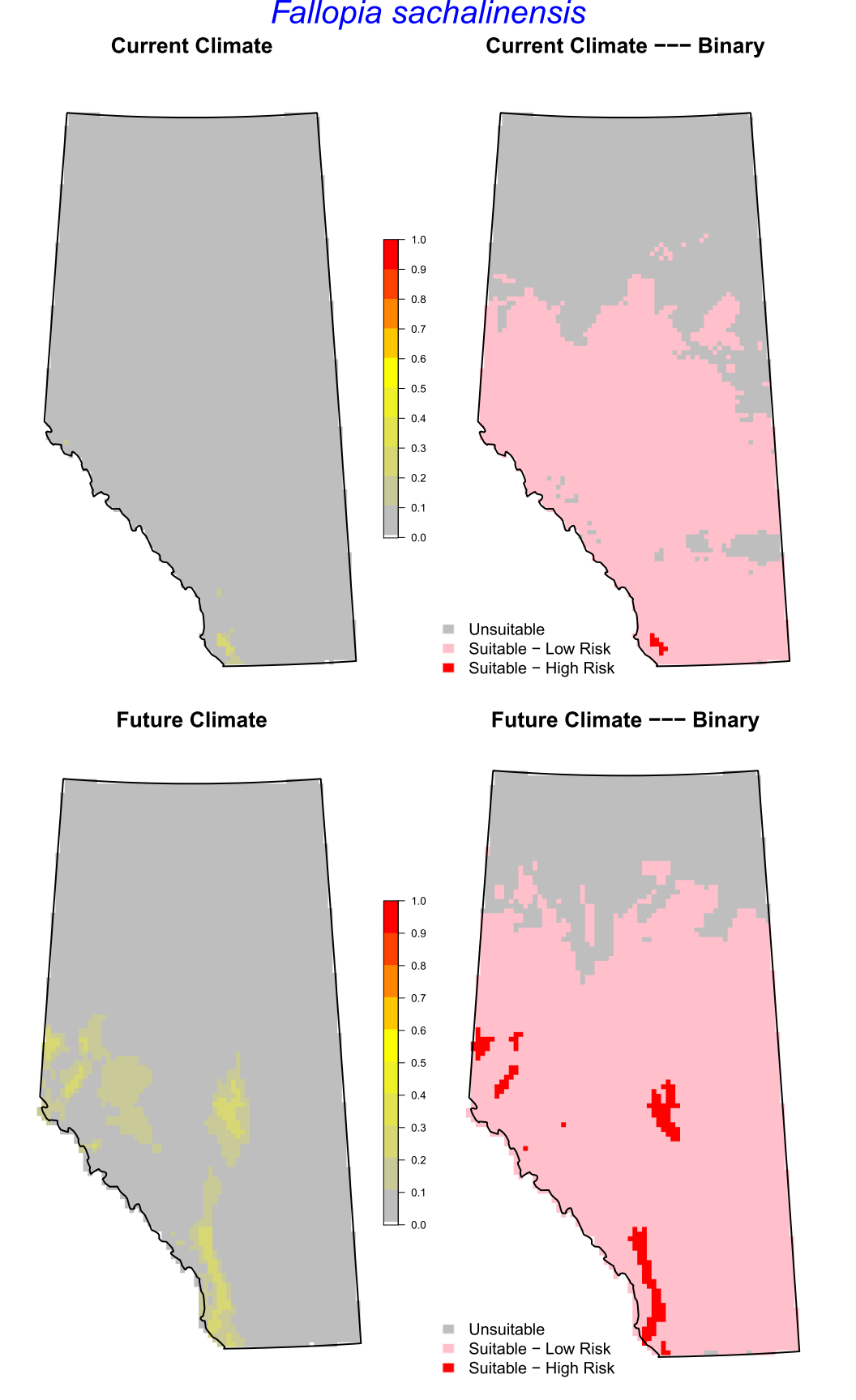

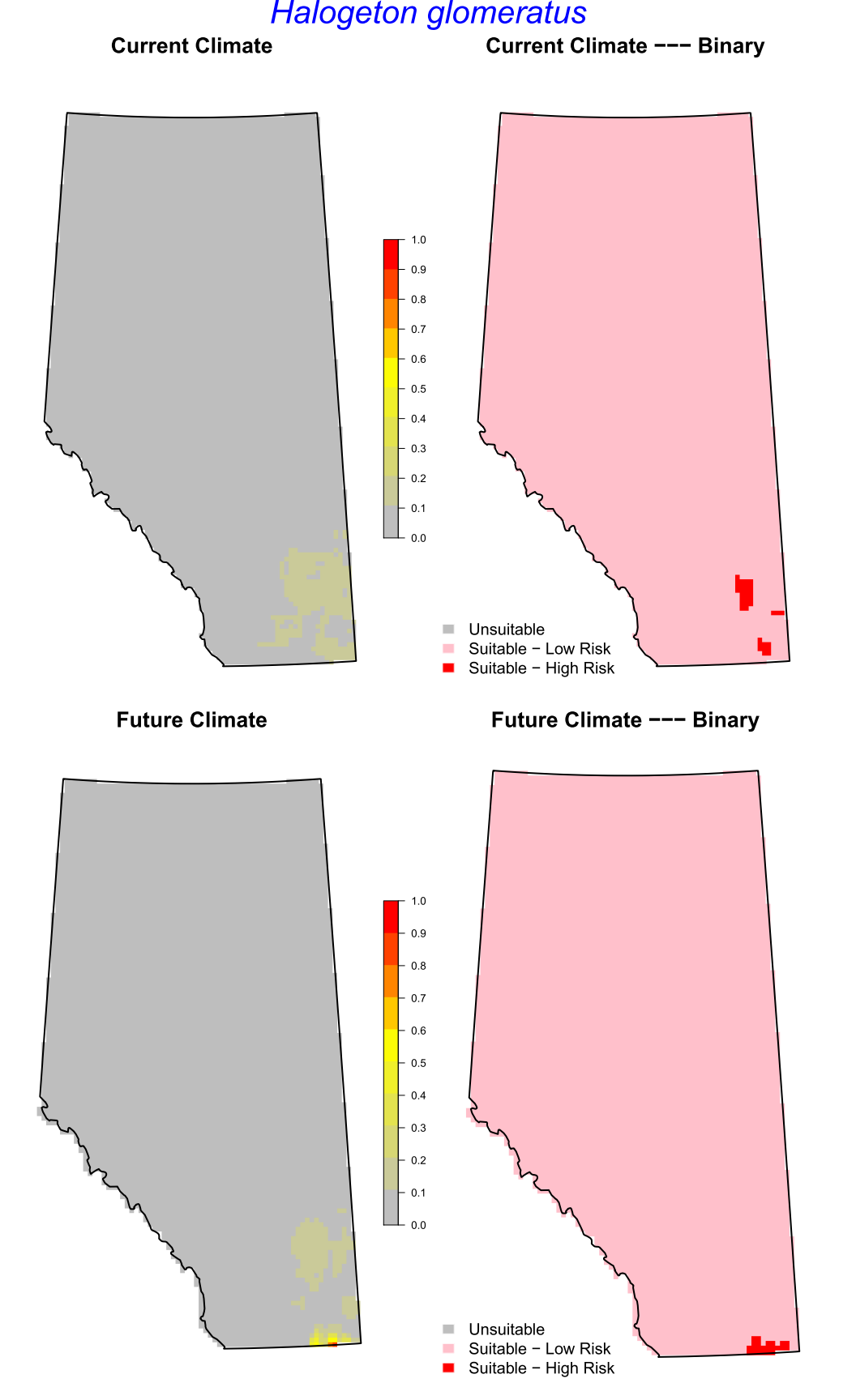

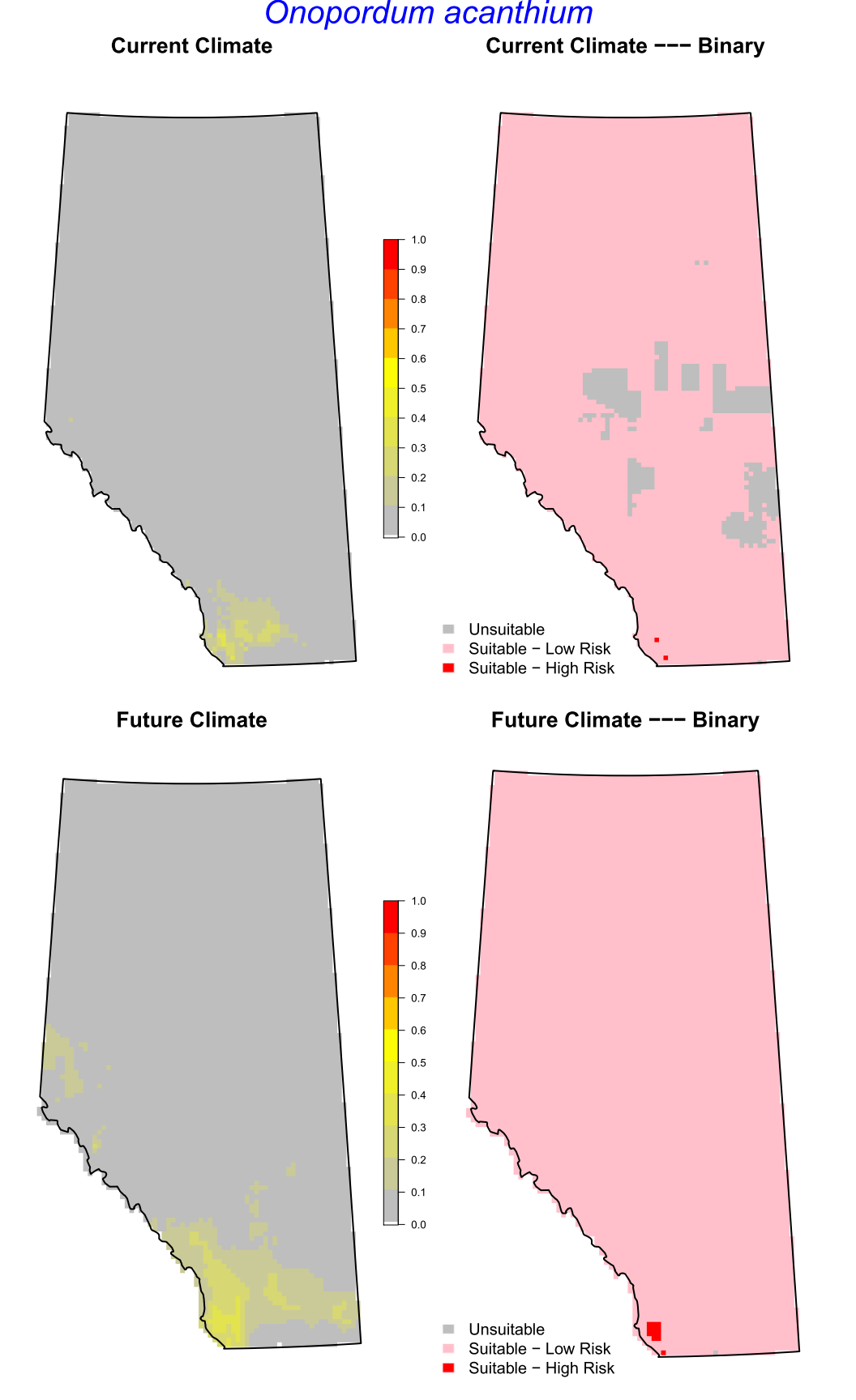

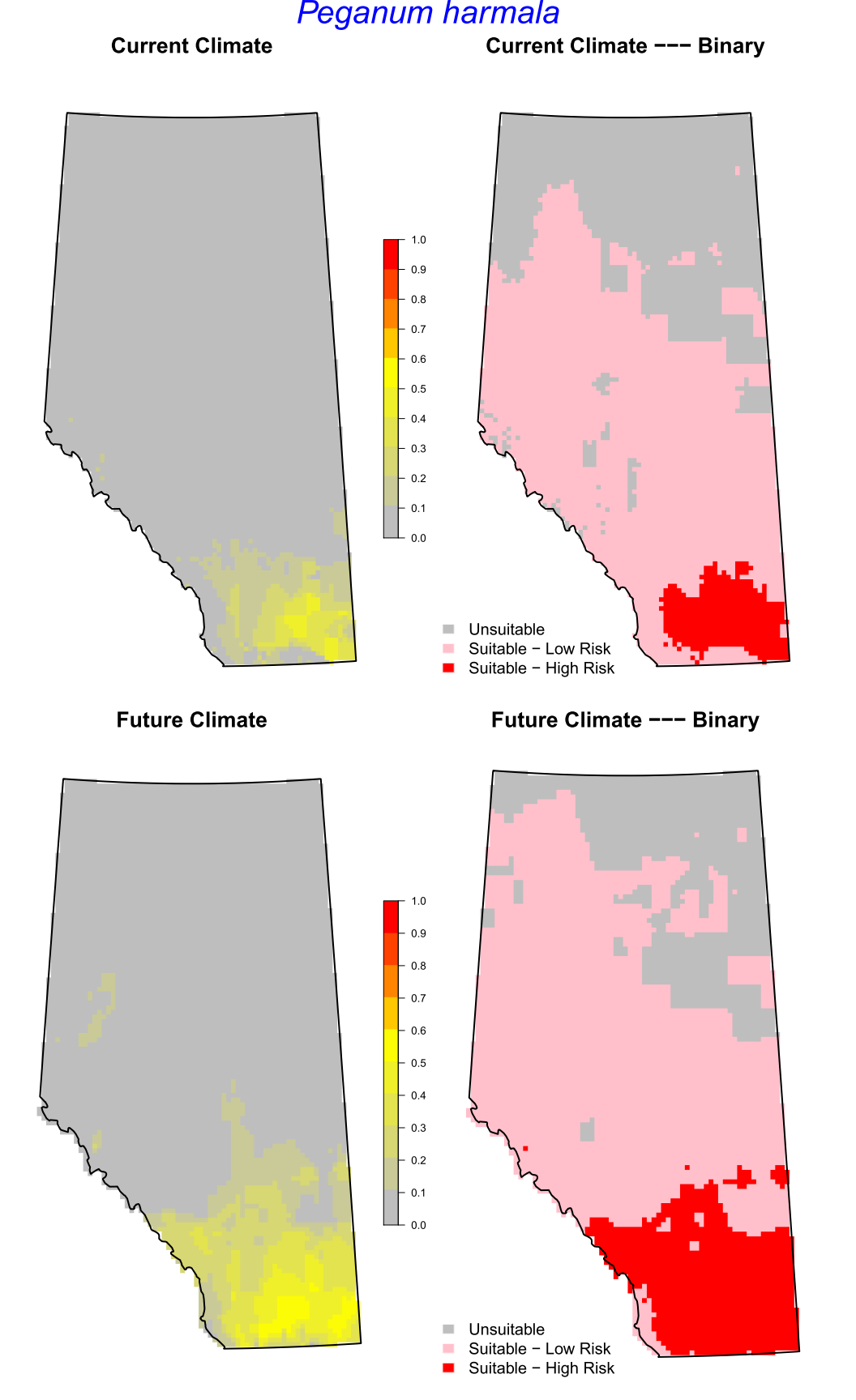

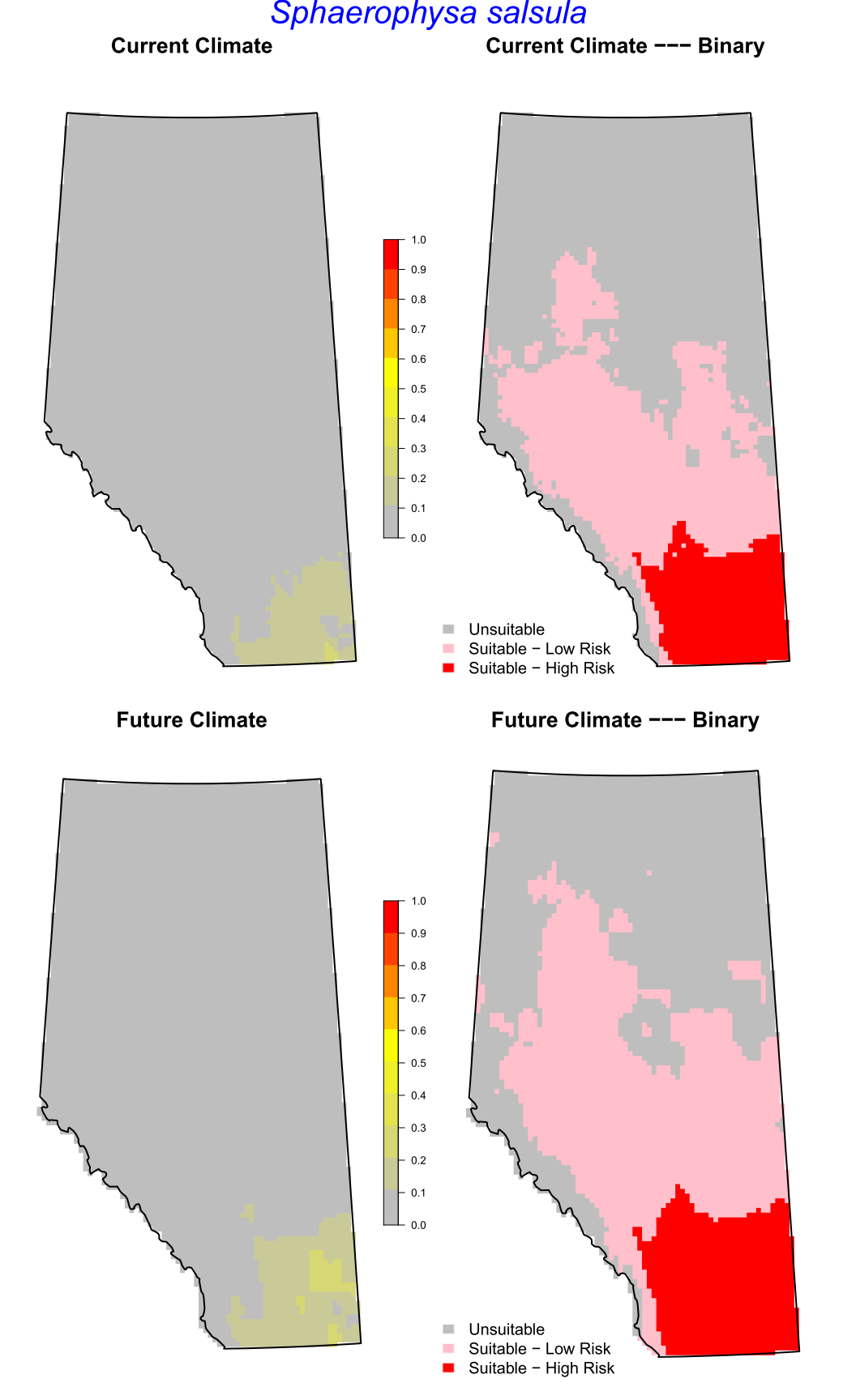

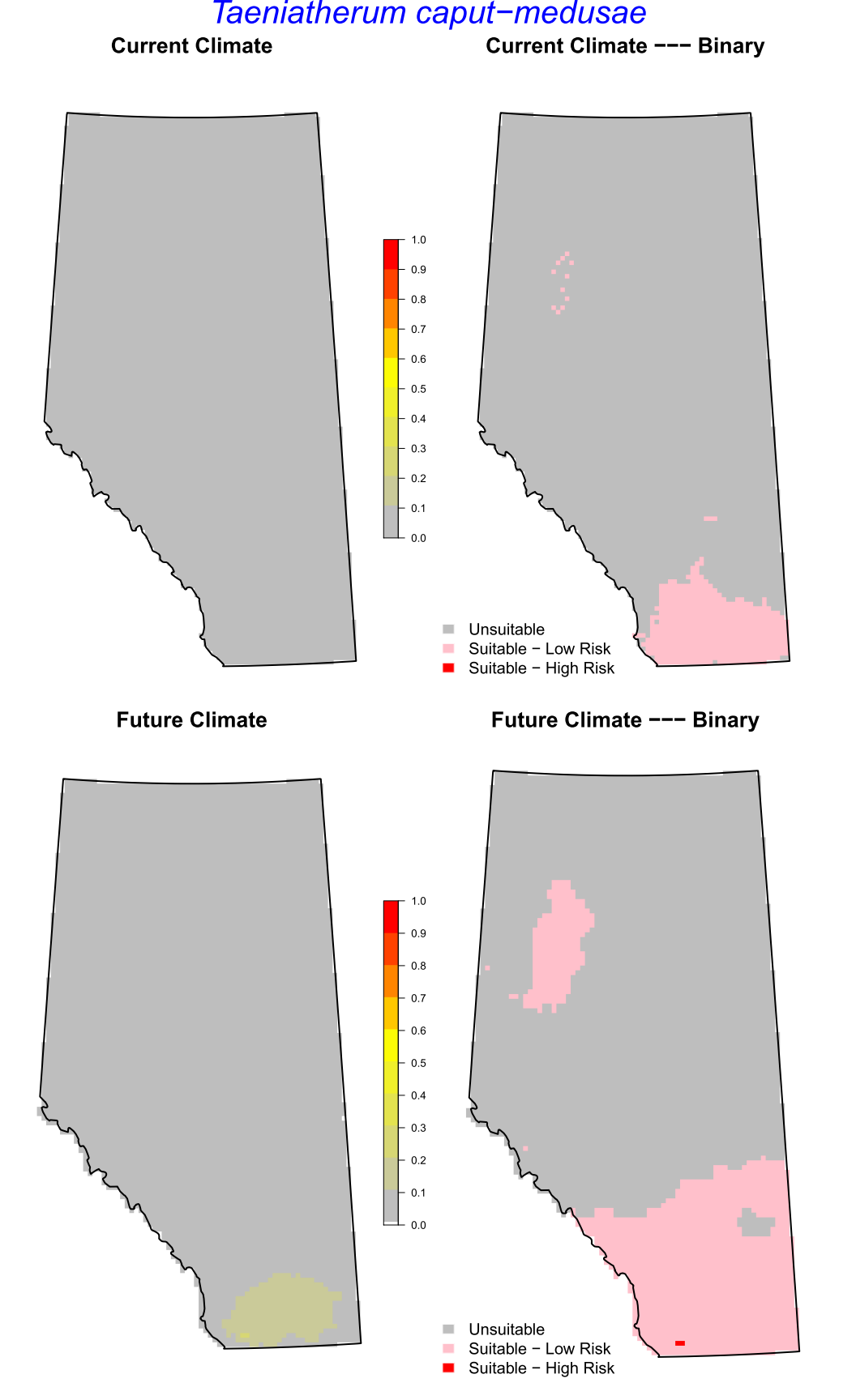

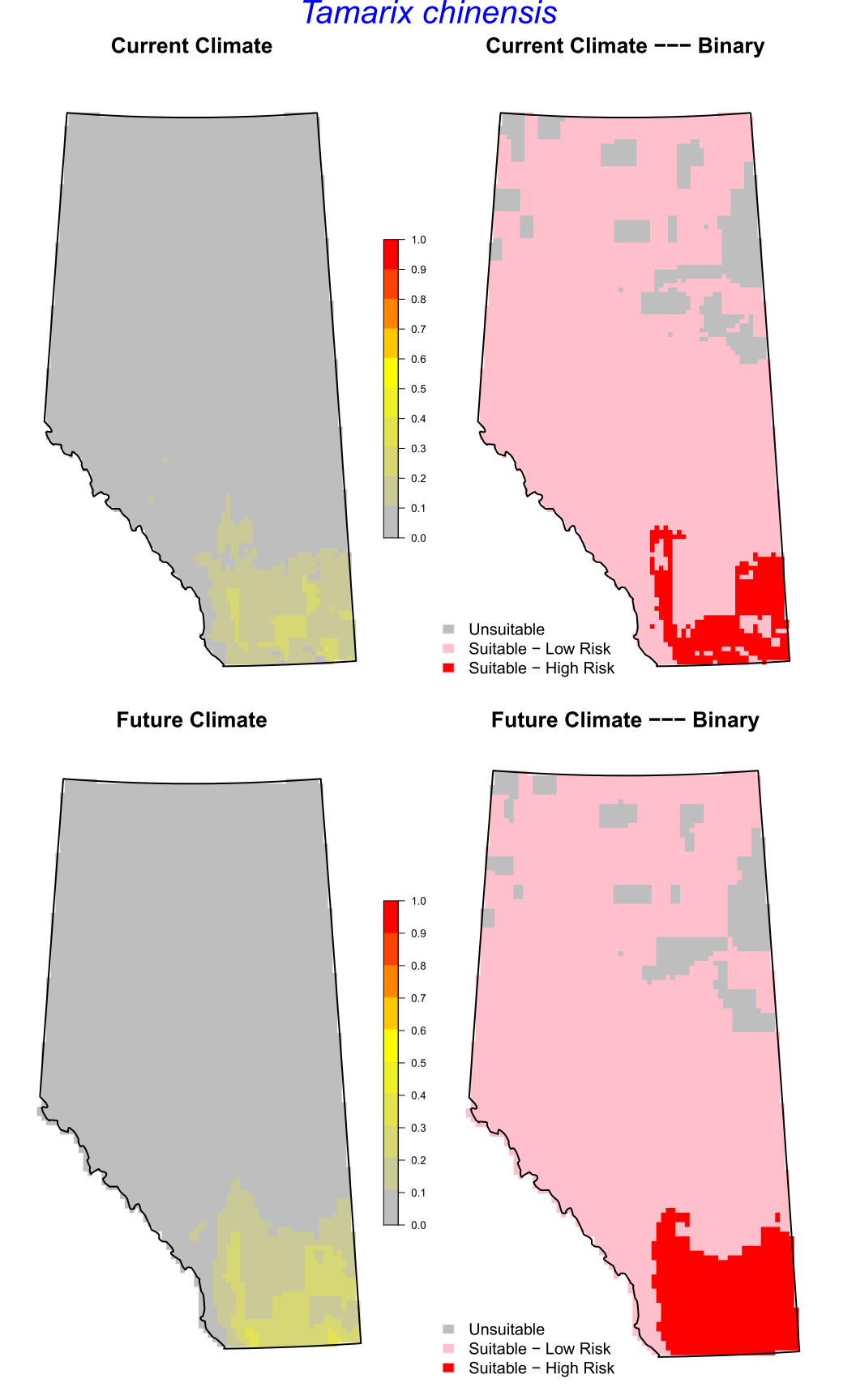

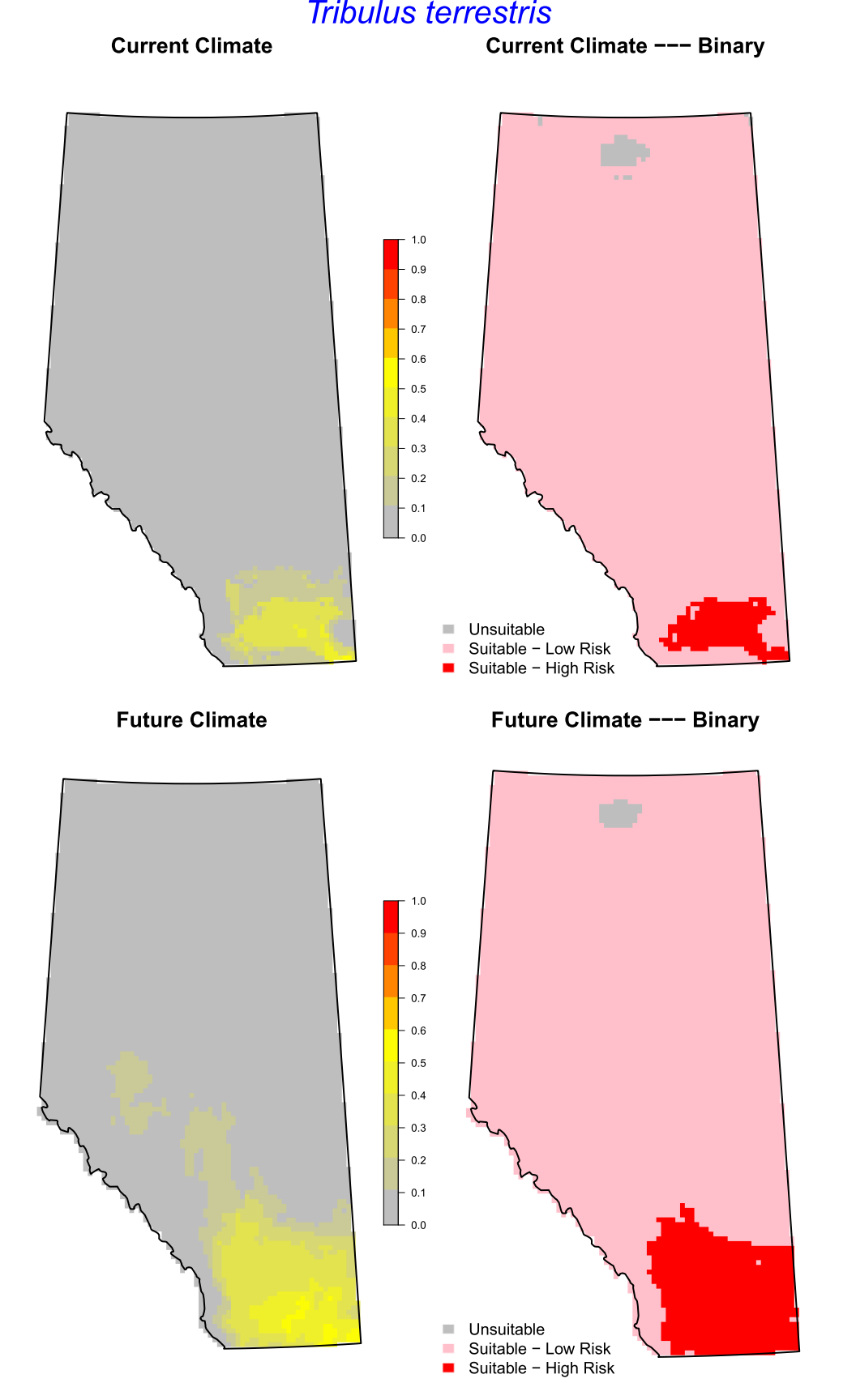

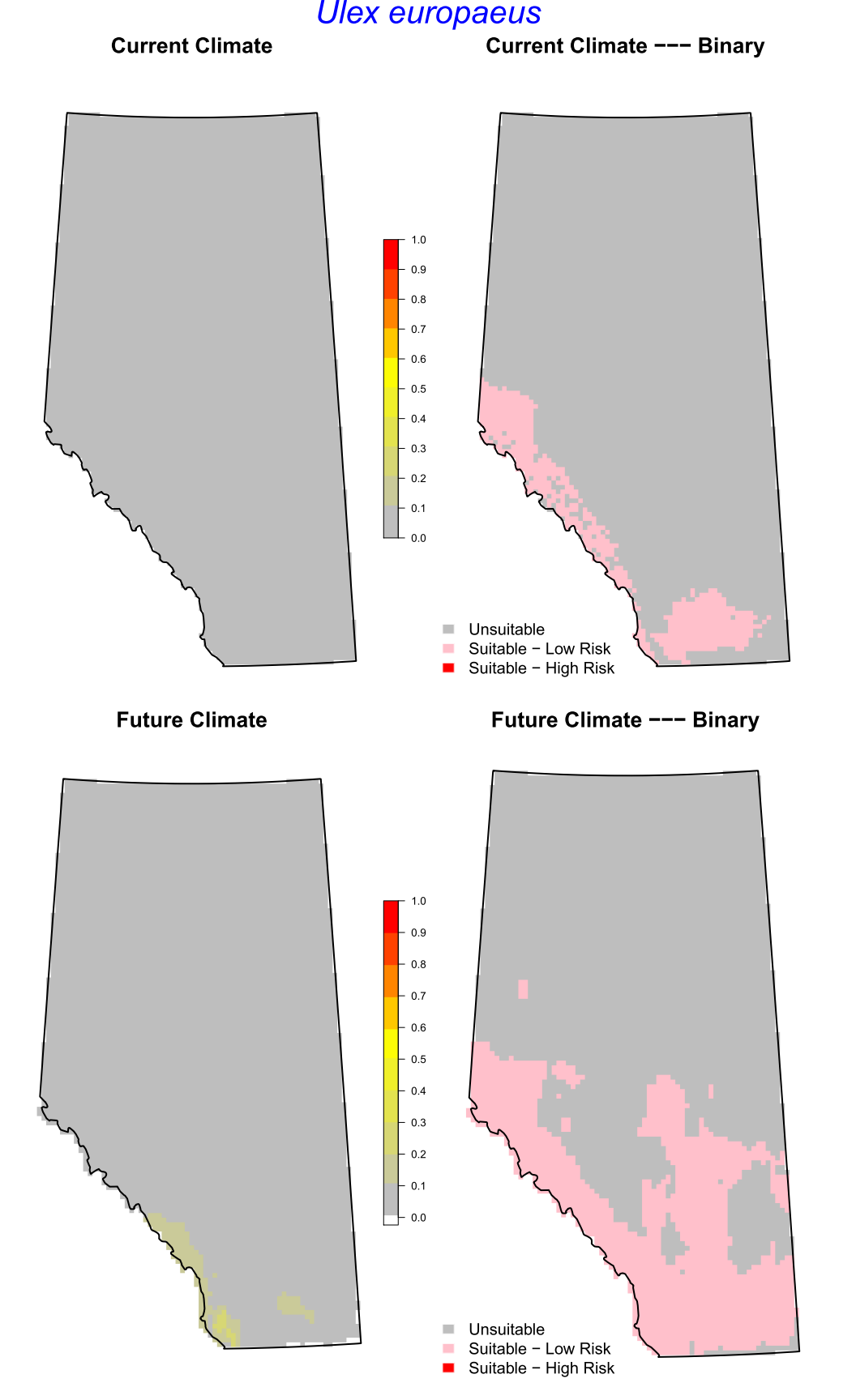

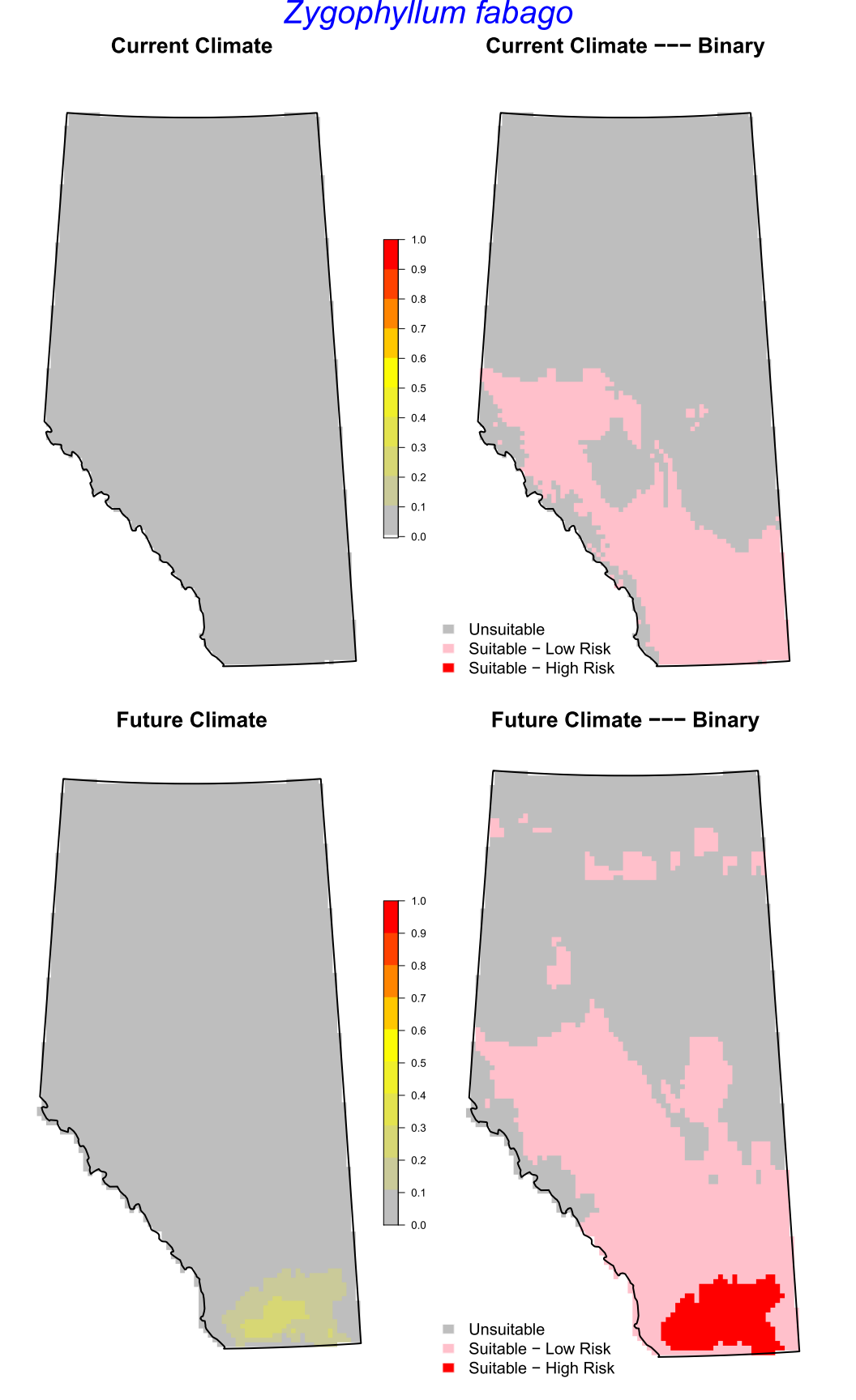

Supplement: S1 Fig — (DOCX) [file pone.0165292.s001.docx]
