# Supplementary material for: Using Risk Assessment and Habitat Suitability Models to Prioritise Invasive Species for Management in a Changing Climate
Source: PLoS One. 2016 Oct 21;11(10):e0165292. doi: 10.1371/journal.pone.0165292 (PMC5074526; doi:10.1371/journal.pone.0165292)
Supplement: S1 Table — An AUC value of 0.5 implies random predictive discrimination, while values above 0.7, 0.8 and 0.9 represent good, very good and excellent discrimination respectively. (DOCX) [file pone.0165292.s002.docx]

**S1 Table. Predictive performance of species distribution modeling. An AUC value of 0.5 implies random predictive discrimination, while values above 0.7, 0.8 and 0.9 represent good, very good and excellent discrimination respectively.**

| Common name | Scientific name | Area under curve (AUC) | |
| --- | --- | --- | --- |
| African rue | *Peganum harmala* | 0.957 |  |
| alkali swainsonpea | *Sphaerophysa salsula* | 0.982 |  |
| autumn olive | *Elaeagnus umbellata* | 0.966 |  |
| black swallow-wort | *Vincetoxicum nigrum* | 0.973 |  |
| gorse | *Ulex europaeus* | 0.769 |  |
| knapweed, brown | *Centaurea jacea* | 0.682 |  |
| knotweed, giant | *Fallopia sachalinensis* | 0.890 |  |
| medusahead | *Taeniatherum caput-medusae* | 0.952 |  |
| puncturevine | *Tribulus terrestris* | 0.945 |  |
| saltlover | *Halogeton glomeratus* | 0.983 |  |
| Scotch broom | *Cytisus scoparius* | 0.695 |  |
| Scotch thistle | *Onopordum acanthium* | 0.823 |  |
| Syrian bean-caper | *Zygophyllum fabago* | 0.984 |  |
| tamarisk, Chinese | *Tamarix chinensis* | 0.962 |  |
| thistle, globe | *Echinops sphaerocephalus* | 0.913 |  |
