# Supplementary material for: Using Risk Assessment and Habitat Suitability Models to Prioritise Invasive Species for Management in a Changing Climate
Source: PLoS One. 2016 Oct 21;11(10):e0165292. doi: 10.1371/journal.pone.0165292 (PMC5074526; doi:10.1371/journal.pone.0165292)
Supplement: S1 Text — (DOCX) [file pone.0165292.s003.docx]

# S1 Text. Blank Form - Invasiveness ranking system for Alberta

**Alberta non-native plant invasiveness ranking form**

(Adapted from Carlson et al. 2008)

| Scientific name: |  |
| --- | --- |
| Common name: |  |
| Assessor: |  |
| Reviewers: |  |
| Date: |  |

Outcome score:

1. Climatic Comparison

This species is present or may potentially establish in the following natural regions:

|  | Collected in Alberta regions | CLIMEX similarity (regional match) in current climate | CLIMEX similarity (regional match) in 2050 |
| --- | --- | --- | --- |
| Boreal |  |  |  |
| Parkland |  |  |  |
| Foothills |  |  |  |
| Grassland |  |  |  |
| Rocky Mountains |  |  |  |
| Shield |  |  |  |

1. Invasiveness Ranking Total (Total answered^1^ points possible) Total score

| 1. Ecological impact | 40() |  |
| --- | --- | --- |
| 1. Biological characteristic and dispersal ability | 25() |  |
| 1. Ecological amplitude and distribution | 25() |  |
| 1. Feasibility of control | 10() |  |
| Outcome score | 100()^b^ | ^a^ |
| Relative maximum score^2^ |  |  |

^1^For questions answered ‘unknown’ do not include point value for the question in parentheses for ‘Total answered points possible.’

^2^Calculated as a/b x 100.

1. Climatic Comparison:
   1. Has this species ever been collected or documented in Alberta?

__Yes – continue to 1.2

__ No – continue to 2.1

1.2 Which natural region has it been collected or documented? Proceed to section B. Invasiveness Ranking.

__Boreal

__Rockies

__Grassland

__Foothills

__Parkland

__Shield

Documentation:

Sources of information:

2.1 Is there a high degree of climate similarity (CMI >0.7) between climates anywhere the species currently occurs and

a. Boreal

b. Rockies

c. Grassland

d. Foothills

e. Parkland

f. Shield

-If ‘no’ is answered for all regions, reject species from consideration

Documentation:

Sources of information:

1. Invasiveness Ranking
2. Ecological Impact
   1. Impact on Natural Ecosystem Processes
3. No perceivable impact on ecosystem processes 0
4. Has the potential to influence ecosystem processes to a minor degree

(e.g., has a perceivable but mild influence on soil nutrient availability) 3

1. Has the potential to cause significant alteration of ecosystem processes (e.g., increases sedimentation rates along streams or coastlines, reduces open water

that are important to waterfowl) 7

1. May cause major, possibly irreversible, alteration or disruption of ecosystem processes (e.g., the species alters geomorphology; hydrology; or affects fire frequency, altering community composition; species fixes substantial levels of nitrogen in the soil making soil unlikely to support certain native plants or more likely to favor non-native species) 10

u. Unknown

Score:

Documentation:

Identify ecosystem processes impacted:

Rationale:

Sources of information:

- 1. Impact on Natural Community Structure

1. No perceived impact; establishes in an existing layer without influencing its

structure 0

1. Has the potential to influence structure in one layer (e.g., changes the density

of one layer) 3

1. Has the potential to cause significant impact in at least one layer (e.g., creation

of a new layer or elimination of an existing layer) 7

1. Likely to cause major alteration of structure (e.g., covers canopy, eradicating

most or all layers below) 10

1. Unknown

Score:

Documentation:

Identify type of impact or alteration:

Rationale:

Sources of information:

- 1. Impact on Natural Community Composition

1. No perceived impact; causes no apparent change in native populations 0
2. Has the potential to influence community composition (e.g., reduces the

number of individuals in one or more native species in the community) 3

1. Has the potential to significantly alters community composition (e.g., produces

a significant reduction in the population size of one or more native species in

the community) 7

1. Likely to cause major alteration in community composition (e.g., results in the extirpation of one or several native species, reducing biodiversity or change the community composition towards species exotic to the natural community) 10

u. Unknown

Score:

Documentation:

Identify type of impact or alteration:

Rationale:

Sources of information:

- 1. Impact on higher trophic levels (cumulative impact of this species on the animals,

fungi, microbes, and other organisms in the community it invades)

1. Negligible perceived impact 0
2. Has the potential to cause minor alteration 3
3. Has the potential to cause moderate alteration (minor reduction in

nesting/foraging sites, reduction in habitat connectivity, interference with

native pollinators, injurious components such as spines, toxins) 7

1. Likely to cause severe alteration of higher trophic populations (extirpation or endangerment of an existing native species/population, or significant reduction

in nesting or foraging sites) 10

u. Unknown

Score:

Documentation:

Identify type of impact or alteration:

Rationale:

Sources of information:

Total Possible:

Total:

1. Biological Characteristics and Dispersal Ability
   1. Mode of reproduction
2. Not aggressive reproduction (few [0-10] seeds per plant and no

vegetative reproduction) 0

1. Somewhat aggressive (reproduces only by seeds (11-1,000/m2) 1
2. Moderately aggressive (reproduces vegetatively and/or by a moderate

amount of seed, <1,000/m2) 2

1. Highly aggressive reproduction (extensive vegetative spread and/or

many seeded, >1,000/m2) 3

u. Unknown

Score:

Documentation:

Describe key reproductive characteristics (including seeds per plant):

Rationale:

Sources of information:

- 1. Innate potential for long-distance dispersal (bird dispersal, sticks to animal hair, buoyant fruits, wind-dispersal)

1. Does not occur (no long-distance dispersal mechanisms) 0
2. Infrequent or inefficient long-distance dispersal (occurs occasionally

despite lack of adaptations) 2

1. Numerous opportunities for long-distance dispersal (species has

adaptations such as pappus, hooked fruit-coats, etc.) 3

1. Unknown

Score:

Documentation:

Identify dispersal mechanisms:

Rationale:

Sources of information:

- 1. Potential to be spread by human activities (both directly and indirectly – possible mechanisms include: commercial sales, use as forage/revegetation, spread along highways, transport on boats, contamination, etc.)

1. Does not occur 0
2. Low (human dispersal is infrequent or inefficient) 1
3. Moderate (human dispersal occurs) 2
4. High (there are numerous opportunities for dispersal to new areas) 3

u. Unknown

Score:

Documentation:

Identify dispersal mechanisms:

Rationale:

Sources of information:

- 1. Allelopathic

1. no 0
2. yes 2
3. unknown

Score:

Documentation:

Describe effect on adjacent plants:

Rationale:

Sources of information:

- 1. Competitive ability

a. Poor competitor for limiting factors 0

b. Moderately competitive for limiting factors 1

c. Highly competitive for limiting factors and/or nitrogen fixing ability 3

u. Unknown

Score:

Documentation:

Evidence of competitive ability:

Rationale:

Sources of information:

- 1. Forms dense thickets, climbing or smothering growth habit, or otherwise taller than the surrounding vegetation

1. No 0
2. Forms dense thickets 1
3. Has climbing or smothering growth habit, or otherwise taller than the surrounding

vegetation 2

u. Unknown

Score:

Documentation:

Describe growth form:

Rationale:

Sources of information:

- 1. Germination requirements

1. Requires open soil and disturbance to germinate 0
2. Can germinate in vegetated areas but in a narrow range or in special conditions 2
3. Can germinate in existing vegetation in a wide range of conditions 3

u. Unknown

Score:

Documentation:

Describe germination requirements:

Rationale:

Sources of information:

- 1. Other species in the genus invasive in Alberta or elsewhere

1. No 0
2. Yes 3

u. Unknown

Score:

Documentation:

Species:

Sources of information:

2.9 Aquatic, wetland, or riparian species

a. Not invasive in wetland communities 0

b. Invasive in riparian communities 1

c. Invasive in wetland communities 3

u. Unknown

Score:

Documentation:

Describe type of habitat:

Rationale:

Sources of information:

Total Possible:

Total:

1. Distribution
   1. Is the species highly domesticated or a weed of agriculture
2. No 0
3. Is occasionally an agricultural pest 2
4. Has been grown deliberately, bred, or is known as a significant agricultural pest 4
5. Unknown

Score:

Documentation:

Identify reason for selection, or evidence of weedy history:

Rationale:

Sources of information:

- 1. Known level of ecological impact in natural areas

1. Not known to cause impact in any other natural area 0
2. Known to cause impacts in natural areas, but in dissimilar habitats and

climate zones than exist in regions of Alberta 1

1. Known to cause low impact in natural areas in similar habitats and climate

zones to those present in Alberta 3

1. Known to cause moderate impact in natural areas in similar habitat and

climate zones 4

1. Known to cause high impact in natural areas in similar habitat and climate

zones 6

u. Unknown

Score:

Documentation:

Identify type of habitat and states or provinces where it occurs:

Sources of information:

- 1. Role of anthropogenic and natural disturbance in establishment

1. Requires anthropogenic disturbances to establish 0
2. May occasionally establish in undisturbed areas but can readily establish in

areas with natural disturbances 3

1. Can establish independent of any known natural or anthropogenic disturbances 5

u. Unknown

Score:

Documentation:

Identify type of disturbance:

Rationale:

Sources of information:

- 1. Current global distribution

1. Occurs in one or two continents or regions (e.g., Mediterranean region) 0
2. Extends over three or more continents 3
3. Extends over three or more continents, including successful introductions in

arctic or subarctic regions 5

u. Unknown

Score:

Documentation:

Describe distribution:

Rationale:

Sources of information:

- 1. Extent of the species Canada range and/or occurrence of formal state or provincial listing

1. 0-5 percent of the states/provinces 0
2. 6-20 percent of the states/provinces 2
3. 21-50 percent, and/or state/province listed as a problem weed

(e.g., ‘Noxious,’ or ‘Invasive’) in 1 state or Canadian province 4

1. Greater than 50 percent, and/or identified as ‘Noxious’ in 2 or more states or

Canadian provinces 5

u. Unknown

Score:

Documentation:

Identify provinces invaded:

Rationale:

Sources of information:

Total possible:

Total:

1. Feasibility of Control
   1. Seed banks
2. Seeds remain viable in the soil for less than 3 years 0
3. Seeds remain viable in the soil for between 3 and 5 years 2
4. Seeds remain viable in the soil for 5 years and more 3

u. Unknown

Score:

Documentation:

Identify longevity of seed bank

Rationale:

Sources of information:

- 1. Vegetative regeneration

1. No resprouting following removal of aboveground growth 0
2. Resprouting from ground-level meristems 1
3. Resprouting from extensive underground system 2
4. Any plant part is a viable propagule 3

u. Unknown

Score:

Documentation:

Describe vegetative response:

Rationale:

Sources of information:

- 1. Level of effort required

1. Management is not required (e.g., species does not persist without repeated anthropogenic disturbance) 0
2. Management is relatively easy and inexpensive; requires a minor investment in human and financial resources 2
3. Management requires a major short-term investment of human and financial resources, or a moderate long-term investment 3
4. Management requires a major, long-term investment of human and financial resources 4

u. Unknown

Score:

Documentation:

Identify types of control methods and time-term required:

Rationale:

Sources of information:

Total Possible:

Total:

Total for 4 sections Possible:

Total for 4 sections:

References:

Notes:

Score Interpretation:

While different users will have different concepts of what constitutes various levels of invasiveness (e.g., what is ‘highly invasive’ vs. ‘moderately invasive’ may differ among management agencies), we divided the ranks into six blocks in Appendix A. We consider species with scores ≥80 as ‘Extremely Invasive’ and species with scores 70–79 as ‘Highly Invasive;’ both of these groups are composed of species estimated to be very threaten­ing to Alberta. Species with scores of 60–69 as ‘Moderately Invasive’ and scores of 50–59 represent ‘Modestly Invasive’ species; both of these groups still pose significant risks to ecosystems. Species with scores of 40–49 are ‘Weakly Invasive’, and <40 are considered ‘Very Weakly Invasive.’ These last two groups generally have not been shown to significantly alter ecosystem processes and communities elsewhere and probably do not require as much attention as the other species.
