# Supplementary material for: Using Risk Assessment and Habitat Suitability Models to Prioritise Invasive Species for Management in a Changing Climate
Source: PLoS One. 2016 Oct 21;11(10):e0165292. doi: 10.1371/journal.pone.0165292 (PMC5074526; doi:10.1371/journal.pone.0165292)
Supplement: S2 Text — (DOCX) [file pone.0165292.s004.docx]

**S2 Text. Non-native plant invasiveness ranking form for 15 potentially new invasive species to Alberta**

(Adapted from Carlson et al. 2008)

| Scientific name: | *Peganum harmala* |
| --- | --- |
| Common name: | African Rue, Syrian-rue, wild rue, harmal |
| Assessor: | Shauna-Lee Chai |
| Reviewer: | John Kartesz |
| Date: | October 25, 2013 |

Outcome score:

1. Climatic Comparison

This species is present or may potentially establish in the following natural regions:

|  | Collected in Alberta regions | CLIMEX similarity in 1975 | CLIMEX similarity in 2050 |
| --- | --- | --- | --- |
| Boreal | No | 0.752 | 0.779 |
| Parkland | No | 0.797 | 0.852 |
| Foothills | No | 0.821 | 0.829 |
| Grassland | No | 0.840 | 0.874 |
| Rocky Mountains | No | 0.786 | 0.777 |
| Shield | No | 0.701 | 0.733 |

1. Invasiveness Ranking Total (Total answered^1^ points possible) Total score

| 1. Ecological impact | 40(40) | 27 |
| --- | --- | --- |
| 1. Biological characteristic and dispersal ability | 25(25) | 15 |
| 1. Ecological amplitude and distribution | 25(25) | 16 |
| 1. Feasibility of control | 10(7) | 6 |
| Outcome score | 100(97)^b^ | ^a^64 |
| Relative maximum score^2^ | 66 | *Moderately Invasive* |

^1^For questions answered “unknown” do not include point value for the question in parentheses for “Total answered points possible.”

^2^Calculated as a/b x 100.

1. Climatic Comparison:
   1. Has this species ever been collected or documented in Alberta?

__Yes – continue to 1.2

x No – continue to 2.1

1.2 Which natural region has it been collected or documented? Proceed to section B. Invasiveness Ranking.

__Boreal

__Rockies

__Grassland

__Foothills

__Parkland

__Shield

Documentation:

Sources of information: ANPC Rogues gallery, ACIMS, PLANTS database, GBIF

2.1 Is there a 70 percent or higher similarity (based on CLIMEX climate matching) between climates anywhere the species currently occurs and

a. Boreal - Yes

b. Rockies - Yes

c. Grassland - Yes

d. Foothills - Yes

e. Parkland - Yes

f. Shield - Yes

-If “no” is answered for all regions, reject species from consideration

Documentation:

Sources of information:

1. Invasiveness Ranking
2. Ecological Impact
   1. Impact on Natural Ecosystem Processes
3. No perceivable impact on ecosystem processes 0
4. Has the potential to influence ecosystem processes to a minor degree

(e.g., has a perceivable but mild influence on soil nutrient availability) 3

1. Has the potential to cause significant alteration of ecosystem processes (e.g., increases sedimentation rates along streams or coastlines, reduces open water

that are important to waterfowl) 7

1. May cause major, possibly irreversible, alteration or disruption of ecosystem processes (e.g., the species alters geomorphology; hydrology; or affects fire frequency, altering community composition; species fixes substantial levels of nitrogen in the soil making soil unlikely to support certain native plants or more likely to favor non-native species) 10

u. Unknown

Score:3

Documentation: acts primarily on degraded rangelands and waste areas along roadsides. Can crowd out native species (CFIA 2010)

Identify ecosystem processes impacted:

Rationale:

Sources of information:

- 1. Impact on Natural Community Structure

1. No perceived impact; establishes in an existing layer without influencing its

structure 0

1. Has the potential to influence structure in one layer (e.g., changes the density

of one layer) 3

1. Has the potential to cause significant impact in at least one layer (e.g., creation

of a new layer or elimination of an existing layer) 7

1. Likely to cause major alteration of structure (e.g., covers canopy, eradicating

most or all layers below) 10

1. Unknown

Score: 7

Documentation: Can become dominant (Abbott et al. 2007)

Identify type of impact or alteration:

Rationale:

Sources of information:

- 1. Impact on Natural Community Composition

1. No perceived impact; causes no apparent change in native populations 0
2. Has the potential to influence community composition (e.g., reduces the

number of individuals in one or more native species in the community) 3

1. Has the potential to significantly alters community composition (e.g., produces

a significant reduction in the population size of one or more native species in

the community) 7

1. Likely to cause major alteration in community composition (e.g., results in the extirpation of one or several native species, reducing biodiversity or change the community composition towards species exotic to the natural community) 10

u. Unknown

Score:10

Documentation: invades dry grasslands and can become dominant (Abott et al. 2007)

Identify type of impact or alteration:

Rationale:

Sources of information:

- 1. Impact on higher trophic levels (cumulative impact of this species on the animals,

fungi, microbes, and other organisms in the community it invades)

1. Negligible perceived impact 0
2. Has the potential to cause minor alteration 3
3. Has the potential to cause moderate alteration (minor reduction in

nesting/foraging sites, reduction in habitat connectivity, interference with

native pollinators, injurious components such as spines, toxins) 7

1. Likely to cause severe alteration of higher trophic populations (extirpation or endangerment of an existing native species/population, or significant reduction

in nesting or foraging sites) 10

u. Unknown

Score:7

Documentation: Toxic to livestock-horses, sheep, cattle and unsuitable forage to native grazers (Abbott et al. 2007).

Identify type of impact or alteration:

Rationale:

Sources of information:

Total Possible:40

Total:27

1. Biological Characteristics and Dispersal Ability
   1. Mode of reproduction
2. Not aggressive reproduction (few [0-10] seeds per plant and no

vegetative reproduction) 0

1. Somewhat aggressive (reproduces only by seeds (11-1,000/m2) 1
2. Moderately aggressive (reproduces vegetatively and/or by a moderate

amount of seed, <1,000/m2) 2

1. Highly aggressive reproduction (extensive vegetative spread and/or

many seeded, >1,000/m2) 3

u. Unknown

Score: 3

Documentation: Flowers have 45-60 seeds with each plant producing up to 1000 fruits and it reproduces vegetatively (USDA 2012)

Describe key reproductive characteristics (including seeds per plant):

Rationale:

Sources of information:

- 1. Innate potential for long-distance dispersal (bird dispersal, sticks to animal hair, buoyant fruits, wind-dispersal)

1. Does not occur (no long-distance dispersal mechanisms) 0
2. Infrequent or inefficient long-distance dispersal (occurs occasionally

despite lack of adaptations) 2

1. Numerous opportunities for long-distance dispersal (species has

adaptations such as pappus, hooked fruit-coats, etc.) 3

1. Unknown

Score:3

Documentation: dispersed by water running over soil and animals-digestion and fur (Parsons and Cuthbertson 1992). It is not clear if local movement can be attributed to small mammals and birds (Oregon 2010).

Identify dispersal mechanisms:

Rationale:

Sources of information:

- 1. Potential to be spread by human activities (both directly and indirectly – possible mechanisms include: commercial sales, use as forage/revegetation, spread along highways, transport on boats, contamination, etc.)

1. Does not occur 0
2. Low (human dispersal is infrequent or inefficient) 1
3. Moderate (human dispersal occurs) 2
4. High (there are numerous opportunities for dispersal to new areas) 3

u. Unknown

Score:3

Documentation: seeds are available for commercial sale for medicinal properties and dye production (Guclu and Ozbek 2007). Can also be dispersed on vehicles and machinery (Parsons and Cuthbertson 1992). Animals can spread seeds in droppings. Agricultural activities can also spread seeds-hay, livestock movement, burning, tilling (Davidson and Wargo)

Identify dispersal mechanisms:

Rationale:

Sources of information:

- 1. Allelopathic

1. no 0
2. yes 2
3. unknown

Score:2

Documentation: contains allelopathic chemicals that act as natural herbicides and reduce growth of surrounding plants giving it a competitive edge (Oregon 2010)

Describe effect on adjacent plants:

Rationale:

Sources of information:

- 1. Competitive ability

a. Poor competitor for limiting factors 0

b. Moderately competitive for limiting factors 1

c. Highly competitive for limiting factors and/or nitrogen fixing ability 3

u. Unknown

Score:3

Documentation: can survive extreme drought and high temperatures as well as exhibiting allelopathy (Abbott et. al. 2008)

Evidence of competitive ability:

Rationale:

Sources of information:

- 1. Forms dense thickets, climbing or smothering growth habit, or otherwise taller than the surrounding vegetation

1. No 0
2. Forms dense thickets 1
3. Has climbing or smothering growth habit, or otherwise taller than the surrounding

vegetation 2

u. Unknown

Score:0

Documentation: can become dominant (Abbott et al 2007). Perennial bushy herb that grows 1 to 1.5 feet tall and 3 to 4 feet in diameter.

Describe growth form:

Rationale:

Sources of information:

- 1. Germination requirements

1. Requires open soil and disturbance to germinate 0
2. Can germinate in vegetated areas but in a narrow range or in special conditions 2
3. Can germinate in existing vegetation in a wide range of conditions 3

u. Unknown

Score:0

Documentation: grows in degraded rangelands and waste areas along roadsides (CFIA 2010)

Describe germination requirements:

Rationale:

Sources of information:

- 1. Other species in the genus invasive in Alberta or elsewhere

1. No 0
2. Yes 3

u. Unknown

Score:0

Documentation:

Species:

Sources of information:

2.9 Aquatic, wetland, or riparian species

a. Not invasive in wetland communities 0

b. Invasive in riparian communities 1

c. Invasive in wetland communities 3

u. Unknown

Score: 1

Documentation: Disturbed areas with moist soil are especially susceptible, such as roadsides, riverbanks or streambanks, riparian corridors, waterways, and drainage or irrigation ditches (USDA 2012)

Describe type of habitat:

Rationale:

Sources of information:

Total Possible:25

Total:15

1. Distribution
   1. Is the species highly domesticated or a weed of agriculture
2. No 0
3. Is occasionally an agricultural pest 2
4. Has been grown deliberately, bred, or is known as a significant agricultural pest 4
5. Unknown

Score:2

Documentation: seeds available commercially for sale and affects rangeland cattle due to non-palatability

Identify reason for selection, or evidence of weedy history:

Rationale:

Sources of information:

- 1. Known level of ecological impact in natural areas

1. Not known to cause impact in any other natural area 0
2. Known to cause impacts in natural areas, but in dissimilar habitats and

climate zones than exist in regions of Alberta 1

1. Known to cause low impact in natural areas in similar habitats and climate

zones to those present in Alberta 3

1. Known to cause moderate impact in natural areas in similar habitat and

climate zones 4

1. Known to cause high impact in natural areas in similar habitat and climate

zones 6

u. Unknown

Score:3

Documentation:

Identify type of habitat and states or provinces where it occurs:

Sources of information:

- 1. Role of anthropogenic and natural disturbance in establishment

1. Requires anthropogenic disturbances to establish 0
2. May occasionally establish in undisturbed areas but can readily establish in

areas with natural disturbances 3

1. Can establish independent of any known natural or anthropogenic disturbances 5

u. Unknown

Score:3

Documentation: Mostly found in disturbed areas

Identify type of disturbance:

Rationale:

Sources of information:

- 1. Current global distribution

1. Occurs in one or two continents or regions (e.g., Mediterranean region) 0
2. Extends over three or more continents 3
3. Extends over three or more continents, including successful introductions in

arctic or subarctic regions 5

u. Unknown

Score:3

Documentation: North Africa, USA, Middle East, central Asia, southeast Europe, Mediterranean, Australia

Describe distribution:

Rationale:

Sources of information:

- 1. Extent of the species Canada range and/or occurrence of formal state or provincial listing

1. 0-5 percent of the states/provinces 0
2. 6-20 percent of the states/provinces 2
3. 21-50 percent, and/or state/province listed as a problem weed

(e.g., “Noxious,” or “Invasive”) in 1 state or Canadian province 4

1. Greater than 50 percent, and/or identified as “Noxious” in 2 or more states or

Canadian provinces 5

u. Unknown

Score:5

Documentation: Noxious weed in Arizona, California, Colorado, New Mexico, Nevada and Oregon. Not regulated in Montana suggesting it has not shown serious invasive characteristics there, even if populations are present. Likely to invade Canada-BC and southern prairies (CFIA 2010)

Identify provinces invaded: absent from Canada

Rationale:

Sources of information:

Total possible:25

Total:16

1. Feasibility of Control
   1. Seed banks
2. Seeds remain viable in the soil for less than 3 years 0
3. Seeds remain viable in the soil for between 3 and 5 years 2
4. Seeds remain viable in the soil for 5 years and more 3

u. Unknown

Score:unknown

Documentation: (Oregon 2010)

Identify longevity of seed bank

Rationale:

Sources of information:

- 1. Vegetative regeneration

1. No resprouting following removal of aboveground growth 0
2. Resprouting from ground-level meristems 1
3. Resprouting from extensive underground system 2
4. Any plant part is a viable propagule 3

u. Unknown

Score:2

Documentation: pieces of rootstock can resprout if cultivated (Parsons and Cuthbertson 1992). It has a deep growing and robust perennial root system (tap root can grow to over 25 feet downwards) that is a major obstacle to plant control (USDA 2012)

Describe vegetative response:

Rationale:

Sources of information:

- 1. Level of effort required

1. Management is not required (e.g., species does not persist without repeated anthropogenic disturbance) 0
2. Management is relatively easy and inexpensive; requires a minor investment in human and financial resources 2
3. Management requires a major short-term investment of human and financial resources, or a moderate long-term investment 3
4. Management requires a major, long-term investment of human and financial resources 4

u. Unknown

Score:4

Documentation: Mechanical removal is a difficult task. The strong, deep taproot and lateral roots must be removed for control. Plants may be cut back to the crown, but regrowth will occur. Tillage will only serve to spread infestations by severing and dragging rootstocks to new areas. Longevity in the seed bank is unknown. Grazing is not an effective control option, due to non-palatability or toxic effects. There are also no available biocontrol agents for this weed. The most effective control strategy is herbicide, applied to the foliage of actively growing plants in the bud stage. It is necessary to repeat this, possibly several times until the plants are completely killed. Nonselective right-of-way herbicide products have provided good roadside control (Oregon 2010).

Identify types of control methods and time-term required:

Rationale:

Sources of information:

Total Possible: 7

Total: 6

Total for 4 sections Possible: 97

Total for 4 sections: 64

References:

Abbott, L.B., Lepar, D. and Daniel, D.L. 2007. Vegetative and reproductive phenology of African rue (Peganum harmala) in the northern Chihuahuan Desert. The Southwestern Naturalist 52(2): 209-218.

Carlson, M. 2008. Invasiveness Ranking System for Non-Native Plants of Alaska. USDA. Available at: http://www.fs.usda.gov/Internet/FSE_DOCUMENTS/fsbdev2_037575.pdf

CFIA 2010. Draft - RMD-10-04: Pest Risk Management Document for *Peganum harmala* (African-rue) in Canada

Davidson J.and Wargo M. Recognition and control of african rue in Nevada Fact Sheet FS-01-45 <http://www.unce.unr.edu/publications/files/nr/2001/FS0145.pdf>

Guclu, C., and Ozbek, H., 2007. African Rue Projects.

Oregon 2010. Oregon Department of Agriculture Plant Pest Risk Assessment for African rue, Peganum harmala 2009 (Revised 2010)

Parsons, W.T., and Cuthbertson, E.G. 1992. Noxious Weeds of Australia. Inkata Press, Melbourne and Sydney, Australia. 692 pp.

USDA 2012. Field Guide for Managing African Rue in the Southwest

Notes

Listed as one of the "worst weeds of the West."

weed of rangelands

prohibited noxious weed under the Weed Seeds Order of the Seeds Act in Canada

extremely drought tolerant-Gobi dessert

the biology and ecology of this species have not been well documented and much of our knowledge about this plant is anecdotal (Abbott et al 2007)

Score Interpretation

While different users will have different concepts of what constitutes various levels of invasiveness (e.g., what is “highly invasive” vs. “moderately invasive” may differ among management agencies), we divided the ranks into six blocks in Appendix A. We consider species with scores ≥80 as “Extremely Invasive” and species with scores 70–79 as “Highly Invasive;” both of these groups are composed of species estimated to be very threaten­ing to Alberta. Species with scores of 60–69 as “Moderately Invasive” and scores of 50–59 represent “Modestly Invasive” species; both of these groups still pose significant risks to ecosystems. Species with scores of 40–49 are “Weakly Invasive”, and <40 are considered “Very Weakly Invasive.” These last two groups generally have not been shown to significantly alter ecosystem processes and communities elsewhere and probably do not require as much attention as the other species.

**Alberta non-native plant invasiveness ranking form**

(Adapted from Carlson et al. 2008)

| Scientific name: | *Sphaerophysa salsula*/*Phaca salsula/Swainsona salsula* |
| --- | --- |
| Common name: | Alkali Swainsonpea/Austrian peaweed |
| Assessor: | Shauna-Lee Chai |
| Reviewers: | David Giblin |
| Date: | October 29, 2013 |

Outcome score:

1. Climatic Comparison

This species is present or may potentially establish in the following natural regions:

|  | Collected in Alberta regions | CLIMEX similarity in 1975 | CLIMEX similarity in 2050 |
| --- | --- | --- | --- |
| Boreal | No | 0.741 | 0.743 |
| Parkland | No | 0.749 | 0.776 |
| Foothills | No | 0.721 | 0.724 |
| Grassland | No | 0.821 | 0.825 |
| Rocky Mountains | No | 0.640 | 0.621 |
| Shield | No | 0.701 | 0.731 |

1. Invasiveness Ranking Total (Total answered^1^ points possible) Total score

| 1. Ecological impact | 40(40) | 28 |
| --- | --- | --- |
| 1. Biological characteristic and dispersal ability | 25(25) | 18 |
| 1. Ecological amplitude and distribution | 25(25) | 19 |
| 1. Feasibility of control | 10(10) | 9 |
| Outcome score | 100(100)^b^ | ^a^74 |
| Relative maximum score^2^ | 74 | *Highly Invasive* |

^1^For questions answered “unknown” do not include point value for the question in parentheses for “Total answered points possible.”

^2^Calculated as a/b x 100.

1. Climatic Comparison:
   1. Has this species ever been collected or documented in Alberta?

__Yes – continue to 1.2

x No – continue to 2.1

1.2 Which natural region has it been collected or documented? Proceed to section B. Invasiveness Ranking.

__Boreal

__Rockies

__Grassland

__Foothills

__Parkland

__Shield

Documentation:

Sources of information: ANPC Rogues gallery, ACIMS, PLANTS database, GBIF

2.1 Is there a 70 percent or higher similarity (based on CLIMEX climate matching) between climates anywhere the species currently occurs and

a. Boreal - Yes

b. Rockies - No

c. Grassland - Yes

d. Foothills - Yes

e. Parkland - Yes

f. Shield - Yes

-If “no” is answered for all regions, reject species from consideration

Documentation:

Sources of information:

1. Invasiveness Ranking
2. Ecological Impact
   1. Impact on Natural Ecosystem Processes
3. No perceivable impact on ecosystem processes 0
4. Has the potential to influence ecosystem processes to a minor degree

(e.g., has a perceivable but mild influence on soil nutrient availability) 3

1. Has the potential to cause significant alteration of ecosystem processes (e.g., increases sedimentation rates along streams or coastlines, reduces open water

that are important to waterfowl) 7

1. May cause major, possibly irreversible, alteration or disruption of ecosystem processes (e.g., the species alters geomorphology; hydrology; or affects fire frequency, altering community composition; species fixes substantial levels of nitrogen in the soil making soil unlikely to support certain native plants or more likely to favor non-native species) 10

u. Unknown

Score:7

Documentation: Can impact nutrient cycling. Invades wetlands, dominates vegetation and reduces food available for game birds, and is unpalatable to wildlife and livestock (Robocker et al. 1964)

Identify ecosystem processes impacted:

Rationale:

Sources of information:

- 1. Impact on Natural Community Structure

1. No perceived impact; establishes in an existing layer without influencing its

structure 0

1. Has the potential to influence structure in one layer (e.g., changes the density

of one layer) 3

1. Has the potential to cause significant impact in at least one layer (e.g., creation

of a new layer or elimination of an existing layer) 7

1. Likely to cause major alteration of structure (e.g., covers canopy, eradicating

most or all layers below) 10

1. Unknown

Score:7

Documentation: Grows to 1.5 m tall in south of range, but about 80 cm in northern range and is aggressive and excludes other vegetation (Robocker et al. 1964)

Identify type of impact or alteration:

Rationale:

Sources of information:

- 1. Impact on Natural Community Composition

1. No perceived impact; causes no apparent change in native populations 0
2. Has the potential to influence community composition (e.g., reduces the

number of individuals in one or more native species in the community) 3

1. Has the potential to significantly alters community composition (e.g., produces

a significant reduction in the population size of one or more native species in

the community) 7

1. Likely to cause major alteration in community composition (e.g., results in the extirpation of one or several native species, reducing biodiversity or change the community composition towards species exotic to the natural community) 10

u. Unknown

Score:7

Documentation: Dominates areas it invades with extremely dense cover (Robocker et al. 1964)

Identify type of impact or alteration:

Rationale:

Sources of information:

- 1. Impact on higher trophic levels (cumulative impact of this species on the animals,

fungi, microbes, and other organisms in the community it invades)

1. Negligible perceived impact 0
2. Has the potential to cause minor alteration 3
3. Has the potential to cause moderate alteration (minor reduction in

nesting/foraging sites, reduction in habitat connectivity, interference with

native pollinators, injurious components such as spines, toxins) 7

1. Likely to cause severe alteration of higher trophic populations (extirpation or endangerment of an existing native species/population, or significant reduction

in nesting or foraging sites) 10

u. Unknown

Score:7

Documentation: unpalatable legume-the value of swainsonpea as a forage plant apparently

is nil. In a grazing test on the McNary Wildlife Refuge in 1959, an area of swainsonpea was fenced and stocked with cattle. The animals refused to eat it, even after other forage was grazed to the ground. In sprinkler and sub-irrigated fields where crops are grown for bird feed, swainsonpea sharply reduces the production of grain and interferes with the feeding of game birds (Robocker et al. 1964).

Identify type of impact or alteration:

Rationale:

Sources of information:

Total Possible:40

Total:28

1. Biological Characteristics and Dispersal Ability
   1. Mode of reproduction
2. Not aggressive reproduction (few [0-10] seeds per plant and no

vegetative reproduction) 0

1. Somewhat aggressive (reproduces only by seeds (11-1,000/m2) 1
2. Moderately aggressive (reproduces vegetatively and/or by a moderate

amount of seed, <1,000/m2) 2

1. Highly aggressive reproduction (extensive vegetative spread and/or

many seeded, >1,000/m2) 3

u. Unknown

Score:3

Documentation: Reproduction by aggressive creeping roots and seed (CDFA, Robocker et al. 1964).

Describe key reproductive characteristics (including seeds per plant):

Rationale:

Sources of information:

- 1. Innate potential for long-distance dispersal (bird dispersal, sticks to animal hair, buoyant fruits, wind-dispersal)

1. Does not occur (no long-distance dispersal mechanisms) 0
2. Infrequent or inefficient long-distance dispersal (occurs occasionally

despite lack of adaptations) 2

1. Numerous opportunities for long-distance dispersal (species has

adaptations such as pappus, hooked fruit-coats, etc.) 3

1. Unknown

Score: 2

Documentation: pers. comm. (Gablin 2014)

Identify dispersal mechanisms:

Rationale:

Sources of information:

- 1. Potential to be spread by human activities (both directly and indirectly – possible mechanisms include: commercial sales, use as forage/revegetation, spread along highways, transport on boats, contamination, etc.)

1. Does not occur 0
2. Low (human dispersal is infrequent or inefficient) 1
3. Moderate (human dispersal occurs) 2
4. High (there are numerous opportunities for dispersal to new areas) 3

u. Unknown

Score:3

Documentation: grows with alfalfa, where alfalfa is grown for seed (CDFA)

Identify dispersal mechanisms:

Rationale:

Sources of information:

- 1. Allelopathic

1. no 0
2. yes 2
3. unknown

Score:0

Documentation:

Describe effect on adjacent plants:

Rationale:

Sources of information:

- 1. Competitive ability

a. Poor competitor for limiting factors 0

b. Moderately competitive for limiting factors 1

c. Highly competitive for limiting factors and/or nitrogen fixing ability 3

u. Unknown

Score:3

Documentation: Highly competitive and fixes nitrogen (Nevada, CDFA)

Evidence of competitive ability:

Rationale:

Sources of information:

- 1. Forms dense thickets, climbing or smothering growth habit, or otherwise taller than the surrounding vegetation

1. No 0
2. Forms dense thickets 1
3. Has climbing or smothering growth habit, or otherwise taller than the surrounding

vegetation 2

u. Unknown

Score:2

Documentation: Grows to 1.5 m tall, has extremely dense cover (Robocker et al. 1964)

Describe growth form:

Rationale:

Sources of information:

- 1. Germination requirements

1. Requires open soil and disturbance to germinate 0
2. Can germinate in vegetated areas but in a narrow range or in special conditions 2
3. Can germinate in existing vegetation in a wide range of conditions 3

u. Unknown

Score:2

Documentation: Grows in disturbed, moist areas-narrow range of germination (Gablin pers. comm.). Average germination of scarified seed was 84 per cent at the end of 98 days. Varying conditions of light and temperature caused no significant difference in germination (Robocker et al. 1964).

Describe germination requirements:

Rationale:

Sources of information:

- 1. Other species in the genus invasive in Alberta or elsewhere

1. No 0
2. Yes 3

u. Unknown

Score:0

Documentation: *Sphaerophysa kotschyana*-not invasive

Species:

Sources of information:

2.9 Aquatic, wetland, or riparian species

a. Not invasive in wetland communities 0

b. Invasive in riparian communities 1

c. Invasive in wetland communities 3

u. Unknown

Score:3

Documentation: High potential for establishment along stream banks, irrigation canals, wasteways, pastures, and meadows with high water tables and other poorly drained areas (Robocker et al. 1964)

Describe type of habitat: S. salsula is a perennial herb which grows in disturbed sites, along roadsides, irrigation ditches and cultivation crops. It thrives in saline soils (Robocker et al. 1964) .

Rationale:

Sources of information:

Total Possible:25

Total:18

1. Distribution
   1. Is the species highly domesticated or a weed of agriculture
2. No 0
3. Is occasionally an agricultural pest 2
4. Has been grown deliberately, bred, or is known as a significant agricultural pest 4
5. Unknown

Score:4

Documentation:Introduced for soil stabilisation and threatens alfalfa seed producers. It is commonly seen in areas where alfalfa is grown, because the seeds of the two species look similar and the weed seed is easily imported with the crop seed (CDFA). In sprinkler and sub-irrigated fields where crops are grown for bird feed, swainsonpea sharply reduces the production of grain and interferes with the feeding of game birds (Robocker et al. 1964).

Identify reason for selection, or evidence of weedy history:

Rationale:

Sources of information:

- 1. Known level of ecological impact in natural areas

1. Not known to cause impact in any other natural area 0
2. Known to cause impacts in natural areas, but in dissimilar habitats and

climate zones than exist in regions of Alberta 1

1. Known to cause low impact in natural areas in similar habitats and climate

zones to those present in Alberta 3

1. Known to cause moderate impact in natural areas in similar habitat and

climate zones 4

1. Known to cause high impact in natural areas in similar habitat and climate

zones 6

u. Unknown

Score:4

Documentation: Example, in the McNary Wildlife Refuge at Burbank, Washington, much of the margin of an oxbow lake is infested with a dense growth of swainsonpea. The infestation extends from the shore line for a considerable distance into relatively barren sand (Robocker et al. 1964).

Identify type of habitat and states or provinces where it occurs: Disturbed sites, roadsides, irrigation ditches, cultivated crops (CDFA)

Sources of information:

- 1. Role of anthropogenic and natural disturbance in establishment

1. Requires anthropogenic disturbances to establish 0
2. May occasionally establish in undisturbed areas but can readily establish in

areas with natural disturbances 3

1. Can establish independent of any known natural or anthropogenic disturbances 5

u. Unknown

Score:3

Documentation:

Identify type of disturbance:

Rationale:

Sources of information:

- 1. Current global distribution

1. Occurs in one or two continents or regions (e.g., Mediterranean region) 0
2. Extends over three or more continents 3
3. Extends over three or more continents, including successful introductions in

arctic or subarctic regions 5

u. Unknown

Score:3

Documentation: Asia, Europe, USA

Describe distribution:

Rationale:

Sources of information:

- 1. Extent of the species Canada range and/or occurrence of formal state or provincial listing

1. 0-5 percent of the states/provinces 0
2. 6-20 percent of the states/provinces 2
3. 21-50 percent, and/or state/province listed as a problem weed

(e.g., “Noxious,” or “Invasive”) in 1 state or Canadian province 4

1. Greater than 50 percent, and/or identified as “Noxious” in 2 or more states or

Canadian provinces 5

u. Unknown

Score:5

Documentation: Noxious in California, Nevada, Oregon, Washington (USDA Plants)

Identify provinces invaded:

Rationale:

Sources of information:

Total possible:25

Total:19

1. Feasibility of Control
   1. Seed banks
2. Seeds remain viable in the soil for less than 3 years 0
3. Seeds remain viable in the soil for between 3 and 5 years 2
4. Seeds remain viable in the soil for 5 years and more 3

u. Unknown

Score:3

Documentation: Like many other legumes, the seeds are extremely hard and may be viable in the soil for many years (CDFA)

Identify longevity of seed bank

Rationale:

Sources of information:

- 1. Vegetative regeneration

1. No resprouting following removal of aboveground growth 0
2. Resprouting from ground-level meristems 1
3. Resprouting from extensive underground system 2
4. Any plant part is a viable propagule 3

u. Unknown

Score:2

Documentation: resprouts from roots (CDFA)

Describe vegetative response:

Rationale:

Sources of information:

- 1. Level of effort required

1. Management is not required (e.g., species does not persist without repeated anthropogenic disturbance) 0
2. Management is relatively easy and inexpensive; requires a minor investment in human and financial resources 2
3. Management requires a major short-term investment of human and financial resources, or a moderate long-term investment 3
4. Management requires a major, long-term investment of human and financial resources 4

u. Unknown

Score:4

Documentation: There is little information regarding control of swainsonpea. Tillage may be ineffective due to an extensive creeping root system that sends up numerous shoots, and may spread severed rootstocks to new areas. Tillage equipment should be thoroughly cleaned after working in infested areas.

Mowing or grazing may reduce seed production, but will be ineffective for complete control. Cattle likely prefer the seed pods of swainsonpea, and seed viability probably remains high after passing through animals. Therefore, cattle should be removed from areas after seed production. Like many other legumes, the seeds are extremely hard and may be viable in the soil for many years.

There is no information regarding chemical control of swainsonpea. Herbicides such as glyphosate, clopyralid, triclopyr, dicamba, 2,4-D, and picloram may be effective in noncrop areas. Control in alfalfa may be difficult, but glyphosate applied with a rope-wick applicator may be effective. However, regrowth is likely with any of these treatments and reapplication may be necessary. Optimal time for treatment is unknown, but other perennial weeds may be most susceptible either at early bloom or during the fall when translocation of carbohydrates to the roots is maximized (CDFA).

Identify types of control methods and time-term required:

Rationale:

Sources of information:

Total Possible: 10

Total:9

Total for 4 sections Possible:100

Total for 4 sections: 74

References:

Carlson, M. 2008. Invasiveness Ranking System for Non-Native Plants of Alaska. USDA. Available at: http://www.fs.usda.gov/Internet/FSE_DOCUMENTS/fsbdev2_037575.pdf

CDFA (California Dept of Food and Agriculture)

<http://www.cdfa.ca.gov/plant/ipc/weedinfo/sphaerophysa-salsula.htm>

Consortium of Pacific Northwest Herbaria database:

http://www.pnwherbaria.org.

DiTomaso, J.M., G.B. Kyser et al. 2013. Weed Control in Natural Areas in the Western United States. Weed Research and Information Center, University of California. 544 pp. (http://wric.ucdavis.edu/information/natural%20areas/wr_S/Sphaerophysa.pdf).

Robocker, A.W.C., Kerr, H. and Bruns, V. 1964. Characteristics and Control of Swainsonpea. Weed Science Society of America, 12: 189–191.

USDA PLANTS database

Nevada <http://agri.nv.gov/Noxious_Weed-Swainsonpea/>

Notes

legume

Often infests roadsides, fencerows and irrigation ditches, stabilised dune areas with high water table.

Perennial; reproduces by seed and creeping roots

Potential contaminant of alfalfa seed due to similar size, shape and weight

eradicated from California

Score Interpretation

While different users will have different concepts of what constitutes various levels of invasiveness (e.g., what is “highly invasive” vs. “moderately invasive” may differ among management agencies), we divided the ranks into six blocks in Appendix A. We consider species with scores ≥80 as “Extremely Invasive” and species with scores 70–79 as “Highly Invasive;” both of these groups are composed of species estimated to be very threaten­ing to Alberta. Species with scores of 60–69 as “Moderately Invasive” and scores of 50–59 represent “Modestly Invasive” species; both of these groups still pose significant risks to ecosystems. Species with scores of 40–49 are “Weakly Invasive”, and <40 are considered “Very Weakly Invasive.” These last two groups generally have not been shown to significantly alter ecosystem processes and communities elsewhere and probably do not require as much attention as the other species.

**Alberta non-native plant invasiveness ranking form**

(Adapted from Carlson et al. 2008)

| Scientific name: | *Elaeagnus umbellata* |
| --- | --- |
| Common name: | Autumn Olive |
| Assessor: | Shauna-Lee Chai |
| Reviewers: | Peter Rice |
| Date: | November 6, 2013 |

Outcome score:

1. Climatic Comparison

This species is present or may potentially establish in the following natural regions:

|  | Collected in Alberta regions | CLIMEX similarity in 1975 | CLIMEX similarity in 2050 |
| --- | --- | --- | --- |
| Boreal | No | 0.674 | 0.729 |
| Parkland | No | 0.746 | 0.789 |
| Foothills | No | 0.757 | 0.786 |
| Grassland | No | 0.740 | 0.761 |
| Rocky Mountains | No | 0.762 | 0.763 |
| Shield | No | 0.590 | 0.661 |

1. Invasiveness Ranking Total (Total answered^1^ points possible) Total score

| 1. Ecological impact | 40(40) | 37 |
| --- | --- | --- |
| 1. Biological characteristic and dispersal ability | 25(25) | 20 |
| 1. Ecological amplitude and distribution | 25(25) | 16 |
| 1. Feasibility of control | 10(7) | 6 |
| Outcome score | 100(97)^b^ | ^a^79 |
| Relative maximum score^2^ | 81 | *Extremely Invasive* |

^1^For questions answered “unknown” do not include point value for the question in parentheses for “Total answered points possible.”

^2^Calculated as a/b x 100.

1. Climatic Comparison:
   1. Has this species ever been collected or documented in Alberta?

__Yes – continue to 1.2

x No – continue to 2.1

1.2 Which natural region has it been collected or documented? Proceed to section B. Invasiveness Ranking.

__Boreal

__Rockies

__Grassland

__Foothills

__Parkland

__Shield

Documentation:

Sources of information: ANPC Rogues gallery, ACIMS, PLANTS database, GBIF

2.1 Is there a 70 percent or higher similarity (based on CLIMEX climate matching) between climates anywhere the species currently occurs and

a. Boreal – Not in 1975, but in 2050

b. Rockies - Yes

c. Grassland - Yes

d. Foothills - Yes

e. Parkland -Yes

f. Shield -No

-If “no” is answered for all regions, reject species from consideration

Documentation:

Sources of information:

1. Invasiveness Ranking
2. Ecological Impact
   1. Impact on Natural Ecosystem Processes
3. No perceivable impact on ecosystem processes 0
4. Has the potential to influence ecosystem processes to a minor degree

(e.g., has a perceivable but mild influence on soil nutrient availability) 3

1. Has the potential to cause significant alteration of ecosystem processes (e.g., increases sedimentation rates along streams or coastlines, reduces open water

that are important to waterfowl) 7

1. May cause major, possibly irreversible, alteration or disruption of ecosystem processes (e.g., the species alters geomorphology; hydrology; or affects fire frequency, altering community composition; species fixes substantial levels of nitrogen in the soil making soil unlikely to support certain native plants or more likely to favor non-native species) 10

u. Unknown

Score:10

Documentation: Nitrogen-fixing capabilities of *E. umbellata* has the capacity to adversely affect the nitrogen cycle of native communities that may depend on infertile soils (Eckardt and Sather, 1987)

Identify ecosystem processes impacted:

Rationale:

Sources of information:

- 1. Impact on Natural Community Structure

1. No perceived impact; establishes in an existing layer without influencing its

structure 0

1. Has the potential to influence structure in one layer (e.g., changes the density

of one layer) 3

1. Has the potential to cause significant impact in at least one layer (e.g., creation

of a new layer or elimination of an existing layer) 7

1. Likely to cause major alteration of structure (e.g., covers canopy, eradicating

most or all layers below) 10

1. Unknown

Score:10

Documentation: can reach high densities in forest understorey being the only species present in the understorey (Edgin and Ebinger 2001)

Identify type of impact or alteration:

Rationale:

Sources of information:

- 1. Impact on Natural Community Composition

1. No perceived impact; causes no apparent change in native populations 0
2. Has the potential to influence community composition (e.g., reduces the

number of individuals in one or more native species in the community) 3

1. Has the potential to significantly alters community composition (e.g., produces

a significant reduction in the population size of one or more native species in

the community) 7

1. Likely to cause major alteration in community composition (e.g., results in the extirpation of one or several native species, reducing biodiversity or change the community composition towards species exotic to the natural community) 10

u. Unknown

Score: 10

Documentation: displaces native plants and can form dense thickets (Munger 2003, Eckardt and Sather (1987)

Identify type of impact or alteration:

Rationale:

Sources of information:

- 1. Impact on higher trophic levels (cumulative impact of this species on the animals,

fungi, microbes, and other organisms in the community it invades)

1. Negligible perceived impact 0
2. Has the potential to cause minor alteration 3
3. Has the potential to cause moderate alteration (minor reduction in

nesting/foraging sites, reduction in habitat connectivity, interference with

native pollinators, injurious components such as spines, toxins) 7

1. Likely to cause severe alteration of higher trophic populations (extirpation or endangerment of an existing native species/population, or significant reduction

in nesting or foraging sites) 10

u. Unknown

Score: 7

Documentation: invades prairies after fire and has ability to reduce prairie habitat (Eckardt and Sather 1987)

Identify type of impact or alteration:

Rationale:

Sources of information:

Total Possible:40

Total:37

1. Biological Characteristics and Dispersal Ability
   1. Mode of reproduction
2. Not aggressive reproduction (few [0-10] seeds per plant and no

vegetative reproduction) 0

1. Somewhat aggressive (reproduces only by seeds (11-1,000/m2) 1
2. Moderately aggressive (reproduces vegetatively and/or by a moderate

amount of seed, <1,000/m2) 2

1. Highly aggressive reproduction (extensive vegetative spread and/or

many seeded, >1,000/m2) 3

u. Unknown

Score:3

Documentation: 66,000 seeds per year can be produced by a plant (USDA 1987)

Describe key reproductive characteristics (including seeds per plant):

Rationale:

Sources of information:

- 1. Innate potential for long-distance dispersal (bird dispersal, sticks to animal hair, buoyant fruits, wind-dispersal)

1. Does not occur (no long-distance dispersal mechanisms) 0
2. Infrequent or inefficient long-distance dispersal (occurs occasionally

despite lack of adaptations) 2

1. Numerous opportunities for long-distance dispersal (species has

adaptations such as pappus, hooked fruit-coats, etc.) 3

1. Unknown

Score:3

Documentation: Birds use it as a food source (USDA)

Identify dispersal mechanisms:

Rationale:

Sources of information:

- 1. Potential to be spread by human activities (both directly and indirectly – possible mechanisms include: commercial sales, use as forage/revegetation, spread along highways, transport on boats, contamination, etc.)

1. Does not occur 0
2. Low (human dispersal is infrequent or inefficient) 1
3. Moderate (human dispersal occurs) 2
4. High (there are numerous opportunities for dispersal to new areas) 3

u. Unknown

Score:3

Documentation: It was planted and has escaped. Autumn-olive has been promoted for reclamation of mine spoils and other disturbed soils and is an ornamental. It has been planted for reclamation of surface coal mine sites because it is tolerant of low pH soil conditions often found on these sites. It has also been suggested for use in stabilizing eroded soils in exposed coastal areas due to its salt spray tolerance. An additional benefit to planting autumn-olive in these and other situations, where reclamation of disturbed and frequently nutrient-poor soils is an important objective, is its ability to fix atmospheric nitrogen. Autumn-olive has been a recommended species for planting as a tall shrub component in windbreaks in the Great Plains, in part due to its wildlife food and cover value.

Identify dispersal mechanisms:

Rationale:

Sources of information:

- 1. Allelopathic

1. no 0
2. yes 2
3. unknown

Score:0

Documentation: USDA PLANTS

Describe effect on adjacent plants:

Rationale:

Sources of information:

- 1. Competitive ability

a. Poor competitor for limiting factors 0

b. Moderately competitive for limiting factors 1

c. Highly competitive for limiting factors and/or nitrogen fixing ability 3

u. Unknown

Score: 3

Documentation: High drought tolerance, able to fix nitrogen, rapid growth rate (USDA PLANTS)

Evidence of competitive ability:

Rationale:

Sources of information:

- 1. Forms dense thickets, climbing or smothering growth habit, or otherwise taller than the surrounding vegetation

1. No 0
2. Forms dense thickets 1
3. Has climbing or smothering growth habit, or otherwise taller than the surrounding

vegetation 2

u. Unknown

Score:2

Documentation: Autumn-olive is a many-branched, deciduous shrub or shrubby tree, growing 10 to16 feet (3-5 m) tall

Describe growth form:

Rationale:

Sources of information:

- 1. Germination requirements

1. Requires open soil and disturbance to germinate 0
2. Can germinate in vegetated areas but in a narrow range or in special conditions 2
3. Can germinate in existing vegetation in a wide range of conditions 3

u. Unknown

Score:2

Documentation: Grows well on variety of soils, is moderately shade tolerant, but is thought to be generally absent from areas with very low light intensity, such as under a dense forest canopy (Nestleroad and Zimmerman 1987). It has been recorded under canopy however in Illinois (Ebinger and Lehnen 1981). Primarily a ruderale and not particularly invasive of undisturbed vegetated habitats even under favorable climatic conditions (Munger 2003).

Describe germination requirements:

Rationale:

Sources of information:

- 1. Other species in the genus invasive in Alberta or elsewhere

1. No 0
2. Yes 3

u. Unknown

Score:3

Documentation: Invasive Russian Olive (*Elaeagnus angustifolia*) sold as an ornamental in Alberta

Species:

Sources of information:

2.9 Aquatic, wetland, or riparian species

a. Not invasive in wetland communities 0

b. Invasive in riparian communities 1

c. Invasive in wetland communities 3

u. Unknown

Score:1

Documentation: does not grow well on very wet sites (Eckardt and Sather, 1987). Found on riparian rangeland (Munger 2003)

Describe type of habitat:

Rationale:

Sources of information:

Total Possible:25

Total:20

1. Distribution
   1. Is the species highly domesticated or a weed of agriculture?
2. No 0
3. Is occasionally an agricultural pest 2
4. Has been grown deliberately, bred, or is known as a significant agricultural pest 4
5. Unknown

Score:4

Documentation: Deliberately grown although not an agricultural weed. Autumn olive was first imported for cultivation into the U.S. in 1830 from Japan (Invasive Plant Atlas of New England - IPANE). It has been promoted as a landscape, reclamation and wildlife food planting ever since.

Identify reason for selection, or evidence of weedy history:

Rationale:

Sources of information:

- 1. Known level of ecological impact in natural areas

1. Not known to cause impact in any other natural area 0
2. Known to cause impacts in natural areas, but in dissimilar habitats and

climate zones than exist in regions of Alberta 1

1. Known to cause low impact in natural areas in similar habitats and climate

zones to those present in Alberta 3

1. Known to cause moderate impact in natural areas in similar habitat and

climate zones 4

1. Known to cause high impact in natural areas in similar habitat and climate

zones 6

u. Unknown

Score:1

Documentation: Problem in natural areas (Nestleroad et al. 1987). Rice pers. comm-score b.

Identify type of habitat and states or provinces where it occurs: forests prairies fields

Sources of information:

- 1. Role of anthropogenic and natural disturbance in establishment

1. Requires anthropogenic disturbances to establish 0
2. May occasionally establish in undisturbed areas but can readily establish in

areas with natural disturbances 3

1. Can establish independent of any known natural or anthropogenic disturbances 5

u. Unknown

Score:3

Documentation:

Identify type of disturbance:

Rationale:

Sources of information:

- 1. Current global distribution

1. Occurs in one or two continents or regions (e.g., Mediterranean region) 0
2. Extends over three or more continents 3
3. Extends over three or more continents, including successful introductions in

arctic or subarctic regions 5

u. Unknown

Score:3

Documentation: North America, Asia, Australia

Describe distribution:

Rationale:

Sources of information:

- 1. Extent of the species Canada range and/or occurrence of formal state or provincial listing

1. 0-5 percent of the states/provinces 0
2. 6-20 percent of the states/provinces 2
3. 21-50 percent, and/or state/province listed as a problem weed

(e.g., “Noxious,” or “Invasive”) in 1 state or Canadian province 4

1. Greater than 50 percent, and/or identified as “Noxious” in 2 or more states or

Canadian provinces 5

u. Unknown

Score:5

Documentation: Noxious in Connecticut, Massachusetts, New Hampshire and West Virginia (USDA Plants)

Identify provinces invaded:

Rationale:

Sources of information:

Total possible:25

Total:16

1. Feasibility of Control
   1. Seed banks
2. Seeds remain viable in the soil for less than 3 years 0
3. Seeds remain viable in the soil for between 3 and 5 years 2
4. Seeds remain viable in the soil for 5 years and more 3

u. Unknown

Score:unknown

Documentation: unknown (Munger 2003)

Identify longevity of seed bank

Rationale:

Sources of information:

- 1. Vegetative regeneration

1. No resprouting following removal of aboveground growth 0
2. Resprouting from ground-level meristems 1
3. Resprouting from extensive underground system 2
4. Any plant part is a viable propagule 3

u. Unknown

Score:2

Documentation: vigorous re-sprouting from burned, cut or mowed plants (Szafoni 1991)

Describe vegetative response:

Rationale:

Sources of information:

- 1. Level of effort required

1. Management is not required (e.g., species does not persist without repeated anthropogenic disturbance) 0
2. Management is relatively easy and inexpensive; requires a minor investment in human and financial resources 2
3. Management requires a major short-term investment of human and financial resources, or a moderate long-term investment 3
4. Management requires a major, long-term investment of human and financial resources 4

u. Unknown

Score:4

Documentation: Control requires frequent monitoring and repeated treatments

Identify types of control methods and time-term required: hand pulling seedlings, treating cut surfaces with glyphosate. For larger plants, basal-bark application of triclopyr or 2,4-D (Edgin, Bob; Ebinger, John E. 2001)

Rationale:

Sources of information:

Total Possible:7

Total: 6

Total for 4 sections Possible: 97

Total for 4 sections: 82

References:

Carlson, M. 2008. Invasiveness Ranking System for Non-Native Plants of Alaska. USDA. Available at: <http://www.fs.usda.gov/Internet/FSE_DOCUMENTS/fsbdev2_037575.pdf>

Catling, P. M.; Oldham, M. J.; Sutherland, D. A.; Brownell, V. R.; Larson, B. M. H. 1997. The recent spread of autumn-olive, Elaeagnus umbellata, into southern Ontario and its current status. Canadian Field Naturalist. 111(3): 376-380.

Ebinger, John; Lehnen, Larry. 1981. Naturalized autumn olive in Illinois. Transactions, Illinois State Academy of Science. 74(3&4): 83-85.

Eckhardt, N. and Sather, N. (1987) Element Stewardship Abstract for *Elaeagnus umbellata*

Edgin, Bob; Ebinger, John E. 2001. Control of autumn olive (Elaeagnus umbellata Thunb.) at Beall Woods Nature Preserve, Illinois, USA. Natural Areas Journal. 21(4): 386-388

Henry, Jimmy. 1980. A bonanza for wildlife. Soil Conservation. 45(8): 13.

Munger, Gregory T. 2003. Elaeagnus umbellata. In: Fire Effects Information System, [Online]. U.S. Department of Agriculture, Forest Service, Rocky Mountain Research Station, Fire Sciences Laboratory (Producer). Available: http://www.fs.fed.us/database/feis/ [2013, November 6].

Nestleroad, James; Zimmerman, Douglas; Ebinger, John. 1987. Autumn olive reproduction in three Illinois state parks. Transactions, Illinois Academy of Science. 80(1&2): 33-39.

Szafoni, Robert E. 1991. Vegetation management guideline: autumn olive, Elaeagnus umbellata Thunb. Natural Areas Journal. 11(2): 121-122.

USDA PLANTS database

U.S. Department of Agriculture, Soil Conservation Service. 1987. `Redwing' autumn olive. Program Aid Number 1392. Washington, DC. 4 p

Notes

A serious threat to native communities across many parts of the Eastern USA. Top invasive plants in natural habitats of Canada in 2005- <http://www.ou.edu/cas/botany-micro/ben/ben345.html>

Peter Rice pers. comm.-Autumn olive was first imported for cultivation into the U.S. in 1830 from Japan Invasive Plant Atlas of New England - IPANE). It has been promoted as a landscape, reclamation and wildlife food planting ever since. Autumn olive seeds are dispersed by birds into natural and semi-natural habitats. These efficient dispersal mechanisms, particularly birds, are what have allowed the plant to spread throughout New England (IPANE). However, autumn olive is currently not reported as prevalent in the interior west by EDDMaps. The one occurrence record for Montana (from 1953) in the INVADERS Database System (http://invader.dbs.umt.edu) was in a university town (Bozeman) nursery. There have been no additional reports of autumn olive in Montana although the state has a strong and active new invaders and EDRR program. A new (Lesica 2012) Manual of Montana Vascular Plants does not recognize autumn olive as present in Montana. The single Idaho record (from 2006) in INVADERS is in a university urban (Moscow) riparian area. The INVADERS record for Idaho not suggest a significant infestation.

Score Interpretation

While different users will have different concepts of what constitutes various levels of invasiveness (e.g., what is “highly invasive” vs. “moderately invasive” may differ among management agencies), we divided the ranks into six blocks in Appendix A. We consider species with scores ≥80 as “Extremely Invasive” and species with scores 70–79 as “Highly Invasive;” both of these groups are composed of species estimated to be very threaten­ing to Alberta. Species with scores of 60–69 as “Moderately Invasive” and scores of 50–59 represent “Modestly Invasive” species; both of these groups still pose significant risks to ecosystems. Species with scores of 40–49 are “Weakly Invasive”, and <40 are considered “Very Weakly Invasive.” These last two groups generally have not been shown to significantly alter ecosystem processes and communities elsewhere and probably do not require as much attention as the other species.

**Alberta non-native plant invasiveness ranking form**

(Adapted from Carlson et al. 2008)

| Scientific name: | *Cynanchum louiseae/* *Vincetoxicum nigrum* |
| --- | --- |
| Common name: | Black Swallow-wort |
| Assessor: | Shauna-Lee Chai |
| Reviewers: | Lindsey Milbrath |
| Date: | October 24, 2013 |

Outcome score:

1. Climatic Comparison

This species is present or may potentially establish in the following natural regions:

|  | Collected in Alberta regions | CLIMEX similarity in  1975 | CLIMEX similarity in 2050 |
| --- | --- | --- | --- |
| Boreal | No | 0.673 | 0.728 |
| Parkland | No | 0.746 | 0.789 |
| Foothills | No | 0.722 | 0.759 |
| Grassland | No | 0.740 | 0.761 |
| Rocky Mountains | No | 0.664 | 0.703 |
| Shield | No | 0.590 | 0.661 |

1. Invasiveness Ranking Total (Total answered^1^ points possible) Total score

| 1. Ecological impact | 40(40) | 28 |
| --- | --- | --- |
| 1. Biological characteristic and dispersal ability | 25(25) | 20 |
| 1. Ecological amplitude and distribution | 25(25) | 13 |
| 1. Feasibility of control | 10(7) | 5 |
| Outcome score | 100(97)^b^ | ^a^66 |
| Relative maximum score^2^ | 68 | *Moderately Invasive* |

^1^For questions answered “unknown” do not include point value for the question in parentheses for “Total answered points possible.”

^2^Calculated as a/b x 100.

1. Climatic Comparison:
   1. Has this species ever been collected or documented in Alberta?

__Yes – continue to 1.2

x No – continue to 2.1

Which natural region has it been collected or documented (see inset map)? Proceed to section B. Invasiveness Ranking.

__Boreal

__Rockies

__Grassland

__Foothills

__Parkland

__Shield

Documentation:

Sources of information: ANPC Rogues gallery, ACIMS, PLANTS database, GBIF

2.1 Is there a 70 percent or higher similarity (based on CLIMEX climate matching) between climates anywhere the species currently occurs and

a. Boreal – Not in 1975, but in 2050

b. Rockies - Not in 1975, but in 2050

c. Grassland - Yes

d. Foothills - Yes

e. Parkland - Yes

f. Shield - No

-If “no” is answered for all regions, reject species from consideration

Documentation:

Sources of information:

1. Invasiveness Ranking
2. Ecological Impact
   1. Impact on Natural Ecosystem Processes
3. No perceivable impact on ecosystem processes 0
4. Has the potential to influence ecosystem processes to a minor degree

(e.g., has a perceivable but mild influence on soil nutrient availability) 3

1. Has the potential to cause significant alteration of ecosystem processes (e.g., increases sedimentation rates along streams or coastlines, reduces open water

that are important to waterfowl) 7

1. May cause major, possibly irreversible, alteration or disruption of ecosystem processes (e.g., the species alters geomorphology; hydrology; or affects fire frequency, altering community composition; species fixes substantial levels of nitrogen in the soil making soil unlikely to support certain native plants or more likely to favor non-native species) 10

u. Unknown

Score:7

Documentation: Ecosystem effects have not yet been studied. The species (when densities are high) causes ecosystem-wide reduction in light availability for co-occurring species (Lawlor 2006).

Identify ecosystem processes impacted:

Rationale:

Sources of information:

- 1. Impact on Natural Community Structure

1. No perceived impact; establishes in an existing layer without influencing its

structure 0

1. Has the potential to influence structure in one layer (e.g., changes the density

of one layer) 3

1. Has the potential to cause significant impact in at least one layer (e.g., creation

of a new layer or elimination of an existing layer) 7

1. Likely to cause major alteration of structure (e.g., covers canopy, eradicating

most or all layers below) 10

1. Unknown

Score:7

Documentation: climbing habit means it is a strong light competitor reducing light availability for other forbs, shrubs and saplings. It can overtop and smother shrubs forming the dominant cover. It can comprise the dominant cover in the herbaceous understory layer (Lawlor, 2006, Ditommaso et al. 2005). We have essentially no empirical data to date to confirm if there are community impacts of black swallow-wort infestations. It is an assumption based on high density stands of the weed (Lawlor pers. comm.).

Identify type of impact or alteration:

Rationale:

Sources of information:

- 1. Impact on Natural Community Composition

1. No perceived impact; causes no apparent change in native populations 0
2. Has the potential to influence community composition (e.g., reduces the

number of individuals in one or more native species in the community) 3

1. Has the potential to significantly alter community composition (e.g., produces

a significant reduction in the population size of one or more native species in

the community) 7

1. Likely to cause major alteration in community composition (e.g., results in the extirpation of one or several native species, reducing biodiversity or change the community composition towards species exotic to the natural community) 10

u. Unknown

Score:7

Documentation: Establishment of C. louiseae is threatening the endemic Jessop’s milkvetch, Astragalus rob- binsii (Oakes) A. Gray at Windsor (VT) on ice-scoured banks of the Connecticut River (Ditommaso et al. 2005). Its ability to form monocultures suggests it alters species composition of communities and competes for moisture, nutrients and light (DiTommaso et al., 2005; Lawlor 2006). We do have unpublished data for pale swallow-wort but correlations between pale swallow-wort densities and plant community richness and cover often do not show a negative effect unless swallow-wort densities are very high. Plus the question is always whether or how much of the correlated reduction is due to swallow-wort or some other factor (land use history, deer pressure; Lawlor pers. comm.).

Identify type of impact or alteration:

Rationale:

Sources of information:

- 1. Impact on higher trophic levels (cumulative impact of this species on the animals,

fungi, microbes, and other organisms in the community it invades)

1. Negligible perceived impact 0
2. Has the potential to cause minor alteration 3
3. Has the potential to cause moderate alteration (minor reduction in

nesting/foraging sites, reduction in habitat connectivity, interference with

native pollinators, injurious components such as spines, toxins) 7

1. Likely to cause severe alteration of higher trophic populations (extirpation or endangerment of an existing native species/population, or significant reduction

in nesting or foraging sites) 10

u. Unknown

Score:7

Documentation: low threat-deleterious effect on monarch butterfly via displaced oviposition and larval mortality and potential reduction of host plant availability through displacement. Also hosts rusts of Pinus spp in Europe. Although some insect and mite pests do utilize black and pale swallow-wort as host plants, their densities are very low (Milbrath 2010). Thus, swallow-worts are not likely to serve as a reservoir for pests. Studies of the related pale swallow-wort suggests a decline in arthropod and grassland bird diversity. Toxic to grazing mammals. (Ditommaso et al. 2005, Lawlor 2006)

Identify type of impact or alteration:

Rationale:

Sources of information:

Total Possible:40

Total:28

1. Biological Characteristics and Dispersal Ability
   1. Mode of reproduction
2. Not aggressive reproduction (few [0-10] seeds per plant and no

vegetative reproduction) 0

1. Somewhat aggressive (reproduces only by seeds (11-1,000/m2) 1
2. Moderately aggressive (reproduces vegetatively and/or by a moderate

amount of seed, <1,000/m2) 2

1. Highly aggressive reproduction (extensive vegetative spread and/or

many seeded, >1,000/m2) 3

u. Unknown

Score:3

Documentation: Reproduces by seed. References to rhizomes (e.g., Lumer and Yost 1995) have not been confirmed, although occasionally a rhizome-like connection can be found between two root crowns of black swallow-wort plants; this has never been seen in pale swallow-wort (LR Milbrath, personal observation). (rhizome propagation has recently been disproved (Cappuccino, 2004)). Vegetative expansion (increasing numbers of stems over time from a root crown) does occur but does not contribute to an increase in plant density, only an increase in stem density (Averill et al. 2011). Seed production per stem is similar between pale and black swallow-wort (Averill et al. 2011). Therefore, it is possible that high density infestations of black swallow-wort could produce >10,000 viable seed/m2 as has been reported for pale swallow-wort (Smith et al. 2006). 2090 seeds per square meter per plant can be produced (Uva et al. 1997).

Describe key reproductive characteristics (including seeds per plant):

Rationale:

Sources of information:

- 1. Innate potential for long-distance dispersal (bird dispersal, sticks to animal hair, buoyant fruits, wind-dispersal)

1. Does not occur (no long-distance dispersal mechanisms) 0
2. Infrequent or inefficient long-distance dispersal (occurs occasionally

despite lack of adaptations) 2

1. Numerous opportunities for long-distance dispersal (species has

adaptations such as pappus, hooked fruit-coats, etc.) 3

1. Unknown

Score:3

Documentation: long distance dispersal by wind facilitated through long hairs and occurs readily (Lawlor 2006)

Identify dispersal mechanisms:

Rationale:

Sources of information:

- 1. Potential to be spread by human activities (both directly and indirectly – possible mechanisms include: commercial sales, use as forage/revegetation, spread along highways, transport on boats, contamination, etc.)

1. Does not occur 0
2. Low (human dispersal is infrequent or inefficient) 1
3. Moderate (human dispersal occurs) 2
4. High (there are numerous opportunities for dispersal to new areas) 3

u. Unknown

Score:2

Documentation:found close to human habitation in gardens. May be spread by harvesting or hay making; may have been used as an ornamental originally but no such use is known now (Ditommaso et al. 2005)

Identify dispersal mechanisms:

Rationale:

Sources of information:

- 1. Allelopathic

1. no 0
2. yes 2
3. unknown

Score:2

Documentation: swallowwort root exudates caused significant root length reductions (e.g., 40%for butterfly milkweed and 20% for large crabgrass) and reduced germination (e.g., 25% for lettuce) of indicator species in laboratory studies (Douglass et al. 2011). Also see Gibson et al. 2011. However, it is not certain if allelopathy occurs in the field (processing of soil samples is currently underway-Milbrath pers. comm.).

Describe effect on adjacent plants:

Rationale:

Sources of information:

- 1. Competitive ability

a. Poor competitor for limiting factors 0

b. Moderately competitive for limiting factors 1

c. Highly competitive for limiting factors and/or nitrogen fixing ability 3

u. Unknown

Score:1

Documentation: Could be competitive for light due to climbing habit (Ditommaso et al. 2005). Blanchard et al. (2010) indicated that pale swallow-wort is not more competitive than some native plants.

Evidence of competitive ability:

Rationale:

Sources of information:

- 1. Forms dense thickets, climbing or smothering growth habit, or otherwise taller than the surrounding vegetation

1. No 0
2. Forms dense thickets 1
3. Has climbing or smothering growth habit, or otherwise taller than the surrounding

vegetation 2

u. Unknown

Score:2

Documentation: forms dense populations and is a climber (Ditommaso et al. 2005)

Describe growth form:

Rationale:

Sources of information:

- 1. Germination requirements

1. Requires open soil and disturbance to germinate 0
2. Can germinate in vegetated areas but in a narrow range or in special conditions 2
3. Can germinate in existing vegetation in a wide range of conditions 3

u. Unknown

Score:3

Documentation: Seems to prefer high light environments, some degree of shade tolerance (but not to the extent of pale swallow-wort), grows in some woodland understoreys (Ditommaso et al 2005). Can establish across a wide range of soil pH (Magidow et al. 2013)

Describe germination requirements:

Rationale:

Sources of information:

- 1. Other species in the genus invasive in Alberta or elsewhere

1. No 0
2. Yes 3

u. Unknown

Score:3

Documentation:

Species: *Cynanchum rossicum* (pale swallow-wort), it is more likely to spread to other areas from where it is planted, more competitive and effective at dispersal (DiTommaso et al. 2005)

Sources of information:

2.9 Aquatic, wetland, or riparian species

a. Not invasive in wetland communities 0

b. Invasive in riparian communities 1

c. Invasive in wetland communities 3

u. Unknown

Score:1

Documentation: Although referred to as an upland species, it can occur in wetlands (Lawlor 2006, Ditommaso et al 2005). Black (and pale) swallow-wort can occur in areas labelled wetland, but they will be in the drier areas because neither species tolerates perennially wet soils (Milbrath pers. comm.).

Describe type of habitat:

Rationale:

Sources of information:

Total Possible:25

Total:20

1. Distribution
   1. Is the species highly domesticated or a weed of agriculture
2. No 0
3. Is occasionally an agricultural pest 2
4. Has been grown deliberately, bred, or is known as a significant agricultural pest 4
5. Unknown

Score:2

Documentation: Noted weed pest in nurseries and pastures. Hosts rusts of Pinus spp in Europe. Although some insect and mite pests do utilize black and pale swallow-wort as host plants, their densities are very low (Milbrath 2010). Thus, swallow-worts are not likely to serve as a reservoir for pests.

Identify reason for selection, or evidence of weedy history:

Rationale:

Sources of information:

- 1. Known level of ecological impact in natural areas

1. Not known to cause impact in any other natural area 0
2. Known to cause impacts in natural areas, but in dissimilar habitats and

climate zones than exist in regions of Alberta 1

1. Known to cause low impact in natural areas in similar habitats and climate

zones to those present in Alberta 3

1. Known to cause moderate impact in natural areas in similar habitat and

climate zones 4

1. Known to cause high impact in natural areas in similar habitat and climate

zones 6

u. Unknown

Score:3

Documentation: E.G. pale swallow wort disturbs unique alvar ecosystems in western New York (Great Lakes basin) (Douglass et al. 2009)-different environment to Alberta however. Lack of documentation, but low impacts present (Milbrath pers. comm.)

Identify type of habitat and states or provinces where it occurs:

Sources of information:

- 1. Role of anthropogenic and natural disturbance in establishment

1. Requires anthropogenic disturbances to establish 0
2. May occasionally establish in undisturbed areas but can readily establish in

areas with natural disturbances 3

1. Can establish independent of any known natural or anthropogenic disturbances 5

u. Unknown

Score:3

Documentation: establishes in upland disturbed and undisturbed areas (Ditommaso et al. 2005, Lawlor 2006)

Identify type of disturbance: woodlands, pastures, old fields, shores, flood plains and ruderal areas

Rationale:

Sources of information:

- 1. Current global distribution

1. Occurs in one or two continents or regions (e.g., Mediterranean region) 0
2. Extends over three or more continents 3
3. Extends over three or more continents, including successful introductions in

arctic or subarctic regions 5

u. Unknown

Score: 0

Documentation: Europe and North America. Cynanchum louiseae appears to be rapidly expanding its range in North America and there is no evidence threat it is near to reaching maximum geographic or ecological distribution; future growth is expected (DiTommaso et al., 2005).

Describe distribution:

Rationale:

Sources of information:

- 1. Extent of the species Canada range and/or occurrence of formal state or provincial listing

1. 0-5 percent of the states/provinces 0
2. 6-20 percent of the states/provinces 2
3. 21-50 percent, and/or state/province listed as a problem weed

(e.g., “Noxious,” or “Invasive”) in 1 state or Canadian province 4

1. Greater than 50 percent, and/or identified as “Noxious” in 2 or more states or

Canadian provinces 5

u. Unknown

Score:5

Documentation: Noxious in Ontario, Connecticut, Massachusetts, New Hampshire and Vermont (USDA PLANTS)

Identify provinces invaded: Ontario, Quebec

Rationale:

Sources of information: Record for BC is for pale swallow-wort only, and is not considered current (Milbrath pers. comm.).

Total possible:25

Total:13

1. Feasibility of Control
   1. Seed banks
2. Seeds remain viable in the soil for less than 3 years 0
3. Seeds remain viable in the soil for between 3 and 5 years 2
4. Seeds remain viable in the soil for 5 years and more 3

u. Unknown

Score:unknown

Documentation: studies not found (Ditommaso et al 2005). For pale swallow-wort, some seed is known to survive up to 3 years (e.g., Averill et al. 2010) but it isn’t clear if we could assume the same for black swallow-wort (Milbrath pers. comm.).

Identify longevity of seed bank

Rationale:

Sources of information:

- 1. Vegetative regeneration

1. No resprouting following removal of aboveground growth 0
2. Resprouting from ground-level meristems 1
3. Resprouting from extensive underground system 2
4. Any plant part is a viable propagule 3

u. Unknown

Score:1

Documentation: re-sprouts however do not assist in population spread (Ditommaso et al. 2005). Readily regrows from axillary buds or crown buds when frosted or cut, but root crown is limited in size (Milbrath, personal observation).

Describe vegetative response:

Rationale:

Sources of information:

- 1. Level of effort required

1. Management is not required (e.g., species does not persist without repeated anthropogenic disturbance) 0
2. Management is relatively easy and inexpensive; requires a minor investment in human and financial resources 2
3. Management requires a major short-term investment of human and financial resources, or a moderate long-term investment 3
4. Management requires a major, long-term investment of human and financial resources 4

u. Unknown

Score:4

Documentation: Lawlor (2006)- There has been some experimentation on the use of herbicides to control pale swallow-wort populations. In general, these studies suggest that chemical control alone is not effective, and long-term reapplication is needed to sustain control. Mowing will not eradicate populations but can be used to prevent a seed crop if done in early to mid-July. Digging up plants may control swallow-wort populations, though it would likely only be practical for small populations or isolated plants. Because broken root crowns left on or in the ground may sprout, care must be taken to remove and dispose of the entire root crown.

Identify types of control methods and time-term required:

Rationale:

Sources of information:

Total Possible:7

Total: 5

Total for 4 sections Possible:97

Total for 4 sections:74

References:

Averill, K.M., A. DiTommaso, C.L. Mohler, and L.R. Milbrath. 2010. Establishment of the invasive perennial *Vincetoxicum rossicum* across a disturbance gradient in New York State, USA. Plant Ecol. 211:65–77.

Averill, K.M., A. DiTommaso, C.L. Mohler, and L.R. Milbrath. 2011. Survival, growth, and fecundity of the invasive swallowworts (*Vincetoxicum rossicum* and *V. nigrum*) in New York State. Invasive Plant Science and Management 4: 198-206.

Blanchard, M.L., J.N. Barney, K.M. Averill, C.L. Mohler, and A. DiTommaso. 2010. Does polyembryony confer a competitive advantage to the invasive perennial vine *Vincetoxicum rossicum* (Apocynaceae)? American Journal of Botany 97(2): 251–260.

Carlson, M. 2008. Invasiveness Ranking System for Non-Native Plants of Alaska. USDA. Available at: http://www.fs.usda.gov/Internet/FSE_DOCUMENTS/fsbdev2_037575.pdf

Cappuccino, N. 2004. Allee effect in an invasive alien plant, pale swallowwort Vincetoxicum rossicum (Asclepiadaceae). Oikos 106:3–8.

Ditommaso, A., Lawlor, F.M. & Darbyshire, S.J., 2005. The Biology of Invasive Alien Plants in Canada . 2 . Cynanchum rossicum ( Kleopow ) Borhidi [ = Vincetoxicum rossicum ( Kleopow ) Barbar .] and Cynanchum louiseae ( L .) Kartesz & Gandhi [ = Vincetoxicum nigrum ( L .) Moench ]. , (Forster 1991).

Douglass, C.H., Weston, L. a. & Wolfe, D., 2011. Phytotoxicity and Potential Allelopathy in Pale (Cynanchum rossicum) and Black swallowwort (C. nigrum). Invasive Plant Science and Management, 4(1), pp.133–141. Available at: http://www.bioone.org/doi/abs/10.1614/IPSM-D-10-00021.1 [Accessed January 2, 2014].

Douglass, C. H., L. A. Weston, and A. DiTommaso. 2009. Black and pale swallowwort (Vincetoxicum nigrum sand V. Rossicum): the biology and ecology of two perennial, exotic and invasive vines. Pages 261– 276 in Inderjit, ed. Management of Invasive Weeds. New York: Springer Science + Business Media B.V

Gibson, D.M., S.B. Krasnoff, J. Biazzo, and L. Milbrath. 2011. Phytotoxicity of antofine from invasive swallow-worts. Journal of Chemical Ecology 37: 871-879.

Lawlor, F. 2006. Fact sheet: Black swallow-wort (Cynanchum louiseae Kartesz & Gandhi), [Online]. In: Weeds gone wild: Alien plant invaders of natural areas. Plant Conservation Alliance, Alien Plant Working Group (Producer). Available: http://www.nps.gov/plants/alien/fact/cylo1.htm

Lumer, C., and S.E. Yost. 1995. The reproductive biology of *Vincetoxicum nigrum* (L.) Moench (Asclepiadaceae), a Mediterranean weed in New York State. Bull. Torrey Bot. Club 122: 15-23.

Magidow, L.C., DiTommaso, A., Ketterings, Q.M., Mohler, C.L., and Milbrath, L.R. 2013. Emergence and performance of two invasive swallowworts (*Vincetoxicum* spp.) in contrasting soil types and soil pH. Invasive Plant Sci Manag 6: 281-291.

Milbrath, L.R. 2010. Phytophagous arthropods of invasive swallow-wort vines (*Vincetoxicum* spp.) in New York. Environmental Entomology 39: 68-78.

Mogg, C, Petit, P, Cappuccino, N, Durst, T, McKague, C, Foster, M, Yack, JE, Arnason, JT, Smith, ML. 2008. Tests of the antibiotic properties of the invasive vine *Vincetoxicum rossicum* against bacteria, fungi and insects. Biochem Syst Ecol 36:383-391

Smith, L.L., A. DiTommaso, J. Lehmann, and S. Greipsson. 2006. Growth and reproductive potential of the invasive exotic vine *Vincetoxicum rossicum* in Northern New York State. Can. J. Bot. 84: 1771-1780.

Uva, R.H., J.C. Neal, and J.M. DiTomaso. 1997. Weeds of the Northeast. Cornell University Press: Ithaca, New York. 397 pp.

Notes:

Natureserve I-rank: High

<http://www.natureserve.org/explorer/servlet/NatureServe?sourceTemplate=tabular_report.wmt&loadTemplate=species_RptComprehensive.wmt&selectedReport=RptComprehensive.wmt&summaryView=tabular_report.wmt&elKey=151919&paging=home&save=true&startIndex=1&nextStartIndex=1&reset=false&offPageSelectedElKey=151919&offPageSelectedElType=species&offPageYesNo=true&post_processes=&radiobutton=radiobutton&selectedIndexes=151919>

milkweed, vine, both shade and light tolerant, grows in woodland understoreys

Native Range: This species is native to western European Mediterranean regions (Lawlor, 2006). Tewksbury et al. (2002) list native distribution as southwestern Europe in France, Italy, Portugal, and Spain.

Most of the literature, is biased toward pale swallow-wort (V. rossicum), so it is basically assumed that known effects apply to black swallow-wort as well, which is the best that can be done currently. In New York, black swallow-wort is not as common as pale, nor are the infestations of black as extensive as they are for pale swallow-wort, for whatever reason (Milbrath pers. comm.).

Score interpretation

While different users will have different concepts of what constitutes various levels of invasiveness (e.g., what is “highly invasive” vs. “moderately invasive” may differ among management agencies), we divided the ranks into six blocks in Appendix A. We consider species with scores ≥80 as “Extremely Invasive” and species with scores 70–79 as “Highly Invasive;” both of these groups are composed of species estimated to be very threaten­ing to Alberta. Species with scores of 60–69 as “Moderately Invasive” and scores of 50–59 represent “Modestly Invasive” species; both of these groups still pose significant risks to ecosystems. Species with scores of 40–49 are “Weakly Invasive”, and <40 are considered “Very Weakly Invasive.” These last two groups generally have not been shown to significantly alter ecosystem processes and communities elsewhere and probably do not require as much attention as the other species

**Alberta non-native plant invasiveness ranking form**

(Adapted from Carlson et al. 2008)

| Scientific name: | *Centaurea jacea* (inclusive of C. jacea complex) |
| --- | --- |
| Common name: | Brown knapweed |
| Assessor: | Shauna-Lee Chai |
| Reviewer: | Gerry Moore |
| Date: | December 2, 2013 |

Outcome score:

1. Climatic Comparison

This species is present or may potentially establish in the following natural regions:

|  | Collected in Alberta regions | CLIMEX similarity in 1975 | CLIMEX similarity in 2050 |
| --- | --- | --- | --- |
| Boreal | No | 0.835 | 0.857 |
| Parkland | No | 0.853 | 0.851 |
| Foothills | No | 0.875 | 0.882 |
| Grassland | No | 0.807 | 0.801 |
| Rocky Mountains | No | 0.872 | 0.866 |
| Shield | No | 0.784 | 0.820 |

1. Invasiveness Ranking Total (Total answered^1^ points possible) Total score

| 1. Ecological impact | 40(40) | 20 |
| --- | --- | --- |
| 1. Biological characteristic and dispersal ability | 25(25) | 13 |
| 1. Ecological amplitude and distribution | 25(25) | 18 |
| 1. Feasibility of control | 10(10) | 7 |
| Outcome score | 100(100)^b^ | ^a^58 |
| Relative maximum score^2^ | 58 | *Modestly Invasive* |

^1^For questions answered “unknown” do not include point value for the question in parentheses for “Total answered points possible.”

^2^Calculated as a/b x 100.

1. Climatic Comparison:
   1. Has this species ever been collected or documented in Alberta?

__Yes – continue to 1.2

x No – continue to 2.1

1.2 Which natural region has it been collected or documented? Proceed to section B. Invasiveness Ranking.

__Boreal

__Rockies

__Grassland

__Foothills

__Parkland

__Shield

Documentation:

Sources of information: ANPC Rogues gallery, ACIMS, PLANTS database, GBIF

2.1 Is there a 70 percent or higher similarity (based on CLIMEX climate matching) between climates anywhere the species currently occurs and

a. Boreal -Yes

b. Rockies - -Yes

c. Grassland -Yes

d. Foothills -Yes

e. Parkland -Yes

f. Shield -Yes

-If “no” is answered for all regions, reject species from consideration

Documentation:

Sources of information:

1. Invasiveness Ranking
2. Ecological Impact
   1. Impact on Natural Ecosystem Processes
3. No perceivable impact on ecosystem processes 0
4. Has the potential to influence ecosystem processes to a minor degree

(e.g., has a perceivable but mild influence on soil nutrient availability) 3

1. Has the potential to cause significant alteration of ecosystem processes (e.g., increases sedimentation rates along streams or coastlines, reduces open water

that are important to waterfowl) 7

1. May cause major, possibly irreversible, alteration or disruption of ecosystem processes (e.g., the species alters geomorphology; hydrology; or affects fire frequency, altering community composition; species fixes substantial levels of nitrogen in the soil making soil unlikely to support certain native plants or more likely to favor non-native species) 10

u. Unknown

Score:3

Documentation: taproot may change soil water content (Cal-IPC 2005)

Identify ecosystem processes impacted:

Rationale:

Sources of information:

- 1. Impact on Natural Community Structure

1. No perceived impact; establishes in an existing layer without influencing its

structure 0

1. Has the potential to influence structure in one layer (e.g., changes the density

of one layer) 3

1. Has the potential to cause significant impact in at least one layer (e.g., creation

of a new layer or elimination of an existing layer) 7

1. Likely to cause major alteration of structure (e.g., covers canopy, eradicating

most or all layers below) 10

1. Unknown

Score:7

Documentation: Can invade rangeland and pastures, causing reduction in carrying capacity. Forms dense stands. It can also result in reduced yields in hayfields. It out competes grasses and other pasture species and is difficult to control due to its extensive root system (Cal-IPC 2005)

Identify type of impact or alteration:

Rationale:

Sources of information:

- 1. Impact on Natural Community Composition

1. No perceived impact; causes no apparent change in native populations 0
2. Has the potential to influence community composition (e.g., reduces the

number of individuals in one or more native species in the community) 3

1. Has the potential to significantly alters community composition (e.g., produces

a significant reduction in the population size of one or more native species in

the community) 7

1. Likely to cause major alteration in community composition (e.g., results in the extirpation of one or several native species, reducing biodiversity or change the community composition towards species exotic to the natural community) 10

u. Unknown

Score:7

Documentation: Can invade rangeland and pastures, causing reduction in carrying capacity. Forms dense stands. It can also result in reduced yields in hayfields. It out competes grasses and other pasture species and is difficult to control due to its extensive root system (Cal-IPC 2005)

Identify type of impact or alteration:

Rationale:

Sources of information:

- 1. Impact on higher trophic levels (cumulative impact of this species on the animals,

fungi, microbes, and other organisms in the community it invades)

1. Negligible perceived impact 0
2. Has the potential to cause minor alteration 3
3. Has the potential to cause moderate alteration (minor reduction in

nesting/foraging sites, reduction in habitat connectivity, interference with

native pollinators, injurious components such as spines, toxins) 7

1. Likely to cause severe alteration of higher trophic populations (extirpation or endangerment of an existing native species/population, or significant reduction

in nesting or foraging sites) 10

u. Unknown

Score:3

Documentation: low palatability to grazers (Cal-IPC 2005)

Identify type of impact or alteration:

Rationale:

Sources of information:

Total Possible:40

Total:20

1. Biological Characteristics and Dispersal Ability
   1. Mode of reproduction
2. Not aggressive reproduction (few [0-10] seeds per plant and no

vegetative reproduction) 0

1. Somewhat aggressive (reproduces only by seeds (11-1,000/m2) 1
2. Moderately aggressive (reproduces vegetatively and/or by a moderate

amount of seed, <1,000/m2) 2

1. Highly aggressive reproduction (extensive vegetative spread and/or

many seeded, >1,000/m2) 3

u. Unknown

Score:3

Documentation: reproduces only by seed, producing >1000 per sq m (Cal-IPC 2005)

Describe key reproductive characteristics (including seeds per plant):

Rationale:

Sources of information:

- 1. Innate potential for long-distance dispersal (bird dispersal, sticks to animal hair, buoyant fruits, wind-dispersal)

1. Does not occur (no long-distance dispersal mechanisms) 0
2. Infrequent or inefficient long-distance dispersal (occurs occasionally

despite lack of adaptations) 2

1. Numerous opportunities for long-distance dispersal (species has

adaptations such as pappus, hooked fruit-coats, etc.) 3

1. Unknown

Score:2

Documentation:water, but rarely grows near to water (Cal-IPC 2005)

Identify dispersal mechanisms:

Rationale:

Sources of information:

- 1. Potential to be spread by human activities (both directly and indirectly – possible mechanisms include: commercial sales, use as forage/revegetation, spread along highways, transport on boats, contamination, etc.)

1. Does not occur 0
2. Low (human dispersal is infrequent or inefficient) 1
3. Moderate (human dispersal occurs) 2
4. High (there are numerous opportunities for dispersal to new areas) 3

u. Unknown

Score: 2

Documentation: dispersed by vehicles and is available for sale as an ornamental (AISC 2012)

Identify dispersal mechanisms:

Rationale:

Sources of information:

- 1. Allelopathic

1. no 0
2. yes 2
3. unknown

Score:0

Documentation:

Describe effect on adjacent plants:

Rationale:

Sources of information:

- 1. Competitive ability

a. Poor competitor for limiting factors 0

b. Moderately competitive for limiting factors 1

c. Highly competitive for limiting factors and/or nitrogen fixing ability 3

u. Unknown

Score:1

Documentation: competes well for water Cal-IPC 2005

Evidence of competitive ability:

Rationale:

Sources of information:

- 1. Forms dense thickets, climbing or smothering growth habit, or otherwise taller than the surrounding vegetation

1. No 0
2. Forms dense thickets 1
3. Has climbing or smothering growth habit, or otherwise taller than the surrounding

vegetation 2

u. Unknown

Score:1

Documentation:

Describe growth form:

Rationale:

Sources of information:

- 1. Germination requirements

1. Requires open soil and disturbance to germinate 0
2. Can germinate in vegetated areas but in a narrow range or in special conditions 2
3. Can germinate in existing vegetation in a wide range of conditions 3

u. Unknown

Score:0

Documentation:

Describe germination requirements:

Rationale:

Sources of information:

- 1. Other species in the genus invasive in Alberta or elsewhere

1. No 0
2. Yes 3

u. Unknown

Score:3

Documentation: Bighead, hybrid, Russian, Squarrose, Black, Diffuse, meadow, Spotted, Tyrol knapweeds

Species:

Sources of information:

2.9 Aquatic, wetland, or riparian species

a. Not invasive in wetland communities 0

b. Invasive in riparian communities 1

c. Invasive in wetland communities 3

u. Unknown

Score: 1

Documentation: Generally grows away from water (Cal-IPC 2005)

Describe type of habitat:

Rationale:

Sources of information:

Total Possible:25

Total:13

1. Distribution
   1. Is the species highly domesticated or a weed of agriculture
2. No 0
3. Is occasionally an agricultural pest 2
4. Has been grown deliberately, bred, or is known as a significant agricultural pest 4
5. Unknown

Score: 4

Documentation:ornamental

Identify reason for selection, or evidence of weedy history:

Rationale:

Sources of information:

- 1. Known level of ecological impact in natural areas

1. Not known to cause impact in any other natural area 0
2. Known to cause impacts in natural areas, but in dissimilar habitats and

climate zones than exist in regions of Alberta 1

1. Known to cause low impact in natural areas in similar habitats and climate

zones to those present in Alberta 3

1. Known to cause moderate impact in natural areas in similar habitat and

climate zones 4

1. Known to cause high impact in natural areas in similar habitat and climate

zones 6

u. Unknown

Score:3

Documentation: One example is the only known infestation in Colorado that is not overgrazed and has a riparian area that has been described as "very healthy." The existing native plant community is quite diverse and the infestation is in all the different plant communities. (Cal-IPC 2005)

Identify type of habitat and states or provinces where it occurs:

Sources of information:

- 1. Role of anthropogenic and natural disturbance in establishment

1. Requires anthropogenic disturbances to establish 0
2. May occasionally establish in undisturbed areas but can readily establish in

areas with natural disturbances 3

1. Can establish independent of any known natural or anthropogenic disturbances 5

u. Unknown

Score:3

Documentation: Grows in disturbed places, but does not need disturbance to establish new populations. One example is the only known infestation in Colorado that is not overgrazed and has a riparian area that has been described as "very healthy." The existing native plant community is quite diverse and the infestation is in all the different plant communities. (Cal-IPC 2005)

Identify type of disturbance:

Rationale:

Sources of information:

- 1. Current global distribution

1. Occurs in one or two continents or regions (e.g., Mediterranean region) 0
2. Extends over three or more continents 3
3. Extends over three or more continents, including successful introductions in

arctic or subarctic regions 5

u. Unknown

Score:3

Documentation: Europe, North and South America, Africa, Australia (ISSG)

Describe distribution:

Rationale:

Sources of information:

- 1. Extent of the species Canada range and/or occurrence of formal state or provincial listing

1. 0-5 percent of the states/provinces 0
2. 6-20 percent of the states/provinces 2
3. 21-50 percent, and/or state/province listed as a problem weed

(e.g., “Noxious,” or “Invasive”) in 1 state or Canadian province 4

1. Greater than 50 percent, and/or identified as “Noxious” in 2 or more states or

Canadian provinces 5

u. Unknown

Score:5

Documentation: noxious in Washington and Ontario and prohibited noxious is Alberta

Identify provinces invaded:

Rationale:

Sources of information:

Total possible:25

Total:18

1. Feasibility of Control
   1. Seed banks
2. Seeds remain viable in the soil for less than 3 years 0
3. Seeds remain viable in the soil for between 3 and 5 years 2
4. Seeds remain viable in the soil for 5 years and more 3

u. Unknown

Score:2

Documentation: Cal-IPC 2005

Identify longevity of seed bank

Rationale:

Sources of information:

- 1. Vegetative regeneration

1. No resprouting following removal of aboveground growth 0
2. Resprouting from ground-level meristems 1
3. Resprouting from extensive underground system 2
4. Any plant part is a viable propagule 3

u. Unknown

Score:1

Documentation: resprouts from root crown (AISC 2012)

Describe vegetative response:

Rationale:

Sources of information:

- 1. Level of effort required

1. Management is not required (e.g., species does not persist without repeated anthropogenic disturbance) 0
2. Management is relatively easy and inexpensive; requires a minor investment in human and financial resources 2
3. Management requires a major short-term investment of human and financial resources, or a moderate long-term investment 3
4. Management requires a major, long-term investment of human and financial resources 4

u. Unknown

Score:4

Documentation: Once a seed bank develops, management is required for many years (AISC 2012)

Identify types of control methods and time-term required: Mechanical

Rationale:

Sources of information:

Total Possible: 10

Total: 7

Total for 4 sections Possible: 100

Total for 4 sections: 58

References:

AISC 2012. Alberta Invasive Species Council Fact Sheet: Brown knapweed

Cal-IPC 2005. (California Invasive Plant Council) Plant Assessment Form. Available at: <http://www.cal-ipc.org/paf/site/paf/293>

Carlson, M. 2008. Invasiveness Ranking System for Non-Native Plants of Alaska. USDA. Available at: http://www.fs.usda.gov/Internet/FSE_DOCUMENTS/fsbdev2_037575.pdf

USDA PLANTS database

Notes

Taxonomy is difficult as there are closely related species in this complex, such as C. nigra, C. ×moncktonii, and C. nigrescens (Moore pers.comm.).

Similar to other knapweed species. It prefers moister and cooler conditions than other knapweeds, growing in moister grasslands and open forests. It tolerates partial shade.

Score Interpretation

While different users will have different concepts of what constitutes various levels of invasiveness (e.g., what is “highly invasive” vs. “moderately invasive” may differ among management agencies), we divided the ranks into six blocks in Appendix A. We consider species with scores ≥80 as “Extremely Invasive” and species with scores 70–79 as “Highly Invasive;” both of these groups are composed of species estimated to be very threaten­ing to Alberta. Species with scores of 60–69 as “Moderately Invasive” and scores of 50–59 represent “Modestly Invasive” species; both of these groups still pose significant risks to ecosystems. Species with scores of 40–49 are “Weakly Invasive”, and <40 are considered “Very Weakly Invasive.” These last two groups generally have not been shown to significantly alter ecosystem processes and communities elsewhere and probably do not require as much attention as the other species.

**Alberta non-native plant invasiveness ranking form**

(Adapted from Carlson et al. 2008)

| Scientific name: | *Fallopia sachalinensis/Polygonum sachalinense* |
| --- | --- |
| Common name: | Giant Knotweed |
| Assessor: | Shauna-Lee Chai |
| Reviewer: | Thomas Heutte |
| Date: | November 8, 2013 |

Outcome score:

1. Climatic Comparison

This species is present or may potentially establish in the following natural regions:

|  | Collected in Alberta regions | CLIMEX similarity in 1975 | CLIMEX similarity in 2050 |
| --- | --- | --- | --- |
| Boreal | No | 0.776 | 0.821 |
| Parkland | No | 0.825 | 0.834 |
| Foothills | No | 0.843 | 0.869 |
| Grassland | No | 0.795 | 0.809 |
| Rocky Mountains | No | 0.828 | 0.838 |
| Shield | No | 0.701 | 0.764 |

*present in gardens as ornamentals

1. Invasiveness Ranking Total (Total answered^1^ points possible) Total score

| 1. Ecological impact | 40(40) | 31 |
| --- | --- | --- |
| 1. Biological characteristic and dispersal ability | 25(25) | 18 |
| 1. Ecological amplitude and distribution | 25(25) | 23 |
| 1. Feasibility of control | 10(7) | 6 |
| Outcome score | 100(97)^b^ | ^a^78 |
| Relative maximum score^2^ | 80 | *Extremely Invasive* |

^1^For questions answered “unknown” do not include point value for the question in parentheses for “Total answered points possible.”

^2^Calculated as a/b x 100.

1. Climatic Comparison:
   1. Has this species ever been collected or documented in Alberta?

__Yes – continue to 1.2

x No – continue to 2.1

1.2 Which natural region has it been collected or documented (see inset map)? Proceed to section B. Invasiveness Ranking.

__Boreal

__Rockies

__Grassland

__Foothills

__Parkland

__Shield

Documentation:

Sources of information: ANPC Rogues gallery, ACIMS, PLANTS database, GBIF

2.1 Is there a 70 percent or higher similarity (based on CLIMEX climate matching) between climates anywhere the species currently occurs and

a. Boreal -Yes

b. Rockies - Yes

c. Grassland -Yes

d. Foothills -Yes

e. Parkland -Yes

f. Shield -Yes

-If “no” is answered for all regions, reject species from consideration

Documentation:

Sources of information:

1. Invasiveness Ranking
2. Ecological Impact
   1. Impact on Natural Ecosystem Processes
3. No perceivable impact on ecosystem processes 0
4. Has the potential to influence ecosystem processes to a minor degree

(e.g., has a perceivable but mild influence on soil nutrient availability) 3

1. Has the potential to cause significant alteration of ecosystem processes (e.g., increases sedimentation rates along streams or coastlines, reduces open water

that are important to waterfowl) 7

1. May cause major, possibly irreversible, alteration or disruption of ecosystem processes (e.g., the species alters geomorphology; hydrology; or affects fire frequency, altering community composition; species fixes substantial levels of nitrogen in the soil making soil unlikely to support certain native plants or more likely to favor non-native species) 10

u. Unknown

Score: 10

Documentation: Ecological impacts of the 3 knotweeds include reduced recruitment of in-stream woody debris and reduced habitat quality for wildlife. Establishment of these knotweeds may also increase the risk of streambank erosion or flooding when decaying shoots are washed into rivers during high flows (Niewinski 1998). Reviews by areas dominated by giant knotweed had less nitrogen available for uptake by both terrestrial and aquatic organisms than areas dominated by native species (Urgenson et al. 2009)

Identify ecosystem processes impacted:

Rationale:

Sources of information:

- 1. Impact on Natural Community Structure

1. No perceived impact; establishes in an existing layer without influencing its

structure 0

1. Has the potential to influence structure in one layer (e.g., changes the density

of one layer) 3

1. Has the potential to cause significant impact in at least one layer (e.g., creation

of a new layer or elimination of an existing layer) 7

1. Likely to cause major alteration of structure (e.g., covers canopy, eradicating

most or all layers below) 10

1. Unknown

Score:7

Documentation: One study supported the assertion that giant knotweed displaces riparian species and has cascading effects on the structure and function of riparian systems. In northwestern Washington, riparian forests with higher giant knotweed stem density had lower juvenile conifer (P<0.01), juvenile red alder (P<0.001), juvenile broadleaved tree (P<0.001), and shrub (P<0.01) stem density; lower herb (P<0.01) and native herb (P<0.001) cover; and lower shrub (P=0.001), herb (P<0.001), and native herb (P=0.002) species richness compared to forests with lower giant knotweed stem density (Urgenson et al. 2009)

Identify type of impact or alteration:

Rationale:

Sources of information:

- 1. Impact on Natural Community Composition

1. No perceived impact; causes no apparent change in native populations 0
2. Has the potential to influence community composition (e.g., reduces the

number of individuals in one or more native species in the community) 3

1. Has the potential to significantly alters community composition (e.g., produces

a significant reduction in the population size of one or more native species in

the community) 7

1. Likely to cause major alteration in community composition (e.g., results in the extirpation of one or several native species, reducing biodiversity or change the community composition towards species exotic to the natural community) 10

u. Unknown

Score:7

Documentation: Studies in both North America and Europe have documented a decrease in native plant cover or species richness in areas where giant knotweed occurs (Hejda et al. 2009). Mechanisms suggested for native plant exclusion include the accumulation of leaf and stem litter, nutrient limitation, and allelopathy (Vrchotova & Sera 2008)

Identify type of impact or alteration:

Rationale:

Sources of information:

- 1. Impact on higher trophic levels (cumulative impact of this species on the animals,

fungi, microbes, and other organisms in the community it invades)

1. Negligible perceived impact 0
2. Has the potential to cause minor alteration 3
3. Has the potential to cause moderate alteration (minor reduction in

nesting/foraging sites, reduction in habitat connectivity, interference with

native pollinators, injurious components such as spines, toxins) 7

1. Likely to cause severe alteration of higher trophic populations (extirpation or endangerment of an existing native species/population, or significant reduction

in nesting or foraging sites) 10

u. Unknown

Score: 7

Documentation: Studies in North America and Europe have documented changes in faunal communities, including a decrease in the diversity and abundance of invertebrates (Kappes et al 2007). Reduces habitat quality for wildlife (Niewinski 1998)

Identify type of impact or alteration:

Rationale:

Sources of information:

Total Possible:40

Total:31

1. Biological Characteristics and Dispersal Ability
   1. Mode of reproduction
2. Not aggressive reproduction (few [0-10] seeds per plant and no

vegetative reproduction) 0

1. Somewhat aggressive (reproduces only by seeds (11-1,000/m2) 1
2. Moderately aggressive (reproduces vegetatively and/or by a moderate

amount of seed, <1,000/m2) 2

1. Highly aggressive reproduction (extensive vegetative spread and/or

many seeded, >1,000/m2) 3

u. Unknown

Score:3

Documentation: In areas with all 3 knotweeds in Pennsylvania, single stems produced 50,000 to 150,000 seeds annually; millions of seeds were produced over a 108-ft² (10-m²) area in some locations (Niewinski, 1998). In Pennsylvania, viable seeds of the 3 knotweeds were not found when male-fertile plants were absent from the site, but many viable seeds were produced when both female and male-fertile plants were present (Niewinski, 1998).

Describe key reproductive characteristics (including seeds per plant):

Rationale:

Sources of information:

- 1. Innate potential for long-distance dispersal (bird dispersal, sticks to animal hair, buoyant fruits, wind-dispersal)

1. Does not occur (no long-distance dispersal mechanisms) 0
2. Infrequent or inefficient long-distance dispersal (occurs occasionally

despite lack of adaptations) 2

1. Numerous opportunities for long-distance dispersal (species has

adaptations such as pappus, hooked fruit-coats, etc.) 3

1. Unknown

Score:3

Documentation: Seeds of the 3 knotweeds may be dispersed by wind, birds, insects, or water

Identify dispersal mechanisms:

Rationale:

Sources of information:

- 1. Potential to be spread by human activities (both directly and indirectly – possible mechanisms include: commercial sales, use as forage/revegetation, spread along highways, transport on boats, contamination, etc.)

1. Does not occur 0
2. Low (human dispersal is infrequent or inefficient) 1
3. Moderate (human dispersal occurs) 2
4. High (there are numerous opportunities for dispersal to new areas) 3

u. Unknown

Score:2

Documentation: Humans spread the plants through dumping yard waste, roadside mowing or construction projects, or using fill dirt from riparian areas

Identify dispersal mechanisms:

Rationale:

Sources of information:

- 1. Allelopathic

1. no 0
2. yes 2
3. unknown

Score:2

Documentation: Vrchotova, 2008

Describe effect on adjacent plants:

Rationale:

Sources of information:

- 1. Competitive ability

a. Poor competitor for limiting factors 0

b. Moderately competitive for limiting factors 1

c. Highly competitive for limiting factors and/or nitrogen fixing ability 3

u. Unknown

Score:1

Documentation: areas dominated by giant knotweed had less nitrogen available for uptake by both terrestrial and aquatic organisms than areas dominated by native species (Urgenson et al. 2009)

Evidence of competitive ability:

Rationale:

Sources of information:

- 1. Forms dense thickets, climbing or smothering growth habit, or otherwise taller than the surrounding vegetation

1. No 0
2. Forms dense thickets 1
3. Has climbing or smothering growth habit, or otherwise taller than the surrounding

vegetation 2

u. Unknown

Score:1

Documentation:

Describe growth form:

Rationale:

Sources of information:

- 1. Germination requirements

1. Requires open soil and disturbance to germinate 0
2. Can germinate in vegetated areas but in a narrow range or in special conditions 2
3. Can germinate in existing vegetation in a wide range of conditions 3

u. Unknown

Score:0

Documentation: Giant knotweed seed germination is favored by moisture and disturbance (Niewinski 1998)

Describe germination requirements:

Rationale:

Sources of information:

- 1. Other species in the genus invasive in Alberta or elsewhere

1. No 0
2. Yes 3

u. Unknown

Score:3

Documentation: Polygonum cuspidatum Siebold & Zucc., Japanese knotweed. Hybrids of giant and Japanese produce Polygonum bohemicum, possibly more invasive than its parents due to greater genetic diversity.

Species:

Sources of information:

2.9 Aquatic, wetland, or riparian species

a. Not invasive in wetland communities 0

b. Invasive in riparian communities 1

c. Invasive in wetland communities 3

u. Unknown

Score:3

Documentation: Plants of all 3 knotweeds that escape cultivation and establish in riparian areas may spread when plant parts are transported downstream (Pysek and Prach 1993). In California, giant knotweed occurred in riparian areas, with the most severe impacts in wetlands in the northwestern part of the state (California Invasive Plant Council. 2006)

Describe type of habitat:

Rationale:

Sources of information:

Total Possible:25

Total:18

1. Distribution
   1. Is the species highly domesticated or a weed of agriculture
2. No 0
3. Is occasionally an agricultural pest 2
4. Has been grown deliberately, bred, or is known as a significant agricultural pest 4
5. Unknown

Score:4

Documentation: Introduced as ornamental plant. Giant knotweed was also promoted as a soil binder and fodder plant (FNA 2010)

Identify reason for selection, or evidence of weedy history:

Rationale:

Sources of information:

- 1. Known level of ecological impact in natural areas

1. Not known to cause impact in any other natural area 0
2. Known to cause impacts in natural areas, but in dissimilar habitats and

climate zones than exist in regions of Alberta 1

1. Known to cause low impact in natural areas in similar habitats and climate

zones to those present in Alberta 3

1. Known to cause moderate impact in natural areas in similar habitat and

climate zones 4

1. Known to cause high impact in natural areas in similar habitat and climate

zones 6

u. Unknown

Score:6

Documentation: Japanese knotweed has invaded rivers bars in Sitka National Historical Park (Densmore et al. 2001) and has established additional infestations in the Tongass National Forest (Stensvold 2000). Large stands have been found along the riverbanks in Pennsylvania and Ohio (Seiger 1991).

Identify type of habitat and states or provinces where it occurs:

Sources of information:

- 1. Role of anthropogenic and natural disturbance in establishment

1. Requires anthropogenic disturbances to establish 0
2. May occasionally establish in undisturbed areas but can readily establish in

areas with natural disturbances 3

1. Can establish independent of any known natural or anthropogenic disturbances 5

u. Unknown

Score:3

Documentation: Japanese knotweed can establish in native habitats (Stensvold 2000, Shaw and Seiger 2002).

Identify type of disturbance:

Rationale:

Sources of information:

- 1. Current global distribution

1. Occurs in one or two continents or regions (e.g., Mediterranean region) 0
2. Extends over three or more continents 3
3. Extends over three or more continents, including successful introductions in

arctic or subarctic regions 5

u. Unknown

Score:5

Documentation: Asia, North America (including Alaska), Australia, South Africa, Europe

Describe distribution:

Rationale:

Sources of information:

- 1. Extent of the species Canada range and/or occurrence of formal state or provincial listing

1. 0-5 percent of the states/provinces 0
2. 6-20 percent of the states/provinces 2
3. 21-50 percent, and/or state/province listed as a problem weed

(e.g., “Noxious,” or “Invasive”) in 1 state or Canadian province 4

1. Greater than 50 percent, and/or identified as “Noxious” in 2 or more states or

Canadian provinces 5

u. Unknown

Score:5

Documentation: Noxious in California, Connecticut, Oregon, Washington (USDA PLANTS)

Identify provinces invaded:

Rationale:

Sources of information:

Total possible:25

Total:23

1. Feasibility of Control
   1. Seed banks
2. Seeds remain viable in the soil for less than 3 years 0
3. Seeds remain viable in the soil for between 3 and 5 years 2
4. Seeds remain viable in the soil for 5 years and more 3

u. Unknown

Score:unknown

Documentation: unknown (Stone 2010)

Spread by seed is rare, though it has been suggested for Japanese knotweed, and seedlings of giant, Japanese, and Bohemian knotweed have been observed (Niewinski 1998).

Identify longevity of seed bank

Rationale:

Sources of information:

- 1. Vegetative regeneration

1. No resprouting following removal of aboveground growth 0
2. Resprouting from ground-level meristems 1
3. Resprouting from extensive underground system 2
4. Any plant part is a viable propagule 3

u. Unknown

Score:3

Documentation: The ability for multiple plant parts to regenerate vegetatively plays an important role in the spread and establishment of the 3 knotweeds. Vegetative regeneration is possible from multiple plant parts, including rhizomes, aboveground stems, roots, and leaves (Stone 2010).

Describe vegetative response:

Rationale:

Sources of information:

- 1. Level of effort required

1. Management is not required (e.g., species does not persist without repeated anthropogenic disturbance) 0
2. Management is relatively easy and inexpensive; requires a minor investment in human and financial resources 2
3. Management requires a major short-term investment of human and financial resources, or a moderate long-term investment 3
4. Management requires a major, long-term investment of human and financial resources 4

u. Unknown

Score:3

Documentation: It responds well to herbicide application, although follow-up applications are required (Heutte pers. comm.). Several sources suggest that the 3 knotweeds are difficult to eradicate due to their extensive root and rhizome systems, the ability of multiple plant parts to regenerate vegetatively, sprouting immediately or 1 to 3 years after treatment, and the large scale of stand establishment. Control of the 3 knotweeds may require multiple treatments within a single growing season or several years of treatment to be effective. Careful disposal of removed plant parts is important to prevent downstream transport or reestablishment. Control and eradication efforts always face the potential for floods or high water to expose and/or transport buried rhizomes or propagules from upstream populations (Davenport 2006).

Identify types of control methods and time-term required: mowing, digging, covering, and many methods of herbicide application

Rationale:

Sources of information:

Total Possible: 7

Total:6

Total for 4 sections Possible: 97

Total for 4 sections: 78

References:

Carlson, M. 2008. Invasiveness Ranking System for Non-Native Plants of Alaska. USDA. Available at: http://www.fs.usda.gov/Internet/FSE_DOCUMENTS/fsbdev2_037575.pdf

California Invasive Plant Council. 2006. California invasive plant inventory, [Online]. California Invasive Plant Council (Producer). Available: http://www.cal-ipc.org/ip/inventory/pdf/Inventory2006.pdf.

Davenport, Roberta. 2006. Control of knotweed and other invasive species and experiences restoring native species in the Pacific Northwest US. Native Plants Journal. 7(1): 20-26.

Densmore, R.V., P.C. McKee, and C. Roland. 2001. Exotic plants in Alaskan National Park Units. Report on file with the National Park Service – Alaska Region, Anchorage, Alaska. 143 pp.

Flora of North America Association. 2010. Flora of North America: The flora, [Online]. Flora of North America Association

Hejda, Martin; Pysek, Petr; Jarosik, Vojtech. 2009. Impact of invasive plants on the species richness, diversity and composition of invaded communities. Journal of Ecology. 97: 393-403

Kappes, Heike; Lay, Rebecca; Topp, Werner. 2007. Changes in different trophic levels of litter-dwelling macrofauna associated with giant knotweed invasion. Ecosystems. 10(5): 734-744

Niewinski, Amy Thomas. 1998. The reproductive ecology of Japanese knotweed (Polygonum cuspidatum) and giant knotweed (Polygonum sachalinensis) seed. University Park, PA: Pennsylvania State University. 49 p. Thesis

Pysek, Petr; Prach, Karel. 1993. Plant invasions and the role of riparian habitats: comparison of four species alien to central Europe. Journal of Biogeography. 20(4): 413-420.

Seiger, L. 1991. Element Stewardship Abstract for Polygonum cuspidatum. The Nature Conservancy in collaboration with the International Network of Natural Heritage Programs and Conservation Data Centers. Natural Heritage Databases. Arlington, VA.

Stensvold, M. 2000. Noxious weed surveys and projects conducted on the Tongass National Forest 1997-2000. Technical report on file, Tongass National Forest. 2 pp.

Urgenson, Lauren S.; Reichard, Sarah H.; Halpern, Charles B. 2009. Community and ecosystem consequences of giant knotweed (Polygonum sachalinense) invasion into riparian forests of western Washington, USA. Biological Conservation. 142(7): 1536-1541.

USDA PLANTS database

Shaw R.H. and L.A. Seiger. 2002. Japanese Knotweed – Biological Control of Invasive Plants in the Eastern United States. In Van Driesche, R. et al., 2002. Biological Control of Invasive Plants in the Eastern United States, USDA Forest Service Publication FHTET-2002-04, 413 pp.

Stensvold, M. 2000. Noxious weed surveys and projects conducted on the Tongass National Forest 1997-2000. Technical report on file, Tongass National Forest. 2 pp.

Stone, Katharine R. 2010. Polygonum sachalinense, P. cuspidatum, P. × bohemicum. In: Fire Effects Information System, [Online]. U.S. Department of Agriculture, Forest Service, Rocky Mountain Research Station, Fire Sciences Laboratory (Producer). Available: http://www.fs.fed.us/database/feis/ [2013, November 29].

Vrchotova, N.; Sera, B. 2008. Allelopathic properties of knotweed rhizome extracts. Plant, Soil and Environment. 54(7): 301-303.

Notes

Giant knotweed is a stout, rhizomatous, perennial geophyte. Observations from the Czech Republic suggest that giant knotweed roots grow "deep" into the soil. Rhizomes have a diameter of 3 inches (8 cm) and may spread 50 to 65 feet (15-20 m) laterally.

Score Interpretation

While different users will have different concepts of what constitutes various levels of invasiveness (e.g., what is “highly invasive” vs. “moderately invasive” may differ among management agencies), we divided the ranks into six blocks in Appendix A. We consider species with scores ≥80 as “Extremely Invasive” and species with scores 70–79 as “Highly Invasive;” both of these groups are composed of species estimated to be very threaten­ing to Alberta. Species with scores of 60–69 as “Moderately Invasive” and scores of 50–59 represent “Modestly Invasive” species; both of these groups still pose significant risks to ecosystems. Species with scores of 40–49 are “Weakly Invasive”, and <40 are considered “Very Weakly Invasive.” These last two groups generally have not been shown to significantly alter ecosystem processes and communities elsewhere and probably do not require as much attention as the other species.

**Alberta non-native plant invasiveness ranking form**

(Adapted from Carlson et al. 2008)

| Scientific name: | *Echinops sphaerocephalus* |
| --- | --- |
| Common name: | Globe thistle/Arctic glow |
| Assessor: | Shauna-Lee Chai |
| Reviewer: | Kurt Dreisilker |
| Date: | November 6, 2013 |

Outcome score:

1. Climatic Comparison

This species is present or may potentially establish in the following natural regions:

|  | Collected in Alberta regions | CLIMEX similarity in 1975 | CLIMEX similarity in 2050 |
| --- | --- | --- | --- |
| Boreal | No | 0.739 | 0.795 |
| Parkland | No | 0.806 | 0.834 |
| Foothills | No | 0.828 | 0.862 |
| Grassland | Yes | 0.832 | 0.858 |
| Rocky Mountains | No | 0.781 | 0.809 |
| Shield | No | 0.647 | 0.720 |

*train tracks in Calgary

1. Invasiveness Ranking Total (Total answered^1^ points possible) Total score

| 1. Ecological impact | 40(27) | 6 |
| --- | --- | --- |
| 1. Biological characteristic and dispersal ability | 25(20) | 13 |
| 1. Ecological amplitude and distribution | 25(25) | 17 |
| 1. Feasibility of control | 10(7) | 5 |
| Outcome score | 100(79)^b^ | ^a^41 |
| Relative maximum score^2^ | 52 | *Modestly Invasive* |

^1^For questions answered “unknown” do not include point value for the question in parentheses for “Total answered points possible.”

^2^Calculated as a/b x 100.

1. Climatic Comparison:
   1. Has this species ever been collected or documented in Alberta?

x Yes – continue to 1.2

No – continue to 2.1

1.2 Which natural region has it been collected or documented? Proceed to section B. Invasiveness Ranking.

__Boreal

__Rockies

x Grassland

__Foothills

__Parkland

__Shield

Documentation:

Sources of information: ANPC Rogues gallery, ACIMS, PLANTS database, GBIF

2.1 Is there a 70 percent or higher similarity (based on CLIMEX climate matching) between climates anywhere the species currently occurs and

a. Boreal - Yes

b. Rockies - Yes

c. Grassland - Yes

d. Foothills - Yes

e. Parkland - Yes

f. Shield – Not in 1975, but in 2050

-If “no” is answered for all regions, reject species from consideration

Documentation:

Sources of information:

1. Invasiveness Ranking
2. Ecological Impact
   1. Impact on Natural Ecosystem Processes
3. No perceivable impact on ecosystem processes 0
4. Has the potential to influence ecosystem processes to a minor degree

(e.g., has a perceivable but mild influence on soil nutrient availability) 3

1. Has the potential to cause significant alteration of ecosystem processes (e.g., increases sedimentation rates along streams or coastlines, reduces open water

that are important to waterfowl) 7

1. May cause major, possibly irreversible, alteration or disruption of ecosystem processes (e.g., the species alters geomorphology; hydrology; or affects fire frequency, altering community composition; species fixes substantial levels of nitrogen in the soil making soil unlikely to support certain native plants or more likely to favor non-native species) 10

u. Unknown

Score:unknown

Documentation: likely low

Identify ecosystem processes impacted:

Rationale:

Sources of information:

- 1. Impact on Natural Community Structure

1. No perceived impact; establishes in an existing layer without influencing its

structure 0

1. Has the potential to influence structure in one layer (e.g., changes the density

of one layer) 3

1. Has the potential to cause significant impact in at least one layer (e.g., creation

of a new layer or elimination of an existing layer) 7

1. Likely to cause major alteration of structure (e.g., covers canopy, eradicating

most or all layers below) 10

1. Unknown

Score:3

Documentation: In NE Illinois, invaded areas mostly have low density or have disappeared from their historical introduction site (Dreisilker pers. comm.). Found to dominate herb layer up to 38% in Czech Republic (Petrik et al. 2009)

Identify type of impact or alteration:

Rationale:

Sources of information:

- 1. Impact on Natural Community Composition

1. No perceived impact; causes no apparent change in native populations 0
2. Has the potential to influence community composition (e.g., reduces the

number of individuals in one or more native species in the community) 3

1. Has the potential to significantly alters community composition (e.g., produces

a significant reduction in the population size of one or more native species in

the community) 7

1. Likely to cause major alteration in community composition (e.g., results in the extirpation of one or several native species, reducing biodiversity or change the community composition towards species exotic to the natural community) 10

u. Unknown

Score:3

Documentation: In NE Illinois, invaded areas mostly have low density or have disappeared from their historical introduction site (Dreisilker 2012). Due to dominance, it results in impoverishment of vegetation in Czech Republic (Petrik et al. 2009)

Identify type of impact or alteration:

Rationale:

Sources of information:

- 1. Impact on higher trophic levels (cumulative impact of this species on the animals,

fungi, microbes, and other organisms in the community it invades)

1. Negligible perceived impact 0
2. Has the potential to cause minor alteration 3
3. Has the potential to cause moderate alteration (minor reduction in

nesting/foraging sites, reduction in habitat connectivity, interference with

native pollinators, injurious components such as spines, toxins) 7

1. Likely to cause severe alteration of higher trophic populations (extirpation or endangerment of an existing native species/population, or significant reduction

in nesting or foraging sites) 10

u. Unknown

Score: unknown

Documentation:

Identify type of impact or alteration:

Rationale:

Sources of information:

Total Possible:27

Total:6

1. Biological Characteristics and Dispersal Ability
   1. Mode of reproduction
2. Not aggressive reproduction (few [0-10] seeds per plant and no

vegetative reproduction) 0

1. Somewhat aggressive (reproduces only by seeds (11-1,000/m2) 1
2. Moderately aggressive (reproduces vegetatively and/or by a moderate

amount of seed, <1,000/m2) 2

1. Highly aggressive reproduction (extensive vegetative spread and/or

many seeded, >1,000/m2) 3

u. Unknown

Score:3

Documentation: Reproduction is mainly by seed, with 1400 seeds produced per plant, and up to 5075 propagules per m2 (Moravcova 2010).

Describe key reproductive characteristics (including seeds per plant):

Rationale:

Sources of information:

- 1. Innate potential for long-distance dispersal (bird dispersal, sticks to animal hair, buoyant fruits, wind-dispersal)

1. Does not occur (no long-distance dispersal mechanisms) 0
2. Infrequent or inefficient long-distance dispersal (occurs occasionally

despite lack of adaptations) 2

1. Numerous opportunities for long-distance dispersal (species has

adaptations such as pappus, hooked fruit-coats, etc.) 3

1. Unknown

Score:2

Documentation: wind dispersed

Identify dispersal mechanisms:

Rationale:

Sources of information:

- 1. Potential to be spread by human activities (both directly and indirectly – possible mechanisms include: commercial sales, use as forage/revegetation, spread along highways, transport on boats, contamination, etc.)

1. Does not occur 0
2. Low (human dispersal is infrequent or inefficient) 1
3. Moderate (human dispersal occurs) 2
4. High (there are numerous opportunities for dispersal to new areas) 3

u. Unknown

Score: 2

Documentation: cultivated and spread by humans as a honey producing plant becoming naturalised in the Czech Republic (Petrik et al. 2009). Horticultural interest-recent references of this species and related species being used as ornamental herbs although, it’s not all that common to use this species in NE Illinois as an ornamental plant (Dreisilker pers. comm.).

Identify dispersal mechanisms:

Rationale:

Sources of information:

- 1. Allelopathic

1. no 0
2. yes 2
3. unknown

Score:unknown

Documentation:

Describe effect on adjacent plants:

Rationale:

Sources of information:

- 1. Competitive ability

a. Poor competitor for limiting factors 0

b. Moderately competitive for limiting factors 1

c. Highly competitive for limiting factors and/or nitrogen fixing ability 3

u. Unknown

Score:unknown

Documentation:

Evidence of competitive ability:

Rationale:

Sources of information:

- 1. Forms dense thickets, climbing or smothering growth habit, or otherwise taller than the surrounding vegetation

1. No 0
2. Forms dense thickets 1
3. Has climbing or smothering growth habit, or otherwise taller than the surrounding

vegetation 2

u. Unknown

Score:0

Documentation: This hasn’t formed a dense, impenetrable thicket as a shrub patch would. Nor does it have a climbing or smothering growth habit. However, it was found growing in an area with shrubs, where the Echinops appeared to be outcompeted by the growing woody shrubs and trees as though the woody plants would eventually push the Echniops sp. out of the site. The Echinops sp. was often leaning away from overhanging shrub growth as though it was trying to “reach” for the sunlight (Dreisilker pers.comm.).

Describe growth form:

Rationale:

Sources of information:

- 1. Germination requirements

1. Requires open soil and disturbance to germinate 0
2. Can germinate in vegetated areas but in a narrow range or in special conditions 2
3. Can germinate in existing vegetation in a wide range of conditions 3

u. Unknown

Score:3

Documentation: Can germinate in full sun to partial shade and decreases with increasing shade (Dreisilker 2012).I germinated seeds from local two local populations. 100 seeds from the two populations were planted in trays and placed in cold storage. Germination soil mix consisted of 55% Canadian sphagnum, 20% perlite, 25% vermiculite. After 70 days of cold storage the trays were placed in the greenhouse to germinate. Ambient temperature ranged 65-68 degrees (F) with 18 hours of sunlight (natural and artificial). High rates of seed germination (up to 75%) were observed within 7 days (Dreisilker pers.comm.).

Describe germination requirements:

Rationale:

Sources of information:

- 1. Other species in the genus invasive in Alberta or elsewhere

1. No 0
2. Yes 3

u. Unknown

Score:3

Documentation: Echinops exaltatus invasive in Sweden <http://www.nobanis.org/speciesInfo.asp?taxaID=2161> . E. exaltatus, E. ritro, and E. sphaerocephalus have been introduced to North America) (Fl. of N. Am. 2011).

Species:

Sources of information:

2.9 Aquatic, wetland, or riparian species

a. Not invasive in wetland communities 0

b. Invasive in riparian communities 1

c. Invasive in wetland communities 3

u. Unknown

Score:0

Documentation: (Dreisilker pers.comm.).

Describe type of habitat:

Rationale:

Sources of information:

Total Possible:20

Total:13

1. Distribution
   1. Is the species highly domesticated or a weed of agriculture
2. No 0
3. Is occasionally an agricultural pest 2
4. Has been grown deliberately, bred, or is known as a significant agricultural pest 4
5. Unknown

Score:4

Documentation: Cultivated and can escape cultivation (FNO 1993). Cultivated as a honey producing plant (Petrik et al. 2009)

Identify reason for selection, or evidence of weedy history:

Rationale:

Sources of information:

- 1. Known level of ecological impact in natural areas

1. Not known to cause impact in any other natural area 0
2. Known to cause impacts in natural areas, but in dissimilar habitats and

climate zones than exist in regions of Alberta 1

1. Known to cause low impact in natural areas in similar habitats and climate

zones to those present in Alberta 3

1. Known to cause moderate impact in natural areas in similar habitat and

climate zones 4

1. Known to cause high impact in natural areas in similar habitat and climate

zones 6

u. Unknown

Score:3

Documentation: The species can invade both mesophilous species-poorer, human-influenced and natural communities. Recently, the invasion of E. sphaero cephalus to the species-rich, dry plant communities of the Sedo-Scleranthetea and Festuco-Brometea classes was documented. However, in these communities, E. sphaerocephalus is usually not a dominant species (Petrik et al. 2009). Low impact on natural areas in Illinois (Dreisilker pers.comm.).

Diagnostic

Identify type of habitat and states or provinces where it occurs:

Sources of information:

- 1. Role of anthropogenic and natural disturbance in establishment

1. Requires anthropogenic disturbances to establish 0
2. May occasionally establish in undisturbed areas but can readily establish in

areas with natural disturbances 3

1. Can establish independent of any known natural or anthropogenic disturbances 5

u. Unknown

Score:3

Documentation: Establishes in both human-influenced and natural communities (Petrik et al. 2009).

Identify type of disturbance:

Rationale:

Sources of information:

- 1. Current global distribution

1. Occurs in one or two continents or regions (e.g., Mediterranean region) 0
2. Extends over three or more continents 3
3. Extends over three or more continents, including successful introductions in

arctic or subarctic regions 5

u. Unknown

Score:3

Documentation: Europe, Asia, North America. Globe thistle is native to central and southern Europe, central Asia (Flora of North America 2011, Enc. of Life 2011, & Hortus Third 1976). Globe thistle is documented 22 states around the United States, ranging from the Pacific coast, central Rockies, Great Lakes, and New England regions. However, it is largely absent from the southern and southwestern states [United States Department of Agriculture (USDA) 2011]. Although it initially seems to be widespread throughout the United States, a closer inspection of its distribution within those states reveals that it is only reported in isolated, geographically dispersed counties within each state (USDA 2011).

Describe distribution:

Rationale:

Sources of information:

- 1. Extent of the species Canada range and/or occurrence of formal state or provincial listing

1. 0-5 percent of the states/provinces 0
2. 6-20 percent of the states/provinces 2
3. 21-50 percent, and/or state/province listed as a problem weed

(e.g., “Noxious,” or “Invasive”) in 1 state or Canadian province 4

1. Greater than 50 percent, and/or identified as “Noxious” in 2 or more states or

Canadian provinces 5

u. Unknown

Score:4

Documentation: Not noxious in any state or province. According to Flora of North America it is introduced in; Man., Ont., Que., Sask. Globe thistle is documented 22 states around the United States, ranging from the Pacific coast, central Rockies, Great Lakes, and New England regions. http://www.efloras.org/florataxon.aspx?flora_id=1&taxon_id=200023870

Identify provinces invaded:

Rationale:

Sources of information:

Total possible:25

Total:17

1. Feasibility of Control
   1. Seed banks
2. Seeds remain viable in the soil for less than 3 years 0
3. Seeds remain viable in the soil for between 3 and 5 years 2
4. Seeds remain viable in the soil for 5 years and more 3

u. Unknown

Score:unknown

Documentation:

Identify longevity of seed bank

Rationale:

Sources of information:

- 1. Vegetative regeneration

1. No resprouting following removal of aboveground growth 0
2. Resprouting from ground-level meristems 1
3. Resprouting from extensive underground system 2
4. Any plant part is a viable propagule 3

u. Unknown

Score:2

Documentation: I observed this species resprouting after being mowed. It appeared to be resprouting from the taproot (Dreisilker pers. comm)

Describe vegetative response: The mowing process that I observed removed the plant above the ground at a height of about 4 inches. Then the plant resprouted growth from the taproot and was capable of flowering (Dreisilker pers. comm).

Rationale:

Sources of information:

- 1. Level of effort required

1. Management is not required (e.g., species does not persist without repeated anthropogenic disturbance) 0
2. Management is relatively easy and inexpensive; requires a minor investment in human and financial resources 2
3. Management requires a major short-term investment of human and financial resources, or a moderate long-term investment 3
4. Management requires a major, long-term investment of human and financial resources 4

u. Unknown

Score:3

Documentation: Land managers in NE Illinois do not appear to consistently control globe thistle where it is currently found. Some land managers control it, while others don’t. Those who control it do not appear to have invested major resources into managing this species.

Globe thistle was observed resprouting from its base after being mown to the ground.

Routine mowing appears to limit globe thistle occurrence. Manicured lawns mowed several times per year seem to reduce the frequency of this species.

Herbicide appears effective at killing globe thistle, but types and concentration details are lacking from site visits (Dreisilker pers. comm.)

Identify types of control methods and time-term required:

Rationale:

Sources of information:

Total Possible: 7

Total: 5

Total for 4 sections Possible: 79

Total for 4 sections: 41

References:

Carlson, M. 2008. Invasiveness Ranking System for Non-Native Plants of Alaska. USDA. Available at: <http://www.fs.usda.gov/Internet/FSE_DOCUMENTS/fsbdev2_037575.pdf>

Dreisilker, K. 2012. Investigation and control of new invasive plant species at the Morton Arboretum. Final report to northeast Illinois invasive plant partnership. The Morton Arboretum. http://niipp.net/wp-content/uploads/2011/01/Morton_Arboretum_Final_Report_2012.pdf

USDA PLANTS database

FNO 1993. Flora of North America Editorial Committee, eds. 1993+. Flora of North America North of Mexico. 16+ vols. New York and Oxford.

ILPIN. (Illinois Plant Information Network) <http://www.fs.fed.us/ne/delaware/ilpin/1143.co>

Moracova, L. et al. 2010. Reproductive characteristics of neophytes in the Czech Republic: traits of invasive and non-invasive species. Preslia 82:365-390

Petrik, P., et al. 2009. Combining numerical and traditional approaches to classify Echinops sphaerocephalus invaded communities in the Czech Republic. 3, pp.253–264.

Notes

A species of roadsides and fields (ILPIN), but may also have effects in natural areas (Petrik et al. 2009)

Score Interpretation

While different users will have different concepts of what constitutes various levels of invasiveness (e.g., what is “highly invasive” vs. “moderately invasive” may differ among management agencies), we divided the ranks into six blocks in Appendix A. We consider species with scores ≥80 as “Extremely Invasive” and species with scores 70–79 as “Highly Invasive;” both of these groups are composed of species estimated to be very threaten­ing to Alberta. Species with scores of 60–69 as “Moderately Invasive” and scores of 50–59 represent “Modestly Invasive” species; both of these groups still pose significant risks to ecosystems. Species with scores of 40–49 are “Weakly Invasive”, and <40 are considered “Very Weakly Invasive.” These last two groups generally have not been shown to significantly alter ecosystem processes and communities elsewhere and probably do not require as much attention as the other species.

**Alberta non-native plant invasiveness ranking form**

(Adapted from Carlson et al. 2008)

| Scientific name: | *Ulex europaeus* |
| --- | --- |
| Common name: | gorse |
| Assessor: | Shauna-Lee Chai |
| Reviewers: | David Clements |
| Date: | October 4, 2013 |

Outcome score:

1. Climatic Comparison

This species is present or may potentially establish in the following natural regions:

|  | Collected in Alberta regions | CLIMEX similarity in  1975 | CLIMEX similarity in  2050 |
| --- | --- | --- | --- |
| Boreal | No | 0.677 | 0.735 |
| Parkland | No | 0.748 | 0.794 |
| Foothills | No | 0.776 | 0.821 |
| Grassland | No | 0.742 | 0.763 |
| Rocky Mountains | No | 0.703 | 0.746 |
| Shield | No | 0.591 | 0.661 |

(0.7 is the climate suitability threshold)

1. Invasiveness Ranking Total (Total answered^1^ points possible) Total score

| 1. Ecological impact | 40(40) | 31 |
| --- | --- | --- |
| 1. Biological characteristics and dispersal ability | 25(25) | 14 |
| 1. Ecological amplitude and distribution | 25(25) | 11 |
| 1. Feasibility of control | 10(10) | 9 |
| Outcome score | 100(100)^b^ | ^a^65 |
| Relative maximum score^2^ | 65 | *‘Moderately invasive’* |

^1^For questions answered “unknown” do not include point value for the question in parentheses for “Total answered points possible.”

^2^Calculated as a/b x 100.

1. Climatic Comparison:
   1. Has this species ever been collected or documented in Alberta?

__Yes – continue to 1.2

x No – continue to 2.1

Which natural region has it been collected or documented (see inset map)? Proceed to section B. Invasiveness Ranking.

__Boreal

__Rockies

__Grassland

__Foothills

__Parkland

__Shield

Documentation:

Sources of information: ANPC Rogues gallery, ACIMS, PLANTS database, GBIF

2.1 Is there a 70 percent or higher similarity (based on CLIMEX climate matching) between climates anywhere the species currently occurs and

a. Boreal – not in 1975, but in 2050

b. Rockies - Yes

c. Grassland - Yes

d. Foothills - Yes

e. Parkland - Yes

f. Shield - no

-If “no” is answered for all regions, reject species from consideration

Documentation:

Sources of information:

1. Invasiveness Ranking
2. Ecological Impact
   1. Impact on Natural Ecosystem Processes
3. No perceivable impact on ecosystem processes 0
4. Has the potential to influence ecosystem processes to a minor degree

(e.g., has a perceivable but mild influence on soil nutrient availability) 3

1. Has the potential to cause significant alteration of ecosystem processes (e.g., increases sedimentation rates along streams or coastlines, reduces open water

that are important to waterfowl) 7

1. May cause major, possibly irreversible, alteration or disruption of ecosystem processes (e.g., the species alters geomorphology; hydrology; or affects fire frequency, altering community composition; species fixes substantial levels of nitrogen in the soil making soil unlikely to support certain native plants or more likely to favor non-native species) 10

u. Unknown

Score: 10

Documentation: Extremely competitive, displaces native plants, alters soils by fixing nitrogen and making soils acidic. Its optimal pH is 4.5-5 (Grubb et al. 1969). It extracts plant nutrients-Ca, Mg, Na which alters nutrient dynamics and can impoverish soil. Its oily seeds and foliage, which accumulate as litter, are highly flammable and it is an extreme fire hazard due to abundant dead material. Soil is often bare between plants increasing the likelihood of erosion. Grows spines and is in dense thickets and excludes grazing animals, reducing pasture quality. Can also interfere with economically important conifer seedling growth in forests (ISSG 2013). In New Zealand a fire spread rapidly through a gorse understorey destroying 1000 ha of forest plantation. Similar risks exist in BC’s coastal areas (Zielke et al 1992).

Identify ecosystem processes impacted: fire, nutrient dynamics

Rational:

Sources of information: ISSG 2013

- 1. Impact on Natural Community Structure

1. No perceived impact; establishes in an existing layer without influencing its

structure 0

1. Has the potential to influences structure in one layer (e.g., changes the density

of one layer) 3

1. Has the potential to cause significant impact in at least one layer (e.g., creation

of a new layer or elimination of an existing layer) 7

1. Likely to cause major alteration of structure (e.g., covers canopy, eradicating

most or all layers below) 10

1. Unknown

Score:7

Documentation: grows densely and excludes other species due to heavy leaf litter, acidifying soil and nutrient competition (ISSG). Grubb et al. (1969) observed in Great Britain that only a few woody species could grow under a canopy of U. europaeus. Evergreen habit and canopy architecture reduces light to other species.

Identify type of impact or alteration:

Rational:

Sources of information:

- 1. Impact on Natural Community Composition

1. No perceived impact; causes no apparent change in native populations 0
2. Has the potential to influences community composition (e.g., reduces the

number of individuals in one or more native species in the community) 3

1. Has the potential to significantly alters community composition (e.g., produces

a significant reduction in the population size of one or more native species in

the community) 7

1. Likely to cause major alteration in community composition (e.g., results in the extirpation of one or several native species, reducing biodiversity or change the community composition towards species exotic to the natural community) 10

u. Unknown

Score:7

Documentation: as above. In BC’s west coast, rare species occupy gorse range, particularly in Garry oak ecosystems (Erickson 1993). Potentially excludes rare species such as Howell’s triteleia (Triteleia howellii Greene), golden paintbrush, (Castilleja levisecta Greenm) or deltoid balsamroot [Balsamorhiza del- toidea (Benth.) A. Gray] associated with the threatened Garry oak (Quercus garryana Douglas) ecosystem in British Columbia (Erickson 1993).

Identify type of impact or alteration:

Rational:

Sources of information:

- 1. Impact on higher trophic levels (cumulative impact of this species on the animals,

fungi, microbes, and other organisms in the community it invades)

1. Negligible perceived impact 0
2. Has the potential to cause minor alteration 3
3. Has the potential to cause moderate alteration (minor reduction in

nesting/foraging sites, reduction in habitat connectivity, interference with

native pollinators, injurious components such as spines, toxins) 7

1. Likely to cause severe alteration of higher trophic populations (extirpation or endangerment of an existing native species/population, or significant reduction

in nesting or foraging sites) 10

u. Unknown

Score: 7

Documentation: Not generally palatable due to spines (ISSG), although some ungulates such as sheep or goats may forage on it (Radcliffe 1985).

Identify type of impact or alteration:

Rational:

Sources of information:

Total Possible: 40

Total: 31

1. Biological Characteristics and Dispersal Ability
   1. Mode of reproduction
2. Not aggressive reproduction (few [0-10] seeds per plant and no

vegetative reproduction) 0

1. Somewhat aggressive (reproduces only by seeds (11-1,000/m2) 1
2. Moderately aggressive (reproduces vegetatively and/or by a moderate

amount of seed, <1,000/m2) 2

1. Highly aggressive reproduction (extensive vegetative spread and/or

many seeded, >1,000/m2) 3

u. Unknown

Score: 2

Documentation: ISSG

Describe key reproductive characteristics (including seeds per plant):500-600 seeds/m^2^, longevity of seed in soil, evergreen habit (Clements et al 2001). Vegetative reproduction is possible by creeping roots or fragments especially after disturbance/cutting or fire (Hoshovsky 1986); low stature plants may spread via lateral vegetative growth over many hectares after repeated mowing (Dennehy et al 2011).

Rational:

Sources of information: Johnson 2001

- 1. Innate potential for long-distance dispersal (bird dispersal, sticks to animal hair, buoyant fruits, wind-dispersal)

1. Does not occur (no long-distance dispersal mechanisms) 0
2. Infrequent or inefficient long-distance dispersal (occurs occasionally

despite lack of adaptations) 2

1. Numerous opportunities for long-distance dispersal (species has

adaptations such as pappus, hooked fruit-coats, etc.) 3

1. Unknown

Score:2

Documentation: flattened winged pod present. Occasional long distance dispersal is water, wildlife. Long distance dispersal is infrequent (Zouhar 2005). Seeds are 6 mg and 2 mm long with hard water resistant coats and elaiosomes (Clements et al 2001)

Identify dispersal mechanisms:

Rational:

Sources of information:

- 1. Potential to be spread by human activities (both directly and indirectly – possible mechanisms include: commercial sales, use as forage/revegetation, spread along highways, transport on boats, contamination, etc.)

1. Does not occur 0
2. Low (human dispersal is infrequent or inefficient) 1
3. Moderate (human dispersal occurs) 2
4. High (there are numerous opportunities for dispersal to new areas) 3

u. Unknown

Score: 2

Documentation: spread of seeds by vehicles along roads, logging and agricultural equipment readily occurs (Clements et al 2001).

Identify dispersal mechanisms:

Rational:

Sources of information:

- 1. Allelopathic

1. no 0
2. yes 2
3. unknown

Score:0

Documentation:

There are no reports of allelopathic activity in U. europaeus.

Rational:

Sources of information: ISSG

- 1. Competitive ability

a. Poor competitor for limiting factors 0

b. Moderately competitive for limiting factors 1

c. Highly competitive for limiting factors and/or nitrogen fixing ability 3

u. Unknown

Score:3

Documentation: Grows even in poor soils

Evidence of competitive ability:

Rational:

Sources of information: ISSG

- 1. Forms dense thickets, climbing or smothering growth habit, or otherwise taller than the surrounding vegetation

1. No 0
2. Forms dense thickets 1
3. Has climbing or smothering growth habit, or otherwise taller than the surrounding

vegetation 2

u. Unknown

Score:2

Documentation: shrub usually up to 4.8 m tall. Dense, impenetrable thickets (Zouhar 2005). After 25-30 yr of dominating a site, other species may establish as gorse declines (Lee et al 1986)

Describe growth form: erect in British Columbia.

Rational:

Sources of information:

- 1. Germination requirements

1. Requires open soil and disturbance to germinate 0
2. Can germinate in vegetated areas but in a narrow range or in special conditions 2
3. Can germinate in existing vegetation in a wide range of conditions 3

u. Unknown

Score:2

Documentation: Increased germination is observed after disturbance in open conditions but does U. europaeus does not readily germinate under heavy vegetative cover (Richardson and Hill 1998).

Describe germination requirements: A large range of germination percentages are observed under different conditions, because of the hard seed coat which often renders seeds dormant.

Rational:

Sources of information: Zouhar 2005

- 1. Other species in the genus invasive in Alberta or elsewhere

1. No 0
2. Yes 3

u. Unknown

Score:0

Documentation: Associated species include in the Atlantic heathlands in France and Spain include dwarf gorse (U. minor), but no record of this species being invasive was found (Zouhar 2005).

Species:

Sources of information:

2.9 Aquatic, wetland, or riparian species

a. Not invasive in wetland communities 0

b. Invasive in riparian communities 1

c. Invasive in wetland communities 3

u. Unknown

Score:1

Documentation: capable of invading riverbeds and riparian areas in New Zealand (Zouhar 2005)

Describe type of habitat:

Rational:

Sources of information:

Total Possible:25

Total:14

1. Distribution
   1. Is the species highly domesticated or a weed of agriculture
2. No 0
3. Is occasionally an agricultural pest 2
4. Has been grown deliberately, bred, or is known as a significant agricultural pest 4
5. Unknown

Score:2

Documentation: Introduced as a hedge plant to contain livestock and for ornamental purposes and spread causing problems for livestock in some areas (Krause et al 1988, ISSG, Zouhar 2005)

Identify reason for selection, or evidence of weedy history:

Rational:

Sources of information:

- 1. Known level of ecological impact in natural areas

1. Not known to cause impact in any other natural area 0
2. Known to cause impacts in natural areas, but in dissimilar habitats and

climate zones than exist in regions of Alberta 1

1. Known to cause low impact in natural areas in similar habitats and climate

zones to those present in Alberta 3

1. Known to cause moderate impact in natural areas in similar habitat and

climate zones 4

1. Known to cause high impact in natural areas in similar habitat and climate

zones 6

u. Unknown

Score:1

Documentation: Invasive effects of the species is best recorded in New Zealand. Impacts recorded in Hinewai Reserve in New Zealand (Wilson 1990, 1994). In BC’s west coast, rare species occupy gorse range within Garry oak ecosystems (Erickson 1993). Potentially excludes rare species such as Howell’s triteleia (Triteleia howellii Greene), golden paintbrush, (Castilleja levisecta Greenm) or deltoid balsamroot [Balsamorhiza del- toidea (Benth.) A. Gray] associated with the threatened Garry oak (Quercus garryana Douglas) ecosystem in British Columbia (Erickson 1993).

Identify type of habitat and states or provinces where it occurs: forest

Sources of information:

- 1. Role of anthropogenic and natural disturbance in establishment

1. Requires anthropogenic disturbances to establish 0
2. May occasionally establish in undisturbed areas but can readily establish in

areas with natural disturbances 3

1. Can establish independent of any known natural or anthropogenic disturbances 5

u. Unknown

Score: 0

Documentation: In Canada, it is only found in BC. Found in areas with degraded soil and disturbed sites such as roadsides, pasture lands, cleared forests, gravelly floodplains. Likewise establishment throughout its invaded range has been primarily through anthropogenic disturbance such as soil disturbance or deliberate planting (Richardson and Hill 1988).

Identify type of disturbance:

Rationale:

Sources of information: ISSG

- 1. Current global distribution

1. Occurs in one or two continents or regions (e.g., Mediterranean region) 0
2. Extends over three or more continents 3
3. Extends over three or more continents, including successful introductions in

arctic or subarctic regions 5

u. Unknown

Score:3

Documentation: Europe, Canada, South America, South Africa. The geographical distribution is controlled by temperature. It thrives in maritime climates similar to its native Mediterranean Europe, incl New Zealand)(Lee et al. 1986). It cannot tolerate arid climates or continental regions with extreme cold or heat and thus tends to occur in coastal areas. Short days inhibit maturation and prevents thorn formation and flowering. It will grow on most soil types (ISSG). Can occur in same habitats as *Cytisus scoparius* (scotch broom) but on drier sites. It invades gradually and often goes unnoticed. Climate warming favours spread (Zielke et al 1992).

Describe distribution: From Virginia to Massachusetts and noxious in BC. Dry and degraded sites (Clements et al 2001).

Rational:

Sources of information:

- 1. Extent of the species Canada range and/or occurrence of formal state or provincial listing

1. 0-5 percent of the states/provinces 0
2. 6-20 percent of the states/provinces 2
3. 21-50 percent, and/or state/province listed as a problem weed

(e.g., “Noxious,” or “Invasive”) in 1 state or Canadian province 4

1. Greater than 50 percent, and/or identified as “Noxious” in 2 or more states or

Canadian provinces 5

u. Unknown

Score:5

Documentation: Gorse is listed as a noxious weed in Washington, Oregon, California, Hawaii, and British Columbia. Identify provinces invaded: BC

Rational:

Sources of information:

Total possible:25

Total:11

1. Feasibility of Control
   1. Seed banks
2. Seeds remain viable in the soil for less than 3 years 0
3. Seeds remain viable in the soil for between 3 and 5 years 2
4. Seeds remain viable in the soil for 5 years and more 3

u. Unknown

Score:3

Documentation:

Identify longevity of seed bank: Seed viability is variable by location. Up to 30 yrs has been reported.

Rational:

Sources of information: ISSG

- 1. Vegetative regeneration

1. No resprouting following removal of aboveground growth 0
2. Resprouting from ground-level meristems 1
3. Resprouting from extensive underground system 2
4. Any plant part is a viable propagule 3

u. Unknown

Score:2

Documentation:

Describe vegetative response: Vegetative reproduction is possible by creeping roots or fragments especially after disturbance/cutting or fire (Hoshovsky 1986, Dennehy et al 2011)

Rational:

Sources of information:

- 1. Level of effort required

1. Management is not required (e.g., species does not persist without repeated anthropogenic disturbance) 0
2. Management is relatively easy and inexpensive; requires a minor investment in human and financial resources 2
3. Management requires a major short-term investment of human and financial resources, or a moderate long-term investment 3
4. Management requires a major, long-term investment of human and financial resources 4

u. Unknown

Score:4

Documentation: Annual costs for control in new Zealand are $17.8 million and $8 million in the agriculture and forestry sectors respectively (Sandrey 1985). Once established it is more difficult to eradicate than scotch broom (Zielke et al 1992, Clements et al 2001).

Identify types of control methods and time-term required:

Rational:

Sources of information:

Total Possible: 10

Total: 9

Total for 4 sections Possible: 100

Total for 4 sections: 65

Score Interpretation (Carlson et al. 2008):

While different users will have different concepts of what constitutes various levels of invasiveness (e.g., what is “highly invasive” vs. “moderately invasive” may differ among management agencies), we divided the ranks into six blocks in Appendix A. We consider species with scores ≥80 as “Extremely Invasive” and species with scores 70–79 as “Highly Invasive;” both of these groups are composed of species estimated to be very threatening to Alaska. Species with scores of 60–69 as “Moderately Invasive” and scores of 50–59 represent “Modestly Invasive” species; both of these groups still pose significant risks to ecosystems. Species with scores of 40–49 are “Weakly Invasive”, and <40 are considered “Very Weakly Invasive.” These last two groups generally have not been shown to significantly alter ecosystem processes and communities elsewhere and probably do not require as much attention as the other species.

Thus gorse, Ulex europaeus, with a combined score for all 4 sections of 65 out of a possible 100 is characterized as “moderately invasive” (Carlson et al 2008).

Notes: On the world’s 100 worst list

References:

ANPC Rogues gallery http://www.anpc.ab.ca/wiki/index.php/Main_Page, ACIMS <http://albertaparks.ca/albertaparksca/management-land-use/alberta-conservation-information-management-system-(acims).aspx> , PLANTS database http://plants.usda.gov/java/, GBIF <http://www.gbif.pt/>, ISSG http://www.issg.org/

Carlson, M. L., Lapina, I. V., Shephard, M., Conn, J. S., Densmore, R., Spencer, P., Heys, J., Riley, J. and Nielsen, J. 2008. Invasiveness Ranking System for Non-Native Plants of Alaska. USDA. Available at: <http://www.fs.usda.gov/Internet/FSE_DOCUMENTS/fsbdev2_037575.pdf>

Clements, D. R., Peterson D.J., and Prasad, R. 2001. The biology of Canadian weeds. 112. Ulex europaeus L." Canadian Journal of Plant Science 81: 325-337.

Dennehy C., Alverson, E.R., Anderson, H.E., Clements, D.R., Gilbert, E.R., and Kaye, T.N. 2011. Management strategies for invasive plants in Pacific Northwest prairies, savannas, and oak woodlands. Northwest Sci. 85: 329-351.

Erickson, W. 1993. Garry Oak ecosystems. Ministry of Environment, Lands and Parks, Victoria, BC 4pp.

Grubb, P. J., Green, H. E. and Merrifield, R. C. J. 1969. The ecology of chalk heath: its relevance to the calcicole-calcifuge and soil acidification problems. J. Ecol. 57: 175–212

Hoshovsky, M. 1986. Element Stewardship Abstract for Ulex europaeus. Unpublished. The Nature Conservancy, California Field Office, 785 Market St., 3rd floor, San Francisco, CA. 25 pp. (http://tncweeds.ucdavis.edu/esadocs/ulexeuro.html)

Krause, M. A., Beck, A. C. and Dent, J. B. 1988. Control of gorse in hill country: an economic assessment of chemical and biological methods. Agric. Systems 26: 35–49.

Johnson, P. N. 2001. Vegetation recovery after fire on a southern New Zealand peatland. New Zealand Journal of Botany. 39(2): 251-267.

Lee, W. G., Allen, R. B. and Johnson, P. N. 1986. Succession and dynamics of gorse (Ulex europaeus L.) communities in the Dunedin Ecological District, South Island, New Zealand. N.Z. J. Bot. 24: 279–292.

Radcliffe, J. E. 1985. Grazing management of goats and sheep for gorse control. N.Z. J. Exp. Agric. 13: 181–190.

Richardson, R. G., Hill, R. L. 1998. The biology of Australian weeds. 34. Ulex europaeus L. Plant Prot. Q. 13(2): 46-58.

Sandrey, R. 1985. Biological control of gorse: an ex-ante evalua- tion. Agricultural Economics Research Unit Research Report No. 172. Lincoln College, Canterbury, New Zealand. 96 pp.

Wilson, HD. 1990. Gorse on Hinewai Reserve. Cantebury Botanical Society Journal 24: 45-47.

Wilson, HD 1994. Regeneration of native forest on Hinewai Reserve, Banks Peninsula. N.Z.J Bot. 32: 373-383.

Zielke, K., Boateng, J., Caldicott, N. and Williams, H. 1992. Broom and Gorse: A Forestry Perspective Problem Analysis. British Columbia Ministry of Forests, Queen’s Printer, Victoria, BC. 20 pp.

Zouhar, Kris. 2005. Ulex europaeus. In: Fire Effects Information System, [Online]. U.S. Department of Agriculture, Forest Service, Rocky Mountain Research Station, Fire Sciences Laboratory (Producer). Available: http://www.fs.fed.us/database/feis/ [2013, October 7].

http://www.fs.fed.us/database/feis/plants/shrub/uleeur/all.html

**Alberta non-native plant invasiveness ranking form**

(Adapted from Carlson et al. 2008)

| Scientific name: | *Taeniatherum caput-medusae/* *Elymus caput-medusae*  In USA, subspecies is*: Taeniatherum caput-medusae ssp. asperum* |
| --- | --- |
| Common name: | Medusahead |
| Assessor: | Shauna-Lee Chai |
| Reviewer: | Kirk W. Davies |
| Date: | November 6, 2013 |

Outcome score:

1. Climatic Comparison

This species is present or may potentially establish in the following natural regions:

|  | Collected in Alberta regions | CLIMEX similarity in 1975 | CLIMEX similarity in 2050 |
| --- | --- | --- | --- |
| Boreal | No | 0.730 | 0.798 |
| Parkland | No | 0.821 | 0.872 |
| Foothills | No | 0.810 | 0.841 |
| Grassland | No | 0.875 | 0.900 |
| Rocky Mountains | No | 0.687 | 0.712 |
| Shield | No | 0.637 | 0.724 |

1. Invasiveness Ranking Total (Total answered^1^ points possible) Total score

| 1. Ecological impact | 40(40) | 37 |
| --- | --- | --- |
| 1. Biological characteristic and dispersal ability | 25(25) | 17 |
| 1. Ecological amplitude and distribution | 25(25) | 21 |
| 1. Feasibility of control | 10(10) | 3 |
| Outcome score | 100(100)^b^ | ^a^78 |
| Relative maximum score^2^ | 78 | *Highly Invasive* |

^1^For questions answered “unknown” do not include point value for the question in parentheses for “Total answered points possible.”

^2^Calculated as a/b x 100.

1. Climatic Comparison:
   1. Has this species ever been collected or documented in Alberta?

__Yes – continue to 1.2

x No – continue to 2.1

Which natural region has it been collected or documented (see inset map)? Proceed to section B. Invasiveness Ranking.

__Boreal

__Rockies

__Grassland

__Foothills

__Parkland

__Shield

Documentation:

Sources of information: ANPC Rogues gallery, ACIMS, PLANTS database, GBIF

2.1 Is there a 70 percent or higher similarity (based on CLIMEX climate matching) between climates anywhere the species currently occurs and

a. Boreal -Yes

b. Rockies – Not in 1975, but in 2050

c. Grassland - Yes

d. Foothills - Yes

e. Parkland -Yes

f. Shield - Not in 1975, but in 2050

-If “no” is answered for all regions, reject species from consideration

Documentation:

Sources of information:

1. Invasiveness Ranking
2. Ecological Impact
   1. Impact on Natural Ecosystem Processes
3. No perceivable impact on ecosystem processes 0
4. Has the potential to influence ecosystem processes to a minor degree

(e.g., has a perceivable but mild influence on soil nutrient availability) 3

1. Has the potential to cause significant alteration of ecosystem processes (e.g., increases sedimentation rates along streams or coastlines, reduces open water

that are important to waterfowl) 7

1. May cause major, possibly irreversible, alteration or disruption of ecosystem processes (e.g., the species alters geomorphology; hydrology; or affects fire frequency, altering community composition; species fixes substantial levels of nitrogen in the soil making soil unlikely to support certain native plants or more likely to favor non-native species) 10

u. Unknown

Score: 10

Documentation: High silica content makes it hard to decompose and standing dead biomass is extremely flammable and creates enormous fuel load (Swenson et al 1964). Medusahead has substantially increased frequency of fire in the western United States (Knapp 1998, Davies and Svejcar 2008).

Identify ecosystem processes impacted:

Rational:

Sources of information:

- 1. Impact on Natural Community Structure

1. No perceived impact; establishes in an existing layer without influencing its

structure 0

1. Has the potential to influences structure in one layer (e.g., changes the density

of one layer) 3

1. Has the potential to cause significant impact in at least one layer (e.g., creation

of a new layer or elimination of an existing layer) 7

1. Likely to cause major alteration of structure (e.g., covers canopy, eradicating

most or all layers below) 10

1. Unknown

Score:10

Documentation: Frequent fires destroy the shrub component of the plant community, and potentially part of the bunchgrass community (Miller et al 1999). It also alters the herbaceous structure by making a more continuous homogeneous medusahead and thatch layer. Whereas native communities have variable height from different species and in drier regions bare ground between herbacoues vegetation (Davies and Svejcar 2008, Davies 2011).

Identify type of impact or alteration:

Rational:

Sources of information:

- 1. Impact on Natural Community Composition

1. No perceived impact; causes no apparent change in native populations 0
2. Has the potential to influence community composition (e.g., reduces the

number of individuals in one or more native species in the community) 3

1. Has the potential to significantly alters community composition (e.g., produces

a significant reduction in the population size of one or more native species in

the community) 7

1. Likely to cause major alteration in community composition (e.g., results in the extirpation of one or several native species, reducing biodiversity or change the community composition towards species exotic to the natural community) 10

u. Unknown

Score:10

Documentation: Medusahead-dominated areas have very low species diversity and low value for wildlife habitat (Miller et al 1999). It migrates rapidly, is vigorously competitive and of low forage quality (Archer 2001). The fires it encourages reduces species diversity (Zimmerman n.d.). Davies (2011) found that increasing medushead abundance greatly decreased native plant abundance and biodiversity.

Identify type of impact or alteration:

Rational:

Sources of information:

- 1. Impact on higher trophic levels (cumulative impact of this species on the animals,

fungi, microbes, and other organisms in the community it invades)

1. Negligible perceived impact 0
2. Has the potential to cause minor alteration 3
3. Has the potential to cause moderate alteration (minor reduction in

nesting/foraging sites, reduction in habitat connectivity, interference with

native pollinators, injurious components such as spines, toxins) 7

1. Likely to cause severe alteration of higher trophic populations (extirpation or endangerment of an existing native species/population, or significant reduction

in nesting or foraging sites) 10

u. Unknown

Score:7

Documentation: Medusahead is a major concern to the range livestock industry because it can suppress desirable vegetation. It is unpalatable to livestock (Zimmerman, n.d.). When dry, dead vegetation decomposes slowly and forms a persistent dense litter on the soil surface. As the plant matures it develops long barbed awns that can cause injury to the eyes, noses, and mouths of grazing animals (Bovey et al 1960). It is not preferred by wildlife as forage (Savage et al 1969). Increased fire frequency with medusahead invasion has resulted in the loss of shrub habitat for shrub obligate wildlife species, such as sage-grouse (Davies and Svejcar 2008).

Identify type of impact or alteration:

Rational:

Sources of information:

Total Possible:40

Total:37

1. Biological Characteristics and Dispersal Ability
   1. Mode of reproduction
2. Not aggressive reproduction (few [0-10] seeds per plant and no

vegetative reproduction) 0

1. Somewhat aggressive (reproduces only by seeds (11-1,000/m2) 1
2. Moderately aggressive (reproduces vegetatively and/or by a moderate

amount of seed, <1,000/m2) 2

1. Highly aggressive reproduction (extensive vegetative spread and/or

many seeded, >1,000/m2) 3

u. Unknown

Score:3

Documentation: Plants produce up to 6,000 seeds/ft^2^ of soil (Major et al 1960)

Describe key reproductive characteristics (including seeds per plant):

Rational:

Sources of information:

- 1. Innate potential for long-distance dispersal (bird dispersal, sticks to animal hair, buoyant fruits, wind-dispersal)

1. Does not occur (no long-distance dispersal mechanisms) 0
2. Infrequent or inefficient long-distance dispersal (occurs occasionally

despite lack of adaptations) 2

1. Numerous opportunities for long-distance dispersal (species has

adaptations such as pappus, hooked fruit-coats, etc.) 3

1. Unknown

Score:3

Documentation: Animals, wind, and water disperse the seed, and spread is rapid. A rough awn aids in animal dispersal of seed (Furbush 1953). ). Medusahead is also likely to be spread long distance by animals because it often grows in clay soil that adheres, with embedded seeds, to animal’s feet when moist (Davies et al 2013).

Identify dispersal mechanisms:

Rational:

Sources of information:

- 1. Potential to be spread by human activities (both directly and indirectly – possible mechanisms include: commercial sales, use as forage/revegetation, spread along highways, transport on boats, contamination, etc.)

1. Does not occur 0
2. Low (human dispersal is infrequent or inefficient) 1
3. Moderate (human dispersal occurs) 2
4. High (there are numerous opportunities for dispersal to new areas) 3

u. Unknown

Score: 3

Documentation: Humans help spread this species by grazing and poor rangeland health. Vehicles are one the major dispersal vectors of medusahead (Davies et al. 2013).

Identify dispersal mechanisms:

Rational:

Sources of information:

- 1. Allelopathic

1. no 0
2. yes 2
3. unknown

Score:0

Documentation:

Describe effect on adjacent plants:

Rational:

Sources of information:

- 1. Competitive ability

a. Poor competitor for limiting factors 0

b. Moderately competitive for limiting factors 1

c. Highly competitive for limiting factors and/or nitrogen fixing ability 3

u. Unknown

Score:3

Documentation: Clausnitzer DW, Borman MM, Johnson DE (1999) Competition between Elymus elymoides and Taeniatherum caput-medusae. Weed Sci. 47: 720-728

Young K, Mangold J (2008) Medusahead (Taeniatherum caput-medusae) outperforms squirreltail (Elymus elymoides) through interference and growth rate. Invasive Plant Sci Manage 1: 73-8

Harris GA, Wilson AM (1970) Competition for moisture among seedlings of annual and perennial grasses as influenced by root elongation at low temperatures. Ecology 51: 530-534

Hironaka M, Sindelar BW (1975) Growth characteristics of squirreltail seedlings in competition with medusahead. J Range Manage 28: 283-285

Leffler AJ, James JJ, Monaco TA (2013) Temperature and functional traits influence differences in nitrogen uptake capacity between native and invasive grasses. Oecologia 171: 51-60

Evidence of competitive ability:

Rational:

Sources of information:

- 1. Forms dense thickets, climbing or smothering growth habit, or otherwise taller than the surrounding vegetation

1. No 0
2. Forms dense thickets 1
3. Has climbing or smothering growth habit, or otherwise taller than the surrounding

vegetation 2

u. Unknown

Score:1

Documentation: grows densely- 1,500 to 2,000 plants/ft2 (Sharp et al 1969). The formation of thick, persistent, thatch layers with medusahead inhibit growth of rival species (Davies and Svejcar 2008)

Describe growth form:

Rational:

Sources of information:

- 1. Germination requirements

1. Requires open soil and disturbance to germinate 0
2. Can germinate in vegetated areas but in a narrow range or in special conditions 2
3. Can germinate in existing vegetation in a wide range of conditions 3

u. Unknown

Score:0

Documentation:

Describe germination requirements:

Rational:

Sources of information:

- 1. Other species in the genus invasive in Alberta or elsewhere

1. No 0
2. Yes 3

u. Unknown

Score:3

Documentation: E.G. *Elymus repens* –couch grass

Species:

Sources of information:

2.9 Aquatic, wetland, or riparian species

a. Not invasive in wetland communities 0

b. Invasive in riparian communities 1

c. Invasive in wetland communities 3

u. Unknown

Score:1

Documentation: Archer, Amy J. 2001. Taeniatherum caput-medusae. In: Fire Effects Information System, [Online]. U.S. Department of Agriculture, Forest Service, Rocky Mountain Research Station, Fire Sciences Laboratory (Producer). Available: http://www.fs.fed.us/database/feis/ [2013, November 7].

Describe type of habitat:

Rational:

Sources of information:

Total Possible:25

Total:17

1. Distribution
   1. Is the species highly domesticated or a weed of agriculture
2. No 0
3. Is occasionally an agricultural pest 2
4. Has been grown deliberately, bred, or is known as a significant agricultural pest 4
5. Unknown

Score:4

Documentation: Medusahead invasion can reduce the grazing capacity of rangelands by at least 50-80% (Hironaka 1961)

Identify reason for selection, or evidence of weedy history:

Rational:

Sources of information:

- 1. Known level of ecological impact in natural areas

1. Not known to cause impact in any other natural area 0
2. Known to cause impacts in natural areas, but in dissimilar habitats and

climate zones than exist in regions of Alberta 1

1. Known to cause low impact in natural areas in similar habitats and climate

zones to those present in Alberta 3

1. Known to cause moderate impact in natural areas in similar habitat and

climate zones 4

1. Known to cause high impact in natural areas in similar habitat and climate

zones 6

u. Unknown

Score:6

Documentation: Medusahead maintain its dominance on sites where native vegetation has been eliminated or severely reduced by overgrazing, cultivation, or frequent fires. It has invaded fields, dry roadsides, and disturbed sagebrush slopes in British Columbia, Washington, Idaho, Oregon, and California (Cronquist et al. 1977). ). The NRCS plant database (http://plants.usda.gov/core/profile?symbol=TACA8) shows medusahead has invaded areas in Washington, Oregon, California, Idaho, Nevada, Utah, Montana, Pennsylvania, New York, and Connecticut. Invades ecologically disturbed areas.

Identify type of habitat and states or provinces where it occurs:

Sources of information:

- 1. Role of anthropogenic and natural disturbance in establishment

1. Requires anthropogenic disturbances to establish 0
2. May occasionally establish in undisturbed areas but can readily establish in

areas with natural disturbances 3

1. Can establish independent of any known natural or anthropogenic disturbances 5

u. Unknown

Score:3

Documentation: Davies 2008

Identify type of disturbance:fire

Rationale:

Sources of information:

- 1. Current global distribution

1. Occurs in one or two continents or regions (e.g., Mediterranean region) 0
2. Extends over three or more continents 3
3. Extends over three or more continents, including successful introductions in

arctic or subarctic regions 5

u. Unknown

Score:3

Documentation: North and South America, Africa, Europe

Describe distribution: native to Mediterranean region of Europe. Medusahead and cheatgrass (Bromus tectorum), another invasive non-native species, overlap in distribution and habitat requirements. Each can replace other herbaceous vegetation and share dominance with the other.

Rational:

Sources of information:

- 1. Extent of the species Canada range and/or occurrence of formal state or provincial listing

1. 0-5 percent of the states/provinces 0
2. 6-20 percent of the states/provinces 2
3. 21-50 percent, and/or state/province listed as a problem weed

(e.g., “Noxious,” or “Invasive”) in 1 state or Canadian province 4

1. Greater than 50 percent, and/or identified as “Noxious” in 2 or more states or

Canadian provinces 5

u. Unknown

Score:5

Documentation: Noxious in California, Colorado, Nevada, Oregon and Utah (USDA Plants)

Identify provinces invaded:

Rational:

Sources of information:

Total possible:25

Total:21

1. Feasibility of Control
   1. Seed banks
2. Seeds remain viable in the soil for less than 3 years 0
3. Seeds remain viable in the soil for between 3 and 5 years 2
4. Seeds remain viable in the soil for 5 years and more 3

u. Unknown

Score:0

Documentation: Short-lived seed bank-viable for at least 1 year (Sharp et al 1957)

Identify longevity of seed bank

Rational:

Sources of information:

- 1. Vegetative regeneration

1. No resprouting following removal of aboveground growth 0
2. Resprouting from ground-level meristems 1
3. Resprouting from extensive underground system 2
4. Any plant part is a viable propagule 3

u. Unknown

Score:0

Documentation:

Describe vegetative response:

Rational:

Sources of information:

- 1. Level of effort required

1. Management is not required (e.g., species does not persist without repeated anthropogenic disturbance) 0
2. Management is relatively easy and inexpensive; requires a minor investment in human and financial resources 2
3. Management requires a major short-term investment of human and financial resources, or a moderate long-term investment 3
4. Management requires a major, long-term investment of human and financial resources 4

u. Unknown

Score:3

Documentation: A combination of treatments including grazing, burning, mechanical manipulation, herbicide such as atrazine, or glyphosate, pre-emergent such as imazapic and/or reseeding are generally necessary to reduce established stands of medusahead (Christensen et al. 1974, Monaco et al. 2005)

Identify types of control methods and time-term required:

Rational:

Sources of information:

Total Possible: 10

Total: 3

Total for 4 sections Possible: 100

Total for 4 sections: 78

References:

Archer, Amy J. 2001. Taeniatherum caput-medusae. In: Fire Effects Information System, [Online]. U.S. Department of Agriculture, Forest Service, Rocky Mountain Research Station, Fire Sciences Laboratory (Producer). Available: http://www.fs.fed.us/database/feis/ [2013, November 7].

Bovey, Rodney W.; LeTourneau, Duane; Erickson, Lambert C. 1960. The chemical composition of medusahead and downy brome. Weeds. 9: 307-311.

Christensen, M. Dale; Young, James A.; Evans, Raymond A. 1974. Control of annual grasses and revegetation in ponderosa pine woodlands. Journal of Range Management. 27(2): 143-145.

Clausnitzer DW, Borman MM, Johnson DE (1999) Competition between Elymus elymoides and Taeniatherum caput-medusae. Weed Sci. 47: 720-728

Cronquist, Arthur; Holmgren, Arthur H.; Holmgren, Noel H.; [and others]. 1977. Intermountain flora: Vascular plants of the Intermountain West, U.S.A. Vol. 6. The Monocotyledons. New York: Columbia University Press. 584 p.

Davies KW, Svejcar TJ (2008) Comparison of medusahead-invaded and noninvaded Wyoming big sagebrush steppe in Southeastern Oregon. Rangeland Ecol Manag 61: 623-629

Davies KW (2011) Plant community diversity and native plant abundance decline with increasing abundance of an exotic annual grass. Oecologia 167: 481-491.

Davies KW, Nafus AM, Madsen MD (2013) Medusahead invasion along unimproved roads, animal trails, and random transects. West N Am Naturalist 73: 54-59.

Furbush, Paul. 1953. Control of medusa-head on California ranges. Journal of Forestry. 51: 118-121.

Harris GA, Wilson AM (1970) Competition for moisture among seedlings of annual and perennial grasses as influenced by root elongation at low temperatures. Ecology 51: 530-534

Hironaka M, Sindelar BW (1975) Growth characteristics of squirreltail seedlings in competition with medusahead. J Range Manage 28: 283-285

Hironaka M (1961) The relative rate of root development of cheatgrass and medusahead. J

667 Range Manage 14: 263-267

Knapp, Paul A. 1998. Spatio-temporal patterns of large grassland fires in the Intermountain West, U.S.A. Global Ecology and Biogeography Letters. 7(4): 259-273.

Leffler AJ, James JJ, Monaco TA (2013) Temperature and functional traits influence differences in nitrogen uptake capacity between native and invasive grasses. Oecologia 171: 51-60.

Major, J.; McKell, C. M.; Berry, L. J. 1960. Improvement of medusahead-infested rangeland. Leaflet 123. Davis, CA: University of California, California Agricultural Experiment Station. 6 p

Miller, Heather C.; Clausnitzer, David; Borman, Michael M. 1999. Medusahead. In: Sheley, Roger L.; Petroff, Janet K., eds. Biology and management of noxious rangeland weeds. Corvallis, OR: Oregon State University Press: 271-281.

Monaco TA, Osmond TM, Dewey SA (2005) Medusahead control with fall- and spring-applied

762 herbicides on northern Utah foothills. Weed Technol 19: 653-658.

Savage, David E.; Young, James A.; Evans, Raymond A. 1969. Utilization of medusahead and downy brome caryopses by chukar partridge. Journal of Wildlife Management. 33(4): 975-978.

Sharp, Lee A.; Hironaka, M.; Tisdale, E. W. 1957. Viability of medusa-head (Elymus caput-medusae L.) seed collected in Idaho. Journal of Range Management. 10: 123-126.

Swenson, Charles F.; LeTourneau, Duane; Erickson, Lambert C. 1964. Silica in medusahead. Weeds. 12: 16-18

USDA PLANTS database

n.d. Zimmerman, J. Medusahead: Economic Impact and Control in Nevada. Fact Sheet. University of Nevada.

Young K, Mangold J (2008) Medusahead (Taeniatherum caput-medusae) outperforms squirreltail (Elymus elymoides) through interference and growth rate. Invasive Plant Sci Manage 1: 73-8

Notes

**annual**

monocot

Medusahead and cheatgrass are often in competition with each other

Score Interpretation

While different users will have different concepts of what constitutes various levels of invasiveness (e.g., what is “highly invasive” vs. “moderately invasive” may differ among management agencies), we divided the ranks into six blocks in Appendix A. We consider species with scores ≥80 as “Extremely Invasive” and species with scores 70–79 as “Highly Invasive;” both of these groups are composed of species estimated to be very threaten­ing to Alaska. Species with scores of 60–69 as “Moderately Invasive” and scores of 50–59 represent “Modestly Invasive” species; both of these groups still pose significant risks to ecosystems. Species with scores of 40–49 are “Weakly Invasive”, and <40 are considered “Very Weakly Invasive.” These last two groups generally have not been shown to significantly alter ecosystem processes and communities elsewhere and probably do not require as much attention as the other species.

**Alberta non-native plant invasiveness ranking form**

(Adapted from Carlson et al. 2008)

| Scientific name: | *Tribulus terrestris* |
| --- | --- |
| Common name: | Puncturevine |
| Assessor: | Shauna-Lee Chai |
| Reviewers: | Lisa Scott |
| Date: | December 2, 2013 |

Outcome score:

1. Climatic Comparison

This species is present or may potentially establish in the following natural regions:

|  | Collected in Alberta regions | CLIMEX similarity in 1975 | CLIMEX similarity in 2050 |
| --- | --- | --- | --- |
| Boreal | No | 0.798 | 0.845 |
| Parkland | No | 0.828 | 0.798 |
| Foothills | No | 0.836 | 0.822 |
| Grassland | No | 0.874 | 0.845 |
| Rocky Mountains | No | 0.778 | 0.776 |
| Shield | No | 0.833 | 0.869 |

1. Invasiveness Ranking Total (Total answered^1^ points possible) Total score

| 1. Ecological impact | 40(40) | 16 |
| --- | --- | --- |
| 1. Biological characteristic and dispersal ability | 25(25) | 17 |
| 1. Ecological amplitude and distribution | 25(25) | 16 |
| 1. Feasibility of control | 10(10) | 5 |
| Outcome score | 100(100)^b^ | ^a^54 |
| Relative maximum score^2^ | 54 | *Modestly Invasive* |

^1^For questions answered “unknown” do not include point value for the question in parentheses for “Total answered points possible.”

^2^Calculated as a/b x 100.

1. Climatic Comparison:
   1. Has this species ever been collected or documented in Alberta?

__Yes – continue to 1.2

x No – continue to 2.1

1.2 Which natural region has it been collected or documented? Proceed to section B. Invasiveness Ranking.

__Boreal

__Rockies

__Grassland

__Foothills

__Parkland

__Shield

Documentation:

Sources of information: ANPC Rogues gallery, ACIMS, PLANTS database, GBIF

2.1 Is there a 70 percent or higher similarity (based on CLIMEX climate matching) between climates anywhere the species currently occurs and

a. Boreal -Yes

b. Rockies -Yes

c. Grassland -Yes

d. Foothills -Yes

e. Parkland -Yes

f. Shield -Yes

-If “no” is answered for all regions, reject species from consideration

Documentation:

Sources of information:

1. Invasiveness Ranking
2. Ecological Impact
   1. Impact on Natural Ecosystem Processes
3. No perceivable impact on ecosystem processes 0
4. Has the potential to influence ecosystem processes to a minor degree

(e.g., has a perceivable but mild influence on soil nutrient availability) 3

1. Has the potential to cause significant alteration of ecosystem processes (e.g., increases sedimentation rates along streams or coastlines, reduces open water

that are important to waterfowl) 7

1. May cause major, possibly irreversible, alteration or disruption of ecosystem processes (e.g., the species alters geomorphology; hydrology; or affects fire frequency, altering community composition; species fixes substantial levels of nitrogen in the soil making soil unlikely to support certain native plants or more likely to favor non-native species) 10

u. Unknown

Score: 3

Documentation: capable of massive population increase over a short time (Squires 1979)

Identify ecosystem processes impacted:

Rationale:

Sources of information:

- 1. Impact on Natural Community Structure

1. No perceived impact; establishes in an existing layer without influencing its

structure 0

1. Has the potential to influence structure in one layer (e.g., changes the density

of one layer) 3

1. Has the potential to cause significant impact in at least one layer (e.g., creation

of a new layer or elimination of an existing layer) 7

1. Likely to cause major alteration of structure (e.g., covers canopy, eradicating

most or all layers below) 10

1. Unknown

Score:3

Documentation:

Identify type of impact or alteration:

Rationale:

Sources of information:

- 1. Impact on Natural Community Composition

1. No perceived impact; causes no apparent change in native populations 0
2. Has the potential to influence community composition (e.g., reduces the

number of individuals in one or more native species in the community) 3

1. Has the potential to significantly alters community composition (e.g., produces

a significant reduction in the population size of one or more native species in

the community) 7

1. Likely to cause major alteration in community composition (e.g., results in the extirpation of one or several native species, reducing biodiversity or change the community composition towards species exotic to the natural community) 10

u. Unknown

Score: 3

Documentation:

Identify type of impact or alteration:

Rationale:

Sources of information:

- 1. Impact on higher trophic levels (cumulative impact of this species on the animals,

fungi, microbes, and other organisms in the community it invades)

1. Negligible perceived impact 0
2. Has the potential to cause minor alteration 3
3. Has the potential to cause moderate alteration (minor reduction in

nesting/foraging sites, reduction in habitat connectivity, interference with

native pollinators, injurious components such as spines, toxins) 7

1. Likely to cause severe alteration of higher trophic populations (extirpation or endangerment of an existing native species/population, or significant reduction

in nesting or foraging sites) 10

u. Unknown

Score: 7

Documentation: unpalatable to grazers (Randall 2001). Spines injure wildlife (Holm et al. 1977)

Identify type of impact or alteration:

Rationale:

Sources of information:

Total Possible:40

Total:16

1. Biological Characteristics and Dispersal Ability
   1. Mode of reproduction
2. Not aggressive reproduction (few [0-10] seeds per plant and no

vegetative reproduction) 0

1. Somewhat aggressive (reproduces only by seeds (11-1,000/m2) 1
2. Moderately aggressive (reproduces vegetatively and/or by a moderate

amount of seed, <1,000/m2) 2

1. Highly aggressive reproduction (extensive vegetative spread and/or

many seeded, >1,000/m2) 3

u. Unknown

Score: 3

Documentation: no vegetative reproduction (Squires 1979)

Describe key reproductive characteristics (including seeds per plant): 400 fruits/plant, each flower with 15-20 seeds. Thousands of seed may be produced by 1 plant (Squires 1979)

Rationale:

Sources of information:

- 1. Innate potential for long-distance dispersal (bird dispersal, sticks to animal hair, buoyant fruits, wind-dispersal)

1. Does not occur (no long-distance dispersal mechanisms) 0
2. Infrequent or inefficient long-distance dispersal (occurs occasionally

despite lack of adaptations) 2

1. Numerous opportunities for long-distance dispersal (species has

adaptations such as pappus, hooked fruit-coats, etc.) 3

1. Unknown

Score:3

Documentation: water and birds (Randall 2001). Fruit is dispersed by adhering to the feet of animals and humans or vehicle and bicycle tyres. Fruit also sticks to sheep wool and is often found in hay, straw and manure (Smith 2002).

Identify dispersal mechanisms:

Rationale:

Sources of information:

- 1. Potential to be spread by human activities (both directly and indirectly – possible mechanisms include: commercial sales, use as forage/revegetation, spread along highways, transport on boats, contamination, etc.)

1. Does not occur 0
2. Low (human dispersal is infrequent or inefficient) 1
3. Moderate (human dispersal occurs) 2
4. High (there are numerous opportunities for dispersal to new areas) 3

u. Unknown

Score: 3

Documentation:vehicles, machinery, animals

Identify dispersal mechanisms:

Rationale:

Sources of information:

- 1. Allelopathic

1. no 0
2. yes 2
3. unknown

Score:2

Documentation:Verdu et al. 1999

Describe effect on adjacent plants:

Rationale:

Sources of information:

- 1. Competitive ability

a. Poor competitor for limiting factors 0

b. Moderately competitive for limiting factors 1

c. Highly competitive for limiting factors and/or nitrogen fixing ability 3

u. Unknown

Score:1

Documentation: Can tolerate dry soil. The deep taproot that the plant forms provides the mechanism for acquiring more water. Due to its ability to extract soil moisture, water losses due to T. terrestris are of economic concern for the agriculture, because of the plant’s ability to extract soil moisture from great depths and to compete well with crops (Holm et al 1991). It is a poor competitor where perennials exist however (Squires 1968)

Evidence of competitive ability:

Rationale:

Sources of information:

- 1. Forms dense thickets, climbing or smothering growth habit, or otherwise taller than the surrounding vegetation

1. No 0
2. Forms dense thickets 1
3. Has climbing or smothering growth habit, or otherwise taller than the surrounding

vegetation 2

u. Unknown

Score:0

Documentation: **annual** (Nikolova and Vassilev 2011). But not dense (Randall 2001). Other reports list ‘dense’ mats formed. Puncturevine forms extremely dense mats when there is limited competitive vegetation and other conditions (soil, climate) are ideal. Has also been observed on rare occasion to climb on other plants (native and non-native) and appear more hedge like (Scott pers. comm).

Describe growth form:

Rationale:

Sources of information:

- 1. Germination requirements

1. Requires open soil and disturbance to germinate 0
2. Can germinate in vegetated areas but in a narrow range or in special conditions 2
3. Can germinate in existing vegetation in a wide range of conditions 3

u. Unknown

Score:2

Documentation: shade intolerant (Nikolova and Vassilev 2011). This plant reproduces mainly through seed.

Germination percentage of T. terrestris is very low under natural and laboratory conditions. Seeds germination and seedlings establishment in this species are vulnerable to environmental stress and produce very a limited number of plants. The growth of T. terrestris plants is very slow and they produce scanty biomass. The main problem of the reproduction of this species is the low and irregular germination of the seeds (Nikolova & Vassilev 2011).

Describe germination requirements:

Rationale:

Sources of information:

- 1. Other species in the genus invasive in Alberta or elsewhere

1. No 0
2. Yes 3

u. Unknown

Score:3

Documentation: Tribulus cistoides, T. longipetalus

Species:

Sources of information:

2.9 Aquatic, wetland, or riparian species

a. Not invasive in wetland communities 0

b. Invasive in riparian communities 1

c. Invasive in wetland communities 3

u. Unknown

Score: 0

Documentation: Randall 2001

Describe type of habitat:

Rationale:

Sources of information:

Total Possible:25

Total:17

1. Distribution
   1. Is the species highly domesticated or a weed of agriculture
2. No 0
3. Is occasionally an agricultural pest 2
4. Has been grown deliberately, bred, or is known as a significant agricultural pest 4
5. Unknown

Score: 4

Documentation: Cultivated for medicinal and food supplement property (Nikolova and Vassilev 2011). Punturevine is a serious competitor of crops, especially in dry conditions. The burrs can be a

nuisance to pickers in orchards and contaminate harvested produce (CAB 2012).

Identify reason for selection, or evidence of weedy history:

Rationale:

Sources of information:

- 1. Known level of ecological impact in natural areas

1. Not known to cause impact in any other natural area 0
2. Known to cause impacts in natural areas, but in dissimilar habitats and

climate zones than exist in regions of Alberta 1

1. Known to cause low impact in natural areas in similar habitats and climate

zones to those present in Alberta 3

1. Known to cause moderate impact in natural areas in similar habitat and

climate zones 4

1. Known to cause high impact in natural areas in similar habitat and climate

zones 6

u. Unknown

Score:4

Documentation:

Identify type of habitat and states or provinces where it occurs:

Sources of information:

- 1. Role of anthropogenic and natural disturbance in establishment

1. Requires anthropogenic disturbances to establish 0
2. May occasionally establish in undisturbed areas but can readily establish in

areas with natural disturbances 3

1. Can establish independent of any known natural or anthropogenic disturbances 5

u. Unknown

Score:0

Documentation: A weed of cultivated fields, waste places and degraded pasture (Squires 1979)

Identify type of disturbance:

Rationale:

Sources of information:

- 1. Current global distribution

1. Occurs in one or two continents or regions (e.g., Mediterranean region) 0
2. Extends over three or more continents 3
3. Extends over three or more continents, including successful introductions in

arctic or subarctic regions 5

u. Unknown

Score:3

Documentation: Europe, Africa, Asia, North America, Australia

Describe distribution:

Rationale:

Sources of information:

- 1. Extent of the species Canada range and/or occurrence of formal state or provincial listing

1. 0-5 percent of the states/provinces 0
2. 6-20 percent of the states/provinces 2
3. 21-50 percent, and/or state/province listed as a problem weed

(e.g., “Noxious,” or “Invasive”) in 1 state or Canadian province 4

1. Greater than 50 percent, and/or identified as “Noxious” in 2 or more states or

Canadian provinces 5

u. Unknown

Score:5

Documentation: Noxious in Arizona, California, Colorado, Idaho, Iowa, Nevada, North Carolina, Oregon, Washington (USDA PLANTS database)

Identify provinces invaded:

Rationale:

Sources of information:

Total possible:25

Total:16

1. Feasibility of Control
   1. Seed banks
2. Seeds remain viable in the soil for less than 3 years 0
3. Seeds remain viable in the soil for between 3 and 5 years 2
4. Seeds remain viable in the soil for 5 years and more 3

u. Unknown

Score:2

Documentation:viability to 5 yrs

Identify longevity of seed bank

Rationale:

Sources of information:

- 1. Vegetative regeneration

1. No resprouting following removal of aboveground growth 0
2. Resprouting from ground-level meristems 1
3. Resprouting from extensive underground system 2
4. Any plant part is a viable propagule 3

u. Unknown

Score:0

Documentation: does not resprout (Scott pers. comm.)

Describe vegetative response:

Rationale:

Sources of information:

- 1. Level of effort required

1. Management is not required (e.g., species does not persist without repeated anthropogenic disturbance) 0
2. Management is relatively easy and inexpensive; requires a minor investment in human and financial resources 2
3. Management requires a major short-term investment of human and financial resources, or a moderate long-term investment 3
4. Management requires a major, long-term investment of human and financial resources 4

u. Unknown

Score:3

Documentation: mechanical, chemical and biocontrol-weevils

Identify types of control methods and time-term required:

Rationale:

Sources of information:

Total Possible: 10

Total:5

Total for 4 sections Possible: 100

Total for 4 sections: 54

References:

AISC 2012. Alberta Invasive Species Council Fact Sheet: Puncturevine

CAB 2012. Tribulus terrestris. Invasive Species Compendium. CAB International.

<http://www.cabi.org/isc/>

Carlson, M. 2008. Invasiveness Ranking System for Non-Native Plants of Alaska. USDA. Available at: http://www.fs.usda.gov/Internet/FSE_DOCUMENTS/fsbdev2_037575.pdf

Holm L.G., Plucknett D.L., Pancho J.V., Herberger J.P. (1991) The World’s Worst Weeds, University Press of Hawaii, Honolulu, p. 609.

Holm LG, Pancho JV, Herberger JP, Plucknett DL, 1979. A Geographical Atlas of World Weeds. New York, USA: John Wiley and Sons.

Nikolova, A. & Vassilev, A., 2011. A Study on Tribulus Terrestris L. Anatomy and Ecological Adaptation. Biotechnology & Biotechnological Equipment, 25(2), pp.2369–2372. Available at: http://diagnosisp.com/dp/journals/view_article.php?journal_id=1&archive=0&issue_id=32&article_id=1150 [Accessed December 2, 2013].

Randall 2001. Pacific Island Ecosystems at Risk (PIER): Tribulus terrestris.

Smith, Nicholas M. 2002. Weeds of the wet/dry tropics of Australia - a field guide. Environment Centre NT, Inc. 112 pp

Squires, V.R. 1979. The Biology of Australian weeds: Tribulus terrestris.

USDA PLANTS database

Verdu, A, M Mas, A Almirall. 1999. Allelopathic effects of Tribulus terrestris. Departamento Hortofruticultura, Botánica i Jardinería, Lérida, Spain. http://www.cabi.org/isc/Default.aspx?site=144&page=2540&LoadModule11=CABISEARCHRESULTS&LoadAction=LoadAbstract&term=au:%22Mas,+M.+T.%22&AbstractSearchTerm=au:%22Mas,+M.+T.%22&query=au:%22Mas,+M.+T.%22&AbstractID=20002302216

Nikolova, A. & Vassilev, A., 2011. a Study on Tribulus Terrestris L. Anatomy and Ecological Adaptation. Biotechnology & Biotechnological Equipment, 25(2), pp.2369–2372. Available at: http://diagnosisp.com/dp/journals/view_article.php?journal_id=1&archive=0&issue_id=32&article_id=1150 [Accessed December 2, 2013].

Notes

**annual**

Intolerant of freezing temps, tolerates salt and drought

It is a caltrop-A caltrop is a metal device, used to deter passage by vehicles with pneumatic tires or the hooves of horses; it has four projecting spikes so arranged that when three of the spikes are on the ground, the fourth points upward to poke a tire or hoof.) The seeds of puncturevine are enclosed in a hard caltrop-like case that can injure livestock, people, and pets when stepped on and can even puncture bicycle tires.

Score Interpretation

While different users will have different concepts of what constitutes various levels of invasiveness (e.g., what is “highly invasive” vs. “moderately invasive” may differ among management agencies), we divided the ranks into six blocks in Appendix A. We consider species with scores ≥80 as “Extremely Invasive” and species with scores 70–79 as “Highly Invasive;” both of these groups are composed of species estimated to be very threaten­ing to Alberta. Species with scores of 60–69 as “Moderately Invasive” and scores of 50–59 represent “Modestly Invasive” species; both of these groups still pose significant risks to ecosystems. Species with scores of 40–49 are “Weakly Invasive”, and <40 are considered “Very Weakly Invasive.” These last two groups generally have not been shown to significantly alter ecosystem processes and communities elsewhere and probably do not require as much attention as the other species.

**Alberta non-native plant invasiveness ranking form**

(Adapted from Carlson et al. 2008)

| Scientific name: | *Tamarix chinensis* (much hybridisation with *Tamarix ramosissima*.  Thus, this ranking can be considered for both species) |
| --- | --- |
| Common name: | Tamarisk, Chinese/saltcedar |
| Assessor: | Shauna-Lee Chai |
| Reviewers: | Tom Dudley |
| Date: | November 29, 2013 |

Outcome score:

1. Climatic Comparison

This species is present or may potentially establish in the following natural regions:

|  | Collected in Alberta regions | CLIMEX similarity in 1975 | CLIMEX similarity in 2050 |
| --- | --- | --- | --- |
| Boreal | No | 0.826 | 0.886 |
| Parkland | Yes (GBIF) | 0.939 | 0.889 |
| Foothills | No | 0.846 | 0.846 |
| Grassland | Yes | 0.885 | 0.878 |
| Rocky Mountains | No | 0.736 | 0.757 |
| Shield | No | 0.706 | 0.788 |

*present in gardens as ornamentals in Edmonton, Calgary, Medicine Hat and greenhouse/nurseries in Southern Alberta (Kimmel pers.comm.)

1. Invasiveness Ranking Total (Total answered^1^ points possible) Total score

| 1. Ecological impact | 40(40) | 40 |
| --- | --- | --- |
| 1. Biological characteristic and dispersal ability | 25(25) | 21 |
| 1. Ecological amplitude and distribution | 25(25) | 21 |
| 1. Feasibility of control | 10(10) | 5 |
| Outcome score | 100(100)^b^ | ^a^87 |
| Relative maximum score^2^ | 87 | *Extremely Invasive* |

^1^For questions answered “unknown” do not include point value for the question in parentheses for “Total answered points possible.”

^2^Calculated as a/b x 100.

1. Climatic Comparison:
   1. Has this species ever been collected or documented in Alberta?

xYes – continue to 1.2

__ No – continue to 2.1

Which natural region has it been collected or documented (see inset map)? Proceed to section B. Invasiveness Ranking.

__Boreal

__Rockies

x Grassland

__Foothills

x Parkland (central parkland)

__Shield

Documentation:

Sources of information: ANPC Rogues gallery, ACIMS, PLANTS database, GBIF. Tamarix drops out with increasing elevation, just as it does with increasing latitude and it is not likely to be able to survive in Rocky Mt habitats of Canada because of the combined environmental conditions of both elevation and latitude. It is also extremely unlikely to survive under boreal conditions, not because of temperature but because the conditions are not suitable for seed germination and establishment (Dudley pers. comm.).

2.1 Is there a 70 percent or higher similarity (based on CLIMEX climate matching) between climates anywhere the species currently occurs and

a. Boreal -Yes

b. Rockies -Yes

c. Grassland -Yes

d. Foothills -Yes

e. Parkland -Yes

f. Shield -Yes

-If “no” is answered for all regions, reject species from consideration

Documentation:

Sources of information:

1. Invasiveness Ranking
2. Ecological Impact
   1. Impact on Natural Ecosystem Processes
3. No perceivable impact on ecosystem processes 0
4. Has the potential to influence ecosystem processes to a minor degree

(e.g., has a perceivable but mild influence on soil nutrient availability) 3

1. Has the potential to cause significant alteration of ecosystem processes (e.g., increases sedimentation rates along streams or coastlines, reduces open water

that are important to waterfowl) 7

1. May cause major, possibly irreversible, alteration or disruption of ecosystem processes (e.g., the species alters geomorphology; hydrology; or affects fire frequency, altering community composition; species fixes substantial levels of nitrogen in the soil making soil unlikely to support certain native plants or more likely to favor non-native species) 10

u. Unknown

Score: 10

Documentation: The high evapotranspiration rates of saltcedar can lower the water table and alter the floristic composition in heavily infested area (Anderson 1982).

Tamarix increases the frequency, extent and intensity of riparian wildfire (Drus et al. 2012). Tamarix is highly flammable, both when green and when deciduous because dry foliage remains on the plant, while native riparian plants are generally intolerant of fire so are damaged or destroyed when riparian areas burn, while Tamarix grows back readily following fire. Evapotranspiration increased (Tamarix approx double the transpiration rates of native veg because of increased leaf surface area – Sala et al.1996) and attendant groundwater reduction. Channel narrowing and both erosion and deposition from dense cover (Graf 1982)

Identify ecosystem processes impacted:

Rationale:

Sources of information:

- 1. Impact on Natural Community Structure

1. No perceived impact; establishes in an existing layer without influencing its

structure 0

1. Has the potential to influence structure in one layer (e.g., changes the density

of one layer) 3

1. Has the potential to cause significant impact in at least one layer (e.g., creation

of a new layer or elimination of an existing layer) 7

1. Likely to cause major alteration of structure (e.g., covers canopy, eradicating

most or all layers below) 10

1. Unknown

Score:10

Documentation: In Bosque del Apache National Wildlife Refuge on the Rio Grande River, saltcedar occurs as a community dominant and in dense subcanopy zones under Rio Grande cottonwood, along with Goodding willow, mule's fat, stretchberry (New Mexico privet), desert false indigo (*Amorpha fruticosa*) and Russian-olive. Durkin and others describe several community types in the upper and middle Rio Grande watershed in which saltcedar is dominant or codominant (Ellis 2001). Only xeric species or halophytes can tolerate the understory environment of saltcedar (Brotherson et al. 1984). In much of the Utah Lake area saltcedar forms almost pure stands, and it is the most widespread introduced species around the lake. Eight of the 13 prevalent species in saltcedar communities are non-native (Brotherson et al. 1984).

Tamarix dominance is associated with reduced diversity of native plants and greater area in bare substrate in the Virgin River floodplain in Nevada, and mechanical tamarisk control has facilitated modest recovery of native species…but mechanical treatments also tend to promote other non-native plants that respond positively to soil disturbance – Ostoja et al., in press

Reduces aquatic invertebrate populations because foliage that falls into stream as organic litter, albeit relatively high in nutrient (N) content, is readily broken down by physical processes in streams so is not available for as long a period as native litter types (Going and Dudley 2008).

Identify type of impact or alteration:

Rationale:

Sources of information:

- 1. Impact on Natural Community Composition

1. No perceived impact; causes no apparent change in native populations 0
2. Has the potential to influence community composition (e.g., reduces the

number of individuals in one or more native species in the community) 3

1. Has the potential to significantly alters community composition (e.g., produces

a significant reduction in the population size of one or more native species in

the community) 7

1. Likely to cause major alteration in community composition (e.g., results in the extirpation of one or several native species, reducing biodiversity or change the community composition towards species exotic to the natural community) 10

u. Unknown

Score: 10

Documentation: forming essentially monotypic stands

Identify type of impact or alteration:

Rationale:

Sources of information:

- 1. Impact on higher trophic levels (cumulative impact of this species on the animals,

fungi, microbes, and other organisms in the community it invades)

1. Negligible perceived impact 0
2. Has the potential to cause minor alteration 3
3. Has the potential to cause moderate alteration (minor reduction in

nesting/foraging sites, reduction in habitat connectivity, interference with

native pollinators, injurious components such as spines, toxins) 7

1. Likely to cause severe alteration of higher trophic populations (extirpation or endangerment of an existing native species/population, or significant reduction

in nesting or foraging sites) 10

u. Unknown

Score:10

Documentation: Dense infestations have dried up springs and pools in California and New Mexico, eliminating habitat for fish and other animals (Duncan and McDaniel 1998). Less palatable to grazing animals than the native species (Horton 1977). Reduced native fish abundance in desert stream via reduction in aquatic invertebrates and

Increasing dominance is associated with decline in avian biodiversity. Tamarix stands do not support small mammal (Longland ) and herptile (Bateman ) populations at the same level as native vegetation and provide fewer food resources to wildlife. Tamarix invasion is listed by the U.S. Fish & Wildlife Service as one of the factors causing endangerment of numerous species, including SW willow flycatcher, yellow-billed cuckoo, and many other species – its abundance in western riparian areas, which are almost the most degraded yet important for biodiversity, is considered a major cause of riparian species declines (Dudley and DeLoach 2004).

Tamarisk also changes microbial assemablages and reduces potential for native species establishment – Meinhardt & Gehring 2012.

Identify type of impact or alteration:

Rationale:

Sources of information:

Total Possible:40

Total:40

1. Biological Characteristics and Dispersal Ability
   1. Mode of reproduction
2. Not aggressive reproduction (few [0-10] seeds per plant and no

vegetative reproduction) 0

1. Somewhat aggressive (reproduces only by seeds (11-1,000/m2) 1
2. Moderately aggressive (reproduces vegetatively and/or by a moderate

amount of seed, <1,000/m2) 2

1. Highly aggressive reproduction (extensive vegetative spread and/or

many seeded, >1,000/m2) 3

u. Unknown

Score:3

Documentation: Mature saltcedar plants are capable of producing 2.5 x 108 seeds per year (Stevens 2002). 17/cm^2^ reached the soil surface in a dense saltcedar stand over 1 growing season (Warren and Turner 1975). Also mature tamarisk plants reproduce vegetatively by adventitious roots (Brotherson and Field 1987)

Describe key reproductive characteristics (including seeds per plant):

Rationale:

Sources of information:

- 1. Innate potential for long-distance dispersal (bird dispersal, sticks to animal hair, buoyant fruits, wind-dispersal)

1. Does not occur (no long-distance dispersal mechanisms) 0
2. Infrequent or inefficient long-distance dispersal (occurs occasionally

despite lack of adaptations) 2

1. Numerous opportunities for long-distance dispersal (species has

adaptations such as pappus, hooked fruit-coats, etc.) 3

1. Unknown

Score:3

Documentation: Saltcedar seeds have small hairs on the apex of the seed coat and are readily dispersed by wind and water (Merkel and Hopkins 1957).

Identify dispersal mechanisms:

Rationale:

Sources of information:

- 1. Potential to be spread by human activities (both directly and indirectly – possible mechanisms include: commercial sales, use as forage/revegetation, spread along highways, transport on boats, contamination, etc.)

1. Does not occur 0
2. Low (human dispersal is infrequent or inefficient) 1
3. Moderate (human dispersal occurs) 2
4. High (there are numerous opportunities for dispersal to new areas) 3

u. Unknown

Score:2

Documentation: human dispersal is not very important for Tamarix, and it is not used in revegetation projects nor forage or other applications, although limited sales of some horticultural varieties are still conducted via the nursery industry and have potential for escape into natural environments – genes from horticultural types are present in wild populations (Gaskin and Kazmer 2009). There is little or no potential for seed or propagule transport by humans as seed viability is extremely short and sensitive to desiccation or damage. However, it is human alteration of riparian and wetland systems through various mechanisms that facilitates spread of Tamarix

Identify dispersal mechanisms:

Rationale:

Sources of information:

- 1. Allelopathic

1. no 0
2. yes 2
3. unknown

Score:2

Documentation: Tamarisk accumulates salt in special glands in its leaves, and then excretes it onto the leaf surface. Foliage of saltcedar is often covered with a bloom of salt. These salts accumulate in the surface layer of soil when plants drop their leaves. As surface soils become more saline over time, particularly along regulated rivers that are no longer subjected to annual flooding and scouring, germination and establishment of many native species become impaired (Busch and Smith 1995).

Describe effect on adjacent plants:

Rationale:

Sources of information:

- 1. Competitive ability

a. Poor competitor for limiting factors 0

b. Moderately competitive for limiting factors 1

c. Highly competitive for limiting factors and/or nitrogen fixing ability 3

u. Unknown

Score:3

Documentation: Saltcedar can obtain water at lower plant water potential and has higher water use efficiency than native riparian trees. The ability of tamarisk to closely regulate photosynthesis and leaf conductance during drought increases its survivability and competitive ability in arid and semiarid rangelands (Busch and Smith 1995).

Evidence of competitive ability:

Rationale:

Sources of information:

- 1. Forms dense thickets, climbing or smothering growth habit, or otherwise taller than the surrounding vegetation

1. No 0
2. Forms dense thickets 1
3. Has climbing or smothering growth habit, or otherwise taller than the surrounding

vegetation 2

u. Unknown

Score:2

Documentation: In many systems can represent close to 100% of vegetative cover, particularly owing to massive germination events following flood scouring of riversides, receding lake margins, etc. – Shafroth et al. 2005

Describe growth form:

Rationale:

Sources of information:

- 1. Germination requirements

1. Requires open soil and disturbance to germinate 0
2. Can germinate in vegetated areas but in a narrow range or in special conditions 2
3. Can germinate in existing vegetation in a wide range of conditions 3

u. Unknown

Score:0

Documentation: Germination requires direct contact with water or extremely high humidity, and is very rapid (<24 hours). Seeds require a moist, fine-grained (silt or smaller particle size) substrate for germination (Merkel and Hopkins 1957). It does not germinate well in vegetated areas, or if it does, it is readily outcompeted by existing plants – requires open substrate

Describe germination requirements:

Rationale:

Sources of information:

- 1. Other species in the genus invasive in Alberta or elsewhere

1. No 0
2. Yes 3

u. Unknown

Score:3

Documentation: *Tamarix ramosissima, Tamarix parviflora*. Species is hybridising.

Species:

Sources of information:

2.9 Aquatic, wetland, or riparian species

a. Not invasive in wetland communities 0

b. Invasive in riparian communities 1

c. Invasive in wetland communities 3

u. Unknown

Score: 3

Documentation: Once established along the major drainages, tamarisk successfully invaded outlying ephemeral water courses, isolated marshes, and springs via its windblown seeds and possibly due to occasional plantings (Graf 1982). Increased evapotranspiration due to the invasion of Tamarix in the United States costs an estimated $65–180 million per year in reduced municipal and agricultural water supplies. In addition to raising water costs, the presence of sediment-trapping Tamarix stands has narrowed river channels and obstructed over-bank flows throughout the western United States, increasing flood damages by as much as $50 million annually (Zavaleta, 2000)

Describe type of habitat:

Rationale:

Sources of information:

Total Possible:25

Total:21

1. Distribution
   1. Is the species highly domesticated or a weed of agriculture
2. No 0
3. Is occasionally an agricultural pest 2
4. Has been grown deliberately, bred, or is known as a significant agricultural pest 4
5. Unknown

Score:4

Documentation: introduced for sale as an ornamental shrub and a windbreak species (Tellman 1997)

Identify reason for selection, or evidence of weedy history:

Rationale:

Sources of information:

- 1. Known level of ecological impact in natural areas

1. Not known to cause impact in any other natural area 0
2. Known to cause impacts in natural areas, but in dissimilar habitats and

climate zones than exist in regions of Alberta 1

1. Known to cause low impact in natural areas in similar habitats and climate

zones to those present in Alberta 3

1. Known to cause moderate impact in natural areas in similar habitat and

climate zones 4

1. Known to cause high impact in natural areas in similar habitat and climate

zones 6

u. Unknown

Score:6

Documentation: Tamarisk is a problem in many natural areas and state and national parks and monuments in the western U.S. (Loope et al. 1988)

Identify type of habitat and states or provinces where it occurs:

Sources of information:

- 1. Role of anthropogenic and natural disturbance in establishment

1. Requires anthropogenic disturbances to establish 0
2. May occasionally establish in undisturbed areas but can readily establish in

areas with natural disturbances 3

1. Can establish independent of any known natural or anthropogenic disturbances 5

u. Unknown

Score:3

Documentation: Tamarisk communities are frequently associated with past disturbances and/or changes in historic disturbance regimes (Dudley et al. 2000)

Identify type of disturbance:

Rationale:

Sources of information:

- 1. Current global distribution

1. Occurs in one or two continents or regions (e.g., Mediterranean region) 0
2. Extends over three or more continents 3
3. Extends over three or more continents, including successful introductions in

arctic or subarctic regions 5

u. Unknown

Score:3

Documentation: N. America, Africa, Europe, Asia

Describe distribution:

Rationale:

Sources of information:

- 1. Extent of the species Canada range and/or occurrence of formal state or provincial listing

1. 0-5 percent of the states/provinces 0
2. 6-20 percent of the states/provinces 2
3. 21-50 percent, and/or state/province listed as a problem weed

(e.g., “Noxious,” or “Invasive”) in 1 state or Canadian province 4

1. Greater than 50 percent, and/or identified as “Noxious” in 2 or more states or

Canadian provinces 5

u. Unknown

Score:5

Documentation: Noxious in California, Nevada, Arizona, Colorado, Montana, New Mexico, North Dakota, South Dakota, Taxas, Wyoming (USDA PLANTS)

Identify provinces invaded:

Rationale:

Sources of information:

Total possible:25

Total:21

1. Feasibility of Control
   1. Seed banks
2. Seeds remain viable in the soil for less than 3 years 0
3. Seeds remain viable in the soil for between 3 and 5 years 2
4. Seeds remain viable in the soil for 5 years and more 3

u. Unknown

Score:0

Documentation: Tamarisk seeds are short-lived-few days, and do not form a persistent seed bank.

Identify longevity of seed bank

Rationale:

Sources of information:

- 1. Vegetative regeneration

1. No resprouting following removal of aboveground growth 0
2. Resprouting from ground-level meristems 1
3. Resprouting from extensive underground system 2
4. Any plant part is a viable propagule 3

u. Unknown

Score:1

Documentation:Resprouts after top-kill (Brotherson and Dean 1987)

Describe vegetative response:

Rationale:

Sources of information:

- 1. Level of effort required

1. Management is not required (e.g., species does not persist without repeated anthropogenic disturbance) 0
2. Management is relatively easy and inexpensive; requires a minor investment in human and financial resources 2
3. Management requires a major short-term investment of human and financial resources, or a moderate long-term investment 3
4. Management requires a major, long-term investment of human and financial resources 4

u. Unknown

Score:4

Documentation: Once tamarisk is well established it is very difficult and expensive to control, as any stress imposed by control methods (e.g. fire, herbicides, and cutting) increases flowering and seed production; and the entire root system must be killed in order to prevent sprouting. Monitoring, prevention, early detection and local eradication remain the most effective approach to controlling tamarisk (Chen 2001). ). Tamarix chinensis/ramosissima has also been the target of a multi-million dollar biological control program that led to establishment of a specialist beetle, Diorhabda spp., in many U.S. states and D. carinulata is likely physiologically capable of establishment in southern Canada if this is eventually seen as a useful approach to management.

Identify types of control methods and time-term required:

Rationale:

Sources of information:

Total Possible: 10

Total: 5

Total for 4 sections Possible: 100

Total for 4 sections: 87

References:

Anderson, J. E. 1982. Factors controlling transpiration and photosynthesis in Tamarix x chinensis Lour. Ecology 63:48-56

Brotherson, Jack D.; Field, Dean. 1987. Tamarix: impacts of a successful weed. Rangelands. 9(3): 110-112.

Brotherson, Jack D.; Carman, John G.; Szyska, Lee A. 1984. Stem-diameter age relationships of Tamarix ramosissima in central Utah. Journal of Range Management. 37(4): 362-364

Busch, David E.; Smith, Stanley D. 1995. Mechanisms associated with decline of woody species in riparian ecosystems of the southwestern U.S. Ecological Monographs. 65(3): 347-370.

Carlson, M. 2008. Invasiveness Ranking System for Non-Native Plants of Alaska. USDA. Available at: http://www.fs.usda.gov/Internet/FSE_DOCUMENTS/fsbdev2_037575.pdf

Chen, Linus Y. 2001. Cost savings from properly managing endangered species habitats. Natural Areas Journal. 21(2): 197-203

Drus, G., Dudley, T., Brooks, m and Matchett, J. (2012) The effect of leaf beetle herbivory on the fire behaviour of tamarisk (Tamarix ramosissima Lebed .).

Dudley, Tom L.; DeLoach, C. Jack; Lovich, Jeffrey E.; Carruthers, Raymond I. 2000. Saltcedar invasion of western riparian areas: impacts and new prospects for control. In: In: New insights and new incites in natural resource management: Transactions, 65th North American wildlife and natural resources conference; 2000 March 24-28; Rosemont, IL. Washington, DC: Wildlife Management Institute: 345-381.

Dudley ,T.L. & C.J.DeLoach. 2004. Saltcedar (*Tamarix* spp.), endangered species, and biological weed control-can they mix? Weed Technology 18:1542–1551

Duncan, Keith W.; McDaniel, Kirk C. 1998. Saltcedar (Tamarix spp.) management with imazapyr. Weed Technology. 12(2): 337-344

Ellis, Lisa M. 2001. Short-term response of woody plants to fire in a Rio Grande riparian forest, central New Mexico, USA. Biological Conservation. 97: 159-170.

Gaskin J.F. & D.J. Kazmer. 2009. Introgression between invasive saltcedars (*Tamarix chinensis* and *T. ramosissima*) in the USA. Biological Invasions 11:1121-1130.

Going, B. M., & Dudley, T. L. (2008). Invasive riparian plant litter alters aquatic insect growth. Biological Invasions, 10(7), 1041-1051.

Graf, William L. 1982. Tamarisk and river-channel management. Environmental Management. 6(4): 283-296

Horton, Jerome S. 1977. The development and perpetuation of the permanent tamarisk type in the phreatophyte zone of the Southwest. In: Johnson, R. Roy; Jones, Dale A., tech. coords. Importance, preservation and management of riparian habitat: a symposium: Proceedings; 1977 July 9; Tucson, AZ. General Technical Report RM-43. Fort Collins, CO: U.S. Department of Agriculture, Forest Service, Rocky Mountain Forest and Range Experiment Station: 124-127.

Loope, Lloyd L.; Sanchez, Peter G.; Tarr, Peter W.; Loope, Walter L.; Anderson, Richard L. 1988. Biological invasions of arid land nature reserves. Biological Conservation. 44: 95-118.

[Meinhardt K.A](http://www.ncbi.nlm.nih.gov/pubmed?term=Meinhardt%20KA%5BAuthor%5D&cauthor=true&cauthor_uid=22611852). & C.A.[Gehring](http://www.ncbi.nlm.nih.gov/pubmed?term=Gehring%20CA%5BAuthor%5D&cauthor=true&cauthor_uid=22611852). 2012. Disrupting mycorrhizal mutualisms: a potential mechanism by which exotic tamarisk outcompetes native cottonwoods. Ecological Applications 22:532-549.

Merkel, Daniel L.;Hopkins, Harold H. 1957. Life history of the salt cedar (Tamarix gallica L.). Transactions of the Kansas Academy of Science. 60(4): 360-369.

Ostoja, S.M., M.L. Brooks, T.L. Dudley & S.R. Lee. 2014. Short-term vegetation response following mechanical control of saltcedar (*Tamarix* spp.) on the Virgin River, USA. Invasive Plant Science & Management (in press).

Sala A, Smith SD, Devitt DA (1996) Water use by *Tamarix ramosissima* and associated phreatophytes in a Mojave Desert floodplain. Ecological Applications 6:888-898.

Stevens, Larry E. 2002. Exotic tamarisk on the Colorado Plateau, [Online]. In: Grahame, John D.; Sisk, Thomas D., eds. Canyons, cultures and environmental change: an introduction to the land-use history of the Colorado Plateau. U.S. Geological Survey (Producer). Available: http://www.cpluhna.nau.edu/Biota/tamarisk.htm

Tellman, Barbara. 1997. Exotic pest plant introduction in the American Southwest. Desert Plants. 13(1): 3-10

USDA PLANTS database

Warren, Douglas K.; and Raymond M. Turner. 1975. Saltcedar (Tamarix chinensis) seed production, seedling establishment, and response to inundation. Journal of the Arizona Academy of Science. 10: 135-144.

Zavaleta, E. S. 2000. In Invasive Species in a Changing World (eds Hobbs, R. J. & Mooney, H. A.) Island, Washington DC.

Zouhar, Kris. 2003. Tamarix spp. In: Fire Effects Information System, [Online]. U.S. Department of Agriculture, Forest Service, Rocky Mountain Research Station, Fire Sciences Laboratory (Producer). Available: http://www.fs.fed.us/database/feis/ [ 2013, November 29].

Notes

Life form-tree shrub

There is probably not another genus of plants as well known as the tamarisks in which the species are so poorly understood or separated on more obscure characters. Each species has a distinct distribution in Eurasia, but they have hybridized in southwestern United States. Gaskin and Schaal as well as Allred present DNA data showing extensive hybridisation of T. chinensis and T. ramosissima.

It is not really possible to have a review of just the T. chinensis species, as the vast majority of invasive Tamarix are hybrids between T. chinensis and T. ramosissima, and only rarely are true species found in the field. This is partly responsible for their success as invasive weeds, as the genetic heterogeneity has facilitated rapid evolution of the plants into new environments, including their spread northward in Montana and subsequently into Canada/Alta. In fact, there is a very interesting pattern in which invasive plants tend to have greater contribution of T. ramosissima genes in the north, and T. chinensis genetic material in the south of their range Friedman et al. 2008, Williams 2013, which has interesting implications for the impact/efficacy of the Diorhabda beetles introduced for their biocontrol (Hultine et al. 2013). Due to the hybridisation issue, this review can be considered for both T. ramosissima and T. chinensis (Dudley pers. comm.).

Tamarisk can tolerate an extreme range of environmental conditions, and Brotherson and von Winkel suggest a general purpose genotype in saltcedar that gives it the capability to "exploit a wide spectrum of habitats." Phenotypic plasticity, ecotypic differentiation and high genetic variation suggest a high invasive potential. However it is highly unlikely to establish outside of riparian and wetland systems, and where substrates are periodically left bare and open for seed germination and establishment – even though it is associated with arid and semi-arid ecosystems, it is only where there is periodically high water available since the plants require substantial moisture for initial seedling establishment (Dudley pers.comm.)

Score Interpretation

While different users will have different concepts of what constitutes various levels of invasiveness (e.g., what is “highly invasive” vs. “moderately invasive” may differ among management agencies), we divided the ranks into six blocks in Appendix A. We consider species with scores ≥80 as “Extremely Invasive” and species with scores 70–79 as “Highly Invasive;” both of these groups are composed of species estimated to be very threaten­ing to Alberta. Species with scores of 60–69 as “Moderately Invasive” and scores of 50–59 represent “Modestly Invasive” species; both of these groups still pose significant risks to ecosystems. Species with scores of 40–49 are “Weakly Invasive”, and <40 are considered “Very Weakly Invasive.” These last two groups generally have not been shown to significantly alter ecosystem processes and communities elsewhere and probably do not require as much attention as the other species.

**Alberta non-native plant invasiveness ranking form**

(Adapted from Carlson et al. 2008)

| Scientific name: | *Halogeton glomeratus* |
| --- | --- |
| Common name: | Saltlover |
| Assessor: | Shauna-Lee Chai |
| Reviewer: | Joseph Ditomaso |
| Date: | November 29, 2013 |

Outcome score:

1. Climatic Comparison

This species is present or may potentially establish in the following natural regions:

|  | Collected in Alberta regions | CLIMEX similarity in  1975 | CLIMEX similarity in 2050 |
| --- | --- | --- | --- |
| Boreal | No | 0.777 | 0.777 |
| Parkland | No | 0.801 | 0.828 |
| Foothills | No | 0.823 | 0.813 |
| Grassland | No | 0.856 | 0.874 |
| Rocky Mountains | No | 0.776 | 0.761 |
| Shield | No | 0.828 | 0.739 |

1. Invasiveness Ranking Total (Total answered^1^ points possible) Total score

| 1. Ecological impact | 40(40) | 20 |
| --- | --- | --- |
| 1. Biological characteristic and dispersal ability | 25(25) | 10 |
| 1. Ecological amplitude and distribution | 25(25) | 15 |
| 1. Feasibility of control | 10(10) | 7 |
| Outcome score | 100(100)^b^ | ^a^52 |
| Relative maximum score^2^ | 52 | *Modestly Invasive* |

^1^For questions answered “unknown” do not include point value for the question in parentheses for “Total answered points possible.”

^2^Calculated as a/b x 100.

1. Climatic Comparison:
   1. Has this species ever been collected or documented in Alberta?

__Yes – continue to 1.2

x No – continue to 2.1

Which natural region has it been collected or documented (see inset map)? Proceed to section B. Invasiveness Ranking.

__Boreal

__Rockies

__Grassland

__Foothills

__Parkland

__Shield

Documentation:

Sources of information: ANPC Rogues gallery, ACIMS, PLANTS database, GBIF

2.1 Is there a 70 percent or higher similarity (based on CLIMEX climate matching) between climates anywhere the species currently occurs and

a. Boreal -Yes

b. Rockies -Yes

c. Grassland -Yes

d. Foothills -Yes

e. Parkland -Yes

f. Shield -Yes

-If “no” is answered for all regions, reject species from consideration

Documentation:

Sources of information:

1. Invasiveness Ranking
2. Ecological Impact
   1. Impact on Natural Ecosystem Processes
3. No perceivable impact on ecosystem processes 0
4. Has the potential to influence ecosystem processes to a minor degree

(e.g., has a perceivable but mild influence on soil nutrient availability) 3

1. Has the potential to cause significant alteration of ecosystem processes (e.g., increases sedimentation rates along streams or coastlines, reduces open water

that are important to waterfowl) 7

1. May cause major, possibly irreversible, alteration or disruption of ecosystem processes (e.g., the species alters geomorphology; hydrology; or affects fire frequency, altering community composition; species fixes substantial levels of nitrogen in the soil making soil unlikely to support certain native plants or more likely to favor non-native species) 10

u. Unknown

Score: 7

Documentation: Causes increases in pH, exchangeable sodium, potassium, magnesium, electrical conductivity, and decreases in water percolation. High salts inhibit micro-organisms aiding nitrification, which depresses plant growth (Cronin et al 1965). Changes not completely irreversible (Ditomaso, pers.comm.).

Identify ecosystem processes impacted:

Rational:

Sources of information:

- 1. Impact on Natural Community Structure

1. No perceived impact; establishes in an existing layer without influencing its

structure 0

1. Has the potential to influences structure in one layer (e.g., changes the density

of one layer) 3

1. Has the potential to cause significant impact in at least one layer (e.g., creation

of a new layer or elimination of an existing layer) 7

1. Likely to cause major alteration of structure (e.g., covers canopy, eradicating

most or all layers below) 10

1. Unknown

Score:3

Documentation: as above

Identify type of impact or alteration:

Rational:

Sources of information:

- 1. Impact on Natural Community Composition

1. No perceived impact; causes no apparent change in native populations 0
2. Has the potential to influence community composition (e.g., reduces the

number of individuals in one or more native species in the community) 3

1. Has the potential to significantly alters community composition (e.g., produces

a significant reduction in the population size of one or more native species in

the community) 7

1. Likely to cause major alteration in community composition (e.g., results in the extirpation of one or several native species, reducing biodiversity or change the community composition towards species exotic to the natural community) 10

u. Unknown

Score:3

Documentation: as above. Not an extremely competitive plant (Whitson et al. 2000)

Identify type of impact or alteration:

Rational:

Sources of information:

- 1. Impact on higher trophic levels (cumulative impact of this species on the animals,

fungi, microbes, and other organisms in the community it invades)

1. Negligible perceived impact 0
2. Has the potential to cause minor alteration 3
3. Has the potential to cause moderate alteration (minor reduction in

nesting/foraging sites, reduction in habitat connectivity, interference with

native pollinators, injurious components such as spines, toxins) 7

1. Likely to cause severe alteration of higher trophic populations (extirpation or endangerment of an existing native species/population, or significant reduction

in nesting or foraging sites) 10

u. Unknown

Score:7

Documentation: Halogeton is high in oxalates and is a serious health threat to grazing

animals, especially sheep (Cronin et al. 1965). Palatability is extremely low, and halogeton is seldom eaten by livestock. It is readily grazed at times causing thousands of livestock poisoning (Whitson et al. 2000). The palatability of halogeton is listed as poor for ungulates.

in Montana, Utah, and Wyoming. In Utah and Wyoming, halogeton

palatability is fair for small mammals, good for game and nongame birds, and poor for waterfowl (Cronin et al. 1965).

Identify type of impact or alteration:

Rational:

Sources of information:

Total Possible:40

Total:20

1. Biological Characteristics and Dispersal Ability
   1. Mode of reproduction
2. Not aggressive reproduction (few [0-10] seeds per plant and no

vegetative reproduction) 0

1. Somewhat aggressive (reproduces only by seeds (11-1,000/m2) 1
2. Moderately aggressive (reproduces vegetatively and/or by a moderate

amount of seed, <1,000/m2) 2

1. Highly aggressive reproduction (extensive vegetative spread and/or

many seeded, >1,000/m2) 3

u. Unknown

Score:3

Documentation: Halogeton can produce 75 seeds per inch (35 seeds per cm) of stem, which

is 200 to 400 pounds of seeds per acre (222-449 kg/ha) Cronin et al. 1965

Describe key reproductive characteristics (including seeds per plant):

Rational:

Sources of information:

- 1. Innate potential for long-distance dispersal (bird dispersal, sticks to animal hair, buoyant fruits, wind-dispersal)

1. Does not occur (no long-distance dispersal mechanisms) 0
2. Infrequent or inefficient long-distance dispersal (occurs occasionally

despite lack of adaptations) 2

1. Numerous opportunities for long-distance dispersal (species has

adaptations such as pappus, hooked fruit-coats, etc.) 3

1. Unknown

Score:3

Documentation: Animals are capable of spreading large amounts of seed great distances; seeds pass with the feces. Wind and road works are other means

Identify dispersal mechanisms:

Rational:

Sources of information:

- 1. Potential to be spread by human activities (both directly and indirectly – possible mechanisms include: commercial sales, use as forage/revegetation, spread along highways, transport on boats, contamination, etc.)

1. Does not occur 0
2. Low (human dispersal is infrequent or inefficient) 1
3. Moderate (human dispersal occurs) 2
4. High (there are numerous opportunities for dispersal to new areas) 3

u. Unknown

Score: 3

Documentation: spread in open or disturbed ground such as dry lakebeds, railways, gravel pits, overgrazed ranges and rodent workings

Identify dispersal mechanisms:

Rational:

Sources of information:

- 1. Allelopathic

1. no 0
2. yes 2
3. unknown

Score:0

Documentation:

Describe effect on adjacent plants:

Rational:

Sources of information:

- 1. Competitive ability

a. Poor competitor for limiting factors 0

b. Moderately competitive for limiting factors 1

c. Highly competitive for limiting factors and/or nitrogen fixing ability 3

u. Unknown

Score:1

Documentation:Poor competitor because it does not establish large shoots or roots early in the growing season (Eckert 1954). It can however survive extended drought due to 10 yr dormancy of seeds (Cronin et al. 1965)

Evidence of competitive ability:

Rational:

Sources of information:

- 1. Forms dense thickets, climbing or smothering growth habit, or otherwise taller than the surrounding vegetation

1. No 0
2. Forms dense thickets 1
3. Has climbing or smothering growth habit, or otherwise taller than the surrounding

vegetation 2

u. Unknown

Score:0

Documentation:

Describe growth form:

Rational:

Sources of information:

- 1. Germination requirements

1. Requires open soil and disturbance to germinate 0
2. Can germinate in vegetated areas but in a narrow range or in special conditions 2
3. Can germinate in existing vegetation in a wide range of conditions 3

u. Unknown

Score:0

Documentation: Halogeton readily invades and dominates rangeland depleted by persistent

and continuous overgrazing (Cronin et al 1965)

Describe germination requirements:

Rational:

Sources of information:

- 1. Other species in the genus invasive in Alberta or elsewhere

1. No 0
2. Yes 3

u. Unknown

Score:0

Documentation:

Species:

Sources of information:

2.9 Aquatic, wetland, or riparian species

a. Not invasive in wetland communities 0

b. Invasive in riparian communities 1

c. Invasive in wetland communities 3

u. Unknown

Score: 0

Documentation:

Describe type of habitat:

Rational:

Sources of information:

Total Possible:25

Total:10

1. Distribution
   1. Is the species highly domesticated or a weed of agriculture
2. No 0
3. Is occasionally an agricultural pest 2
4. Has been grown deliberately, bred, or is known as a significant agricultural pest 4
5. Unknown

Score:4

Documentation: agricultural pest, low palatability to livestock as noted above

Identify reason for selection, or evidence of weedy history:

Rational:

Sources of information:

- 1. Known level of ecological impact in natural areas

1. Not known to cause impact in any other natural area 0
2. Known to cause impacts in natural areas, but in dissimilar habitats and

climate zones than exist in regions of Alberta 1

1. Known to cause low impact in natural areas in similar habitats and climate

zones to those present in Alberta 3

1. Known to cause moderate impact in natural areas in similar habitat and

climate zones 4

1. Known to cause high impact in natural areas in similar habitat and climate

zones 6

u. Unknown

Score:3

Documentation: occurs in degraded areas

Identify type of habitat and states or provinces where it occurs:

Sources of information:

- 1. Role of anthropogenic and natural disturbance in establishment

1. Requires anthropogenic disturbances to establish 0
2. May occasionally establish in undisturbed areas but can readily establish in

areas with natural disturbances 3

1. Can establish independent of any known natural or anthropogenic disturbances 5

u. Unknown

Score:0

Documentation: open or disturbed ground such as dry lakebeds, railways, gravel pits, overgrazed ranges and rodent workings

Identify type of disturbance:

Rationale:

Sources of information:

- 1. Current global distribution

1. Occurs in one or two continents or regions (e.g., Mediterranean region) 0
2. Extends over three or more continents 3
3. Extends over three or more continents, including successful introductions in

arctic or subarctic regions 5

u. Unknown

Score:3

Documentation: Occurs in North America, Australia, Asia

Describe distribution:

Rational:

Sources of information:

- 1. Extent of the species Canada range and/or occurrence of formal state or provincial listing

1. 0-5 percent of the states/provinces 0
2. 6-20 percent of the states/provinces 2
3. 21-50 percent, and/or state/province listed as a problem weed

(e.g., “Noxious,” or “Invasive”) in 1 state or Canadian province 4

1. Greater than 50 percent, and/or identified as “Noxious” in 2 or more states or

Canadian provinces 5

u. Unknown

Score:5

Documentation: Noxious in Arizona, California, Colorado, Hawaii, New Mexico and Oregon (USDA PLANTS)

Identify provinces invaded:

Rational:

Sources of information:

Total possible:25

Total:15

1. Feasibility of Control
   1. Seed banks
2. Seeds remain viable in the soil for less than 3 years 0
3. Seeds remain viable in the soil for between 3 and 5 years 2
4. Seeds remain viable in the soil for 5 years and more 3

u. Unknown

Score:3

Documentation: 2 types of seeds produced: black seds viable for 1 yr and brown seeds that can survive for 10 yrs (Cronin et al. 1965)

Identify longevity of seed bank

Rational:

Sources of information:

- 1. Vegetative regeneration

1. No resprouting following removal of aboveground growth 0
2. Resprouting from ground-level meristems 1
3. Resprouting from extensive underground system 2
4. Any plant part is a viable propagule 3

u. Unknown

Score:0

Documentation: DiTomaso et al. 2013

Describe vegetative response:

Rational:

Sources of information:

- 1. Level of effort required

1. Management is not required (e.g., species does not persist without repeated anthropogenic disturbance) 0
2. Management is relatively easy and inexpensive; requires a minor investment in human and financial resources 2
3. Management requires a major short-term investment of human and financial resources, or a moderate long-term investment 3
4. Management requires a major, long-term investment of human and financial resources 4

u. Unknown

Score:4

Documentation: The best defense against halogeton is a vigorous stand of perennial

range plants and variations in grazing patterns (Pemberton 1986)

Identify types of control methods and time-term required: chemicals, cultural, biocontrol (Cronin et al. 1965).

Rational:

Sources of information:

Total Possible: 10

Total: 7

Total for 4 sections Possible: 100

Total for 4 sections:55

References:

Cronin, Eugene H.; Williams, M. Coburn. 1965. Principles for managing

ranges infested with halogeton. Journal of Range Management. 19:

226-227.

DiTomaso, J.M., G.B. Kyser et al. 2013. Weed Control in Natural Areas in the Western United States. Weed Research and Information Center, University of California. 544 pp.

Eckert, Richard E., Jr. 1954. A study of competition between whitesage

and halogeton in Nevada. Journal of Range Management. 7: 223-225

Reynolds, Timothy D.; Trost, Charles H. 1981. Grazing, crested wheatgrass, and bird populations in southeastern Idaho. Northwest Science. 55(3): 225-234.

USDA PLANTS database

Pavek, Diane S. 1992. Halogeton glomeratus. In: Fire Effects Information System, [Online].

U.S. Department of Agriculture, Forest Service,

Rocky Mountain Research Station, Fire Sciences Laboratory (Producer).

Available: http://www.fs.fed.us/database/feis/ [2013, November 29].

Pemberton, Robert W. 1986. The distribution of halogeton in North

America. Journal of Range Management. 39(3): 281-282

Whitson, T. D., Burrill, L. C., Dewey, S. A., Cudney, D. W., Nelson, B. E., Lee, R. D., and Parker, R. 2000. Weeds of the West. Jackson, WY: Western Society of Weed Science. 628 pp.

Notes

Halogeton is an exotic succulent **annual** forb. The taproot can penetrate as deep as 20 inches. Halogeton is adapted to alkaline soils and semiarid environments. It has rapidly invaded millions of acres in the western states.

Score Interpretation

While different users will have different concepts of what constitutes various levels of invasiveness (e.g., what is “highly invasive” vs. “moderately invasive” may differ among management agencies), we divided the ranks into six blocks in Appendix A. We consider species with scores ≥80 as “Extremely Invasive” and species with scores 70–79 as “Highly Invasive;” both of these groups are composed of species estimated to be very threaten­ing to Alberta. Species with scores of 60–69 as “Moderately Invasive” and scores of 50–59 represent “Modestly Invasive” species; both of these groups still pose significant risks to ecosystems. Species with scores of 40–49 are “Weakly Invasive”, and <40 are considered “Very Weakly Invasive.” These last two groups generally have not been shown to significantly alter ecosystem processes and communities elsewhere and probably do not require as much attention as the other species.

**Alberta non-native plant invasiveness ranking form**

(Adapted from Carlson et al. 2008)

| Scientific name: | *Cytisus scoparius* |
| --- | --- |
| Common name: | Scotch Broom |
| Assessor: | Shauna-Lee Chai |
| Reviewer: | Joseph Ditomaso |
| Date: | October 11, 2013 |

Outcome score:

1. Climatic Comparison

This species is present or may potentially establish in the following natural regions:

|  | Collected in Alberta regions | CLIMEX similarity in  1975 | CLIMEX similarity in 2050 |
| --- | --- | --- | --- |
| Boreal | No | 0.738 | 0.790 |
| Parkland | No | 0.799 | 0.822 |
| Foothills | No | 0.834 | 0.859 |
| Grassland | No | 0.792 | 0.818 |
| Rocky Mountains | No | 0.773 | 0.801 |
| Shield | No | 0.650 | 0.720 |

1. Invasiveness Ranking Total (Total answered^1^ points possible) Total score

| 1. Ecological impact | 40(40) | 37 |
| --- | --- | --- |
| 1. Biological characteristic and dispersal ability | 25(25) | 17 |
| 1. Ecological amplitude and distribution | 25(25) | 16 |
| 1. Feasibility of control | 10(10) | 8 |
| Outcome score | 100(100)^b^ | ^a^78 |
| Relative maximum score^2^ | 78 | *Highly Invasive* |

^1^For questions answered “unknown” do not include point value for the question in parentheses for “Total answered points possible.”

^2^Calculated as a/b x 100.

1. Climatic Comparison:
   1. Has this species ever been collected or documented in Alberta?

__Yes – continue to 1.2

x No – continue to 2.1

Which natural region has it been collected or documented (see inset map)? Proceed to section B. Invasiveness Ranking.

__Boreal

__Rockies

__Grassland

__Foothills

__Parkland

__Shield

Documentation:

Sources of information: ANPC Rogues gallery, ACIMS, PLANTS database, GBIF

2.1 Is there a 70 percent or higher similarity (based on CLIMEX climate matching) between climates anywhere the species currently occurs and

a. Boreal - Yes

b. Rockies - Yes

c. Grassland - Yes

d. Foothills - Yes

e. Parkland - Yes

f. Shield – Not in 1975, but in 2050

-If “no” is answered for all regions, reject species from consideration

Documentation:

Sources of information:

1. Invasiveness Ranking
2. Ecological Impact
   1. Impact on Natural Ecosystem Processes
3. No perceivable impact on ecosystem processes 0
4. Has the potential to influences ecosystem processes to a minor degree

(e.g., has a perceivable but mild influence on soil nutrient availability) 3

1. Has the potential to cause significant alteration of ecosystem processes (e.g., increases sedimentation rates along streams or coastlines, reduces open water

that are important to waterfowl) 7

1. May cause major, possibly irreversible, alteration or disruption of ecosystem processes (e.g., the species alters geomorphology; hydrology; or affects fire frequency, altering community composition; species fixes substantial levels of nitrogen in the soil making soil unlikely to support certain native plants or more likely to favor non-native species) 10

u. Unknown

Score:10

Documentation: aggressive spread, creates high fire hazard, fixes nitrogen (Hoshovsky 1986)

Identify ecosystem processes impacted: fire

Rational:

Sources of information:

- 1. Impact on Natural Community Structure

1. No perceived impact; establishes in an existing layer without influencing its

structure 0

1. Has the potential to influences structure in one layer (e.g., changes the density

of one layer) 3

1. Has the potential to cause significant impact in at least one layer (e.g., creation

of a new layer or elimination of an existing layer) 7

1. Likely to cause major alteration of structure (e.g., covers canopy, eradicating

most or all layers below) 10

1. Unknown

Score:7

Documentation:Scotch broom can cover 90% of the canopy and intercept 65% of light (Prasad and Peterson 1997) in young Douglas fir plantations causing complete stand failures in some instances.

Identify type of impact or alteration:

Rational:

Sources of information:

- 1. Impact on Natural Community Composition

1. No perceived impact; causes no apparent change in native populations 0
2. Has the potential to influences community composition (e.g., reduces the

number of individuals in one or more native species in the community) 3

1. Has the potential to significantly alter community composition (e.g., produces

a significant reduction in the population size of one or more native species in

the community) 7

1. Likely to cause major alteration in community composition (e.g., results in the extirpation of one or several native species, reducing biodiversity or change the community composition towards species exotic to the natural community) 10

u. Unknown

Score:10

Documentation: renders rangeland in the USA worthless (Hoshovsky 1986), threatens endangered Garry oak ecosystems in BC., retarding establishment and spread of many rare and endemic spp in these endangered ecosystems (Nuszdorfer et al 1991). Scotch broom is reported to form monocultures and become so dense that the areas where it occurs are impenetrable (Hoshovsky 2004). In addition, this species excludes the native vegetation to such an extent that common animals such as deer and quail are unable to forage (Hoshovsky 2004).

Identify type of impact or alteration:

Rational:

Sources of information:

- 1. Impact on higher trophic levels (cumulative impact of this species on the animals,

fungi, microbes, and other organisms in the community it invades)

1. Negligible perceived impact 0
2. Has the potential to cause minor alteration 3
3. Has the potential to cause moderate alteration (minor reduction in

nesting/foraging sites, reduction in habitat connectivity, interference with

native pollinators, injurious components such as spines, toxins) 7

1. Likely to cause severe alteration of higher trophic populations (extirpation or endangerment of an existing native species/population, or significant reduction

in nesting or foraging sites) 10

u. Unknown

Score: 10

Documentation: In California, reports of livestock loss due to ingestion of toxic quinolizidine alkaloids, sparteine and isoparteine produced by Scotch broom (Parker et al 1994). Wilson and Carey (2001) found a negative relationship between Scotch broom and deer mice, suggesting that Scotch broom areas have diminished value as wildlife habitat in the Oregon while oak communities in the Puget Trough, Washington. The small areas of remaining Oregon silverspot and Fender blue butterfly habitat have been invaded by Scotch broom and other nonnative species, changing plant community composition and structure and adversely affecting the quality of habitat for the threatened butterflies (Pickering 1997).

Identify type of impact or alteration:

Rational:

Sources of information:

Total Possible:37

Total:40

1. Biological Characteristics and Dispersal Ability
   1. Mode of reproduction
2. Not aggressive reproduction (few [0-10] seeds per plant and no

vegetative reproduction) 0

1. Somewhat aggressive (reproduces only by seeds (11-1,000/m2) 1
2. Moderately aggressive (reproduces vegetatively and/or by a moderate

amount of seed, <1,000/m2) 2

1. Highly aggressive reproduction (extensive vegetative spread and/or

many seeded, >1,000/m2) 3

u. Unknown

Score:3

Documentation:

Describe key reproductive characteristics (including seeds per plant):varies from a few hundred to over 7000 pods per plant with 0-22 seeds per pod (5-8 on average) (Hosking et al. 1996). 4142 seeds/m^2^ (Smith and Harlen 1991)

Rational:

Sources of information:

- 1. Innate potential for long-distance dispersal (bird dispersal, sticks to animal hair, buoyant fruits, wind-dispersal)

1. Does not occur (no long-distance dispersal mechanisms) 0
2. Infrequent or inefficient long-distance dispersal (occurs occasionally

despite lack of adaptations) 2

1. Numerous opportunities for long-distance dispersal (species has

adaptations such as pappus, hooked fruit-coats, etc.) 3

1. Unknown

Score:2

Documentation:

Identify dispersal mechanisms: includes water, hard seed coat keep seeds viable (Williams 1981)

Rational:

Sources of information:

- 1. Potential to be spread by human activities (both directly and indirectly – possible mechanisms include: commercial sales, use as forage/revegetation, spread along highways, transport on boats, contamination, etc.)

1. Does not occur 0
2. Low (human dispersal is infrequent or inefficient) 1
3. Moderate (human dispersal occurs) 2
4. High (there are numerous opportunities for dispersal to new areas) 3

u. Unknown

Score:2

Documentation: vehicles, gravel, highway graders (Boateng 1994)

Identify dispersal mechanisms:

Rational:

Sources of information:

- 1. Allelopathic

1. no 0
2. yes 2
3. unknown

Score:0

Documentation:

Describe effect on adjacent plants:

Rational:

Sources of information:

- 1. Competitive ability

a. Poor competitor for limiting factors 0

b. Moderately competitive for limiting factors 1

c. Highly competitive for limiting factors and/or nitrogen fixing ability 3

u. Unknown

Score:3

Documentation: Efficient nitrogen fixer, drought tolerant (Wheeler et al. 1979)

Evidence of competitive ability:

Rational:

Sources of information:

- 1. Forms dense thickets, climbing or smothering growth habit, or otherwise taller than the surrounding vegetation

1. No 0
2. Forms dense thickets 1
3. Has climbing or smothering growth habit, or otherwise taller than the surrounding

vegetation 2

u. Unknown

Score:1

Documentation: forms dense thickets, monospecific stands (Erskine 1960)

Describe growth form:

Rational:

Sources of information:

- 1. Germination requirements

1. Requires open soil and disturbance to germinate 0
2. Can germinate in vegetated areas but in a narrow range or in special conditions 2
3. Can germinate in existing vegetation in a wide range of conditions 3

u. Unknown

Score:2

Documentation: germination requirements vary by environment. Seeds imbibe water before germination and germination is best after a period of dormancy at temperatures 4-33 deg. Celsius (Bossard 1993)

Describe germination requirements:

Rational:

Sources of information:

- 1. Other species in the genus invasive in Alberta or elsewhere

1. No 0
2. Yes 3

u. Unknown

Score:3

Documentation:

Species: Portuguese broom (C. striatus)-not as widespread as Scotch broom, but thought to have similar characteristics (DiTomaso 1998). It is a noxious weed in Oregon.

Sources of information: USDA PLANTS database

2.9 Aquatic, wetland, or riparian species

a. Not invasive in wetland communities 0

b. Invasive in riparian communities 1

c. Invasive in wetland communities 3

u. Unknown

Score: 1

Documentation:

Describe type of habitat:most rapid spread has occurred along water courses where the species is water dispersed (Hoshovsky, 1986)

Rational:

Sources of information:

Total Possible:25

Total:17

1. Distribution
   1. Is the species highly domesticated or a weed of agriculture
2. No 0
3. Is occasionally an agricultural pest 2
4. Has been grown deliberately, bred, or is known as a significant agricultural pest 4
5. Unknown

Score:4

Documentation:

Identify reason for selection, or evidence of weedy history: destroys rangeland. Introduced as an ornamental, used on highway banks (Peterson & Prasad, 1998). Causes economic loss in Douglas fir plantations (Prasad and Peterson 1997)

Rational:

Sources of information:

- 1. Known level of ecological impact in natural areas

1. Not known to cause impact in any other natural area 0
2. Known to cause impacts in natural areas, but in dissimilar habitats and

climate zones than exist in regions of Alberta 1

1. Known to cause low impact in natural areas in similar habitats and climate

zones to those present in Alaska 3

1. Known to cause moderate impact in natural areas in similar habitat and

climate zones 4

1. Known to cause high impact in natural areas in similar habitat and climate

zones 6

u. Unknown

Score:1

Documentation:

Identify type of habitat and states or provinces where it occurs: threatens Garry oak woodland in BC (Haber 1996) (Cowichan Garry Oak Reserve) and rare and endangered species associated with these habitats.

Sources of information:

- 1. Role of anthropogenic and natural disturbance in establishment

1. Requires anthropogenic disturbances to establish 0
2. May occasionally establish in undisturbed areas but can readily establish in

areas with natural disturbances 3

1. Can establish independent of any known natural or anthropogenic disturbances 5

u. Unknown

Score:3

Documentation: colonises disturbed sites. Does not normally grow in forests except on plantations. Can grow in river beds (Peterson and Prasad, 1998). Can establish in undisturbed areas (J. Ditomaso, pers. comm.)

Identify type of disturbance:

Rationale:

Sources of information:

- 1. Current global distribution

1. Occurs in one or two continents or regions (e.g., Mediterranean region) 0
2. Extends over three or more continents 3
3. Extends over three or more continents, including successful introductions in

arctic or subarctic regions 5

u. Unknown

Score:3

Documentation:

Describe distribution: Africa, Europe, Canada, India, Australia, New Zealand (Peterson and Prasad, 1998)

Rational:

Sources of information:

- 1. Extent of the species Canada range and/or occurrence of formal state or provincial listing

1. 0-5 percent of the states/provinces 0
2. 6-20 percent of the states/provinces 2
3. 21-50 percent, and/or state/province listed as a problem weed

(e.g., “Noxious,” or “Invasive”) in 1 state or Canadian province 4

1. Greater than 50 percent, and/or identified as “Noxious” in 2 or more states or

Canadian provinces 5

u. Unknown

Score:5

Documentation:

Identify provinces invaded:noxious weed in Hawaii, Idaho, Oregon, California, Washington

Rational:

Sources of information: USDA PLANTS database

Total possible:25

Total:16

1. Feasibility of Control
   1. Seed banks
2. Seeds remain viable in the soil for less than 3 years 0
3. Seeds remain viable in the soil for between 3 and 5 years 2
4. Seeds remain viable in the soil for 5 years and more 3

u. Unknown

Score:3

Documentation:

Identify longevity of seed bank: greater than 30 yrs (Smith and Harlen 1991)

Rational:

Sources of information:

- 1. Vegetative regeneration

1. No resprouting following removal of aboveground growth 0
2. Resprouting from ground-level meristems 1
3. Resprouting from extensive underground system 2
4. Any plant part is a viable propagule 3

u. Unknown

Score:1

Documentation: Physical and mechanical control methods applied to adult broom plants are often followed by sprouting from remaining root crowns or aboveground stems, and emergence of numerous broom seedlings (Prasad 2003)

Describe vegetative response:

Rational:

Sources of information:

- 1. Level of effort required

1. Management is not required (e.g., species does not persist without repeated anthropogenic disturbance) 0
2. Management is relatively easy and inexpensive; requires a minor investment in human and financial resources 2
3. Management requires a major short-term investment of human and financial resources, or a moderate long-term investment 3
4. Management requires a major, long-term investment of human and financial resources 4

u. Unknown

Score:4

Documentation:

Identify types of control methods and time-term required: several years are required due to seed bank and resprouting. Integrated management: Brooms are best controlled by an integrated vegetation management program including monitoring, prevention, biological control, uprooting, cutting, controlled burns, competitive planting, and spot treatments with herbicides as a last resort. The choice of specific methods, timing, and combinations depends on the site conditions and the nature of the infestation (Woo et al. 2004).

Rational:

Sources of information:

Total Possible: 10

Total: 8

Total for 4 sections Possible: 100

Total for 4 sections: 78

References:

Boateng, J. 1994. Broom: Putting it in its place. Queen’s Printer, Victoria, BC. 4 pp.

Bossard, Carla C. 1993. Seed germination in the exotic shrub Cytisus scoparius (Scotch broom) in California. Madrono. 40(1): 47-61.

Carlson, M. 2008. Invasiveness Ranking System for Non-Native Plants of Alaska. USDA. Available at: http://www.fs.usda.gov/Internet/FSE_DOCUMENTS/fsbdev2_037575.pdf

DiTomaso, Joseph M. 1998. The biology and ecology of brooms and gorse. Proceedings, California Weed Science Society. 50: 142-148.

Erskine, D. S. 1960. The plants of Prince Edward Island. Plant Research Institute, Canada Department of Agriculture,Charlottetown, PE. Publ. no. 1088, 200 pp

Haber, E. 1996. Invasive plants of Canada: 1996 National survey results. National Botanical Services, Ottawa, ON. 25 pp

Hoshovsky, M. ed. 2004. Element Stewardship Abstract for Cystisus scoparius and Genista monspessulanus. The Nature Conservancy.

Hoshovsky, M. 1986. Element Stewardship Abstract for Cytisus scoparius and Genista

monspessulanus. Scotch Broom, French Broom (http://tncweeds.ucdavis.edu). The

Nature Conservancy. Arlington, VA 19 pp.

Hosking, J. R.; Smith, J. M. B.; Sheppard, A. W. 1996. The biology of Australian weeds. 28. Cytisus scoparius (L.) Link subsp. scoparius. Plant Protection Quarterly. 11(3): 102-108.

Nuszdorfer, F. C., Klinka, K. and Demarchi, D. A. 1991. Chapter 5, pages 81–93 in D. Meidinger and J. Pojar, eds. Coastal Douglas-fir zone, in ecosystems of British Columbia. BC Min. For., Victoria, BC, Special Rep. Ser. No. 6

Parker, B., Miller, G. and Burrill, L.C. 1994. Weeds — Scotch broom, Cytisus scoparius (L.) Link. Pacific Northwest Extension Publication No. 103, Corvallis OR

Peterson, D. J. and Prasad, R. 1998. The Biology of Canadian Weeds. 109. Cytisus scoparius (L.) Link. Can. J. Plant Sci. 78: 497–504.

Pickering, Debbie L. 1997. The influence of fire on west coast grasslands and concerns about its use as a management tool: a case study of the Oregon silverspot butterfly Speyeria zerene Hippolyta (Lepidoptera, Nymphalidae). In: Greenlee, Jason M., ed. Proceedings, 1st conference on fire effects on rare and endangered species and habitats; 1995 November 13-16; Coeur d'Alene, ID. Fairfield, WA: International Association of Wildland Fire: 37-46.

Prasad, R. and Peterson, D. J. 1997. Mechanisms of Invasiveness of the exotic weed, Scotch broom (Cytisus scoparius (L.) Link) in British Columbia. Proc. Expert Committee Weeds Ann. Meeting, 9–12 December 1996, Victoria BC. pp. 197–198. (Abstr.)

Prasad, Raj. 2003. Management and control of gorse and Scotch broom in British Columbia. Technology Transfer Note Number 30. Victoria, BC: Canadian Forest Service, Natural Resources Canada, Pacific Forestry Centre, Forestry Research Applications. 6 p.

Smith, J. and Harlen, R. 1991. Preliminary observations on the seed dynamics of broom at Barrington Tops, New South Wales. Plant Protect. Q. 6: 73–78.

Wheeler, C. T., Perry, D. Helgerson, O. and Gordon, J. 1979. Winter fixation of nitrogen in Scotch broom. New Phytol. 82: 697–701.

Williams, P. A. 1981. Aspects of the ecology of broom (Cytisus scoparius) in Canterbury, New Zealand. N.Z. J. Bot. 19: 31–43.

Wilson, Suzanne M.; Carey, Andrew B. 2001. Small mammals in oak woodlands in the Puget Trough, Washington. Northwest Science. 75(4): 432-349.

Woo, Isa; Drlik, Tanya; Swiadon, Laurie; Quarles, William. 2004. Sweeping away broom--integrated management for an exotic yellow legume. The IPM Practitioner. 26(3-4): 1-8.

Zouhar, Kris. 2005. Cytisus scoparius, C. striatus. In: Fire Effects Information System, [Online]. U.S. Department of Agriculture, Forest Service, Rocky Mountain Research Station, Fire Sciences Laboratory (Producer). Available: http://www.fs.fed.us/database/feis/ [2013, October 16].

(Scotch broom invades similar habitats as gorse)

Natureserve I-rank: <http://www.natureserve.org/explorer/servlet/NatureServe?sourceTemplate=tabular_report.wmt&loadTemplate=species_RptComprehensive.wmt&selectedReport=RptComprehensive.wmt&summaryView=tabular_report.wmt&elKey=138657&paging=home&save=true&startIndex=1&nextStartIndex=1&reset=false&offPageSelectedElKey=138657&offPageSelectedElType=species&offPageYesNo=true&post_processes=&radiobutton=radiobutton&selectedIndexes=138657&selectedIndexes=142957&selectedIndexes=149850>

Scotch Broom is considered an aggressive invader and has invested more than 2 million acres in Washington, Oregon and California. It has also escaped from cultivation in the east, however, it is not as problematic there. This species, when it has established, alters abiotic characters of the ecosystem including adding nitrogen to the soil and altering the fire regime. It invades grasslands, shrublands, open forests and pastures. Finally, it produces copious seeds and requires active management.

Score interpretation

While different users will have different concepts of what constitutes various levels of invasiveness (e.g., what is “highly invasive” vs. “moderately invasive” may differ among management agencies), we divided the ranks into six blocks in Appendix A. We consider species with scores ≥80 as “Extremely Invasive” and species with scores 70–79 as “Highly Invasive;” both of these groups are composed of species estimated to be very threaten­ing to Alaska. Species with scores of 60–69 as “Moderately Invasive” and scores of 50–59 represent “Modestly Invasive” species; both of these groups still pose significant risks to ecosystems. Species with scores of 40–49 are “Weakly Invasive”, and <40 are considered “Very Weakly Invasive.” These last two groups generally have not been shown to significantly alter ecosystem processes and communities elsewhere and probably do not require as much attention as the other species.

**Alberta non-native plant invasiveness ranking form**

(Adapted from Carlson et al. 2008)

| Scientific name: | *Onopordum acanthium* |
| --- | --- |
| Common name: | Scotch thistle |
| Assessor: | Shauna-Lee Chai |
| Reviewer: | Paul Cavers |
| Date: | August 20, 2013 |

Outcome score:

1. Climatic Comparison

This species is present or may potentially establish in the following natural regions:

|  | Collected in Alberta regions | CLIMEX similarity in 1975 | CLIMEX similarity in 2050 |
| --- | --- | --- | --- |
| Boreal | No | 0.744 | 0.797 |
| Parkland | No | 0.813 | 0.856 |
| Foothills | No | 0.833 | 0.858 |
| Grassland | No | 0.852 | 0.882 |
| Rocky Mountains | No | 0.780 | 0.800 |
| Shield | No | 0.664 | 0.725 |

1. Invasiveness Ranking Total (Total answered^1^ points possible) Total score

| 1. Ecological impact | 40(40) | 20 |
| --- | --- | --- |
| 1. Biological characteristic and dispersal ability | 25(23) | 13 |
| 1. Ecological amplitude and distribution | 25(25) | 13 |
| 1. Feasibility of control | 10(10) | 7 |
| Outcome score | 100(98)^b^ | ^a^53 |
| Relative maximum score^2^ | 54 | *‘Modestly Invasive’* |

^1^For questions answered “unknown” do not include point value for the question in parentheses for “Total answered points possible.”

^2^Calculated as a/b x 100.

1. Climatic Comparison:
   1. Has this species ever been collected or documented in Alberta?

__Yes – continue to 1.2

x No – continue to 2.1

Which natural region has it been collected or documented (see inset map)? Proceed to section B. Invasiveness Ranking.

__Boreal

__Rockies

__Grassland

__Foothills

__Parkland

__Shield

Documentation:

Sources of information: ANPC Rogues gallery, ACIMS, PLANTS database, GBIF

2.1 Is there a 70 percent or higher similarity (based on CLIMEX climate matching) between climates anywhere the species currently occurs and

a. Boreal - Yes

b. Rockies - Yes

c. Grassland - Yes

d. Foothills - Yes

e. Parkland - Yes

f. Shield - Not in 1975, but in 2050

-If “no” is answered for all regions, reject species from consideration

Documentation:

Sources of information:

1. Invasiveness Ranking
2. Ecological Impact
   1. Impact on Natural Ecosystem Processes
3. No perceivable impact on ecosystem processes 0
4. Has the potential to influence ecosystem processes to a minor degree

(e.g., has a perceivable but mild influence on soil nutrient availability) 3

1. Has the potential to cause significant alteration of ecosystem processes (e.g., increases sedimentation rates along streams or coastlines, reduces open water

that are important to waterfowl) 7

1. May cause major, possibly irreversible, alteration or disruption of ecosystem processes (e.g., the species alters geomorphology; hydrology; or affects fire frequency, altering community composition; species fixes substantial levels of nitrogen in the soil making soil unlikely to support certain native plants or more likely to favor non-native species) 10

u. Unknown

Score: 7

Documentation: Dead plants remain standing and spreads fire in this manner. Affects grazing and competes with desirable forage species.

Identify ecosystem processes impacted: helps spread fire, alters community composition

Rationale:

Sources of information: ISSG, Cavers et al 2011

- 1. Impact on Natural Community Structure

1. No perceived impact; establishes in an existing layer without influencing its

structure 0

1. Has the potential to influence structure in one layer (e.g., changes the density

of one layer) 3

1. Has the potential to cause significant impact in at least one layer (e.g., creation

of a new layer or elimination of an existing layer) 7

1. Likely to cause major alteration of structure (e.g., covers canopy, eradicating

most or all layers below) 10

1. Unknown

Score: 3

Documentation: up to 3 m tall, large leaves smother other plants. Minor impact on floral strata (ISSG)

Identify type of impact or alteration:

Rationale:

Sources of information:

- 1. Impact on Natural Community Composition

1. No perceived impact; causes no apparent change in native populations 0
2. Has the potential to influence community composition (e.g., reduces the

number of individuals in one or more native species in the community) 3

1. Has the potential to significantly alters community composition (e.g., produces

a significant reduction in the population size of one or more native species in

the community) 7

1. Likely to cause major alteration in community composition (e.g., results in the extirpation of one or several native species, reducing biodiversity or change the community composition towards species exotic to the natural community) 10

u. Unknown

Score: 3

Documentation: competes with desirable forage species. One reason for the lack of persistence of Scotch thistle populations in Southern Ontario is that overwintering plants are often destroyed by populations of small rodents under the snow cover. We do not know which species of rodents are involved but in each case the growing point of the thistle plant is destroyed. This leaves a plant with large leaves in the early spring but one that cannot flower. In most thistle populations that we examined all or virtually all plants are destroyed. The occasional plants that escape destruction are usually the smallest plants and ones that occupy the least favourable sites for thistle growth and reproduction. In general, the larger and more vigorous populations of Scotch thistle are the most likely to be destroyed by this “winter grazing”- (Cavers pers. comm.)

Identify type of impact or alteration:

Rationale:

Sources of information:

- 1. Impact on higher trophic levels (cumulative impact of this species on the animals,

fungi, microbes, and other organisms in the community it invades)

1. Negligible perceived impact 0
2. Has the potential to cause minor alteration 3
3. Has the potential to cause moderate alteration (minor reduction in

nesting/foraging sites, reduction in habitat connectivity, interference with

native pollinators, injurious components such as spines, toxins) 7

1. Likely to cause severe alteration of higher trophic populations (extirpation or endangerment of an existing native species/population, or significant reduction

in nesting or foraging sites) 10

u. Unknown

Score: 7

Documentation: spines make it unpalatable, excludes cattle in California (Fuller 1977). But, can be grazed by goats, sheep in the western United States (Cavers et al. 2011)

Identify type of impact or alteration:

Rationale:

Sources of information:

Total Possible:40

Total:20

1. Biological Characteristics and Dispersal Ability
   1. Mode of reproduction
2. Not aggressive reproduction (few [0-10] seeds per plant and no

vegetative reproduction) 0

1. Somewhat aggressive (reproduces only by seeds (11-1,000/m2) 1
2. Moderately aggressive (reproduces vegetatively and/or by a moderate

amount of seed, <1,000/m2) 2

1. Highly aggressive reproduction (extensive vegetative spread and/or

many seeded, >1,000/m2) 3

u. Unknown

Score: 3

Documentation: >7500 fruits m^-2^ (1 seed/fruit) (Pettit et al. 1996)

Describe key reproductive characteristics (including seeds per plant):20±19 capitula per plant and each capitulum contains many seeds (cypselas). An average of 173 seeds per capitulum is present, which means there are 3460 seeds per plant (Steel and Threadgill, unpublished data in Cavers et al. (2011)

Rationale:

Sources of information:

- 1. Innate potential for long-distance dispersal (bird dispersal, sticks to animal hair, buoyant fruits, wind-dispersal)

1. Does not occur (no long-distance dispersal mechanisms) 0
2. Infrequent or inefficient long-distance dispersal (occurs occasionally

despite lack of adaptations) 2

1. Numerous opportunities for long-distance dispersal (species has

adaptations such as pappus, hooked fruit-coats, etc.) 3

1. Unknown

Score:3

Documentation: (Cavers et al 2011)

Identify dispersal mechanisms: dispersed by humans, wind, water, animals-livestock, wildlife, including long distance dispersal

Rationale:

Sources of information:

- 1. Potential to be spread by human activities (both directly and indirectly – possible mechanisms include: commercial sales, use as forage/revegetation, spread along highways, transport on boats, contamination, etc.)

1. Does not occur 0
2. Low (human dispersal is infrequent or inefficient) 1
3. Moderate (human dispersal occurs) 2
4. High (there are numerous opportunities for dispersal to new areas) 3

u. Unknown

Score: 2

Documentation:

Identify dispersal mechanisms:

Rationale:

Sources of information: ISSG, Cavers et al. (2011)

- 1. Allelopathic

1. no 0
2. yes 2
3. unknown

Score:unknown

Documentation: anecdotal evidence exists that the distribution of Scotch thistle plants in a number of communities may be suggestive of allelopathic effects from these plants but this is unconfirmed (Cavers pers. comm.). No evidence of allelopathy given in Cavers et al. (2011).

Describe effect on adjacent plants:

Rationale:

Sources of information:

- 1. Competitive ability

a. Poor competitor for limiting factors 0

b. Moderately competitive for limiting factors 1

c. Highly competitive for limiting factors and/or nitrogen fixing ability 3

u. Unknown

Score:1

Documentation:

Evidence of competitive ability: competes with desirable forages

Rationale:

Sources of information: Cavers et al 2011

- 1. Forms dense thickets, climbing or smothering growth habit, or otherwise taller than the surrounding vegetation

1. No 0
2. Forms dense thickets 1
3. Has climbing or smothering growth habit, or otherwise taller than the surrounding

vegetation 2

u. Unknown

Score:0

Documentation: Does not form dense thickets in Canada (Cavers pers. comm.)

Describe growth form: up to 3 m tall, broad leaves

Rationale:

Sources of information: Cavers et al 2011

- 1. Germination requirements

1. Requires open soil and disturbance to germinate 0
2. Can germinate in vegetated areas but in a narrow range or in special conditions 2
3. Can germinate in existing vegetation in a wide range of conditions 3

u. Unknown

Score:0

Documentation:

Describe germination requirements: open areas, strong dormancy of Scotch thistle seeds 5-15 years in Ontario (Cavers pers. comm.)

Rationale:

Sources of information: Cavers et al 2011

- 1. Other species in the genus invasive in Alberta or elsewhere

1. No 0
2. Yes 3

u. Unknown

Score:3

Documentation: Onopordum illyricum L. is very similar to Scotch thistle and it has been invasive in Australia for many years (Auld and Medd 1987; Parsons and Cuthbertson 1992). Small populations of this species have become established in California and it has been grown in gardens in several places in Canada (Cavers et al. 2011). Onopordum acaulon L. (stemless thistle) is a widely distributed alien weed in Australia. O. tauricum Willd., native to southeastern Europe and southwest Asia is invasive in California and Colorado and is a noxious weed in both states

Species:

Sources of information: Cavers et al 2011

2.9 Aquatic, wetland, or riparian species

a. Not invasive in wetland communities 0

b. Invasive in riparian communities 1

c. Invasive in wetland communities 3

u. Unknown

Score: 1

Documentation: There is general agreement in North America that Scotch thistle does not grow in wetlands. However, it does occur in riparian communities. It has been recorded from gravel bars in and beside the Thames River; sites that become very dry during summer droughts (Cavers pers. comm.). Elsewhere it is present in rivers canals waterways (ISSG)

Describe type of habitat:

Rationale:

Sources of information:

Total Possible:23

Total:13

1. Distribution
   1. Is the species highly domesticated or a weed of agriculture
2. No 0
3. Is occasionally an agricultural pest 2
4. Has been grown deliberately, bred, or is known as a significant agricultural pest 4
5. Unknown

Score: 4

Documentation: weed in southern Ontario (Moore and Frankton 1974)

Identify reason for selection, or evidence of weedy history:

Rationale:

Sources of information:

- 1. Known level of ecological impact in natural areas

1. Not known to cause impact in any other natural area 0
2. Known to cause impacts in natural areas, but in dissimilar habitats and

climate zones than exist in regions of Alberta 1

1. Known to cause low impact in natural areas in similar habitats and climate

zones to those present in Alberta 3

1. Known to cause moderate impact in natural areas in similar habitat and

climate zones 4

1. Known to cause high impact in natural areas in similar habitat and climate

zones 6

u. Unknown

Score:1

Documentation: minor invader of protected natural areas in Southern Ontario. Likely source is from gravel used for riverside stabilization (Cavers pers. comm.)

Identify type of habitat and states or provinces where it occurs:

Sources of information:

- 1. Role of anthropogenic and natural disturbance in establishment

1. Requires anthropogenic disturbances to establish 0
2. May occasionally establish in undisturbed areas but can readily establish in

areas with natural disturbances 3

1. Can establish independent of any known natural or anthropogenic disturbances 5

u. Unknown

Score:0

Documentation: in waste places and roadsides

Identify type of disturbance:

Rationale:

Sources of information: Cavers et al 2011

- 1. Current global distribution

1. Occurs in one or two continents or regions (e.g., Mediterranean region) 0
2. Extends over three or more continents 3
3. Extends over three or more continents, including successful introductions in

arctic or subarctic regions 5

u. Unknown

Score:3

Documentation: in all continents except Antarctica

Describe distribution: Native to southern Europe and southwestern Asia, it has been introduced to almost 50 countries in all continents except Antarctica. Does not appear to tolerate subarctic or arctic conditions.

Rationale:

Sources of information: cavers et al 2011

- 1. Extent of the species Canada range and/or occurrence of formal state or provincial listing

1. 0-5 percent of the states/provinces 0
2. 6-20 percent of the states/provinces 2
3. 21-50 percent, and/or state/province listed as a problem weed

(e.g., “Noxious,” or “Invasive”) in 1 state or Canadian province 4

1. Greater than 50 percent, and/or identified as “Noxious” in 2 or more states or

Canadian provinces 5

u. Unknown

Score:5

Documentation: regulated in 6 nearby states/provinces. Regulated in Arizona, California, Colorado, Connecticut, Idaho, Missouri, Nevada, new Mexico, Oklahoma, Oregon, Utah, Washington, Wyoming

Identify provinces invaded:BC, Ont, Quebec, Montana, Wyoming

Rationale:

Sources of information:

Total possible:25

Total:13

1. Feasibility of Control
   1. Seed banks
2. Seeds remain viable in the soil for less than 3 years 0
3. Seeds remain viable in the soil for between 3 and 5 years 2
4. Seeds remain viable in the soil for 5 years and more 3

u. Unknown

Score:3

Documentation:>15% germination after 39 years, persistent seed bank in S-W Ontario

Identify longevity of seed bank

Rationale:

Sources of information: Cavers et al. (2011)

- 1. Vegetative regeneration

1. No resprouting following removal of aboveground growth 0
2. Resprouting from ground-level meristems 1
3. Resprouting from extensive underground system 2
4. Any plant part is a viable propagule 3

u. Unknown

Score:1

Documentation: resprouting after cultivation which splits large rosettes (Cavers et al 2011). Parsons and Cuthbertson (1992): “Isolated plants should be grubbed, taking care to remove as much of the taproot as possible, otherwise regrowth occurs from any substantial pieces of the root left intact.” In contrast, Cavers found that regrowth only occurs from fragments that include growing points from the vegetative stem tissue and has not recorded more than 3 individuals after fragmentation of a single non-flowering plant. Possibly, there is more vegetative regeneration from fragmented plants of the hybrid (in Australia) than from plants of Onopordum acanthium in North America. Hybrids from other weed species do have a greater capacity for vegetative regeneration than plants from a single species (e.g. Rumex, Cavers, unpublished).

Describe vegetative response:

Rationale:

Sources of information:

- 1. Level of effort required

1. Management is not required (e.g., species does not persist without repeated anthropogenic disturbance) 0
2. Management is relatively easy and inexpensive; requires a minor investment in human and financial resources 2
3. Management requires a major short-term investment of human and financial resources, or a moderate long-term investment 3
4. Management requires a major, long-term investment of human and financial resources 4

u. Unknown

Score:3

Documentation:

Identify types of control methods and time-term required: digging, mowing, herbicide. Multiyear control efforts have been recorded in Australia, initiated in 1968, and by 1987 the area infested was reduced from 1449 ha to250 ha.

Rationale:

Sources of information: Briese et al. 1990

Total Possible: 10

Total: 7

Total for 4 sections Possible: 98

Total for 4 sections: 53

Score Interpretation (Carlson et al. 2008):

While different users will have different concepts of what constitutes various levels of invasiveness (e.g., what is “highly invasive” vs. “moderately invasive” may differ among management agencies), we divided the ranks into six blocks in Appendix A. We consider species with scores ≥80 as “Extremely Invasive” and species with scores 70–79 as “Highly Invasive;” both of these groups are composed of species estimated to be very threatening to Alberta. Species with scores of 60–69 as “Moderately Invasive” and scores of 50–59 represent “Modestly Invasive” species; both of these groups still pose significant risks to ecosystems. Species with scores of 40–49 are “Weakly Invasive”, and <40 are considered “Very Weakly Invasive.” These last two groups generally have not been shown to significantly alter ecosystem processes and communities elsewhere and probably do not require as much attention as the other species

Notes:

Onopordum acanthium could be a hybrid between Onopordum acanthium and Onopordum illyricum L. (Illyrian thistle) (Cavers pers. comm.).

References:

Briese, D. T., Lane, D., Hyde-Wyatt, B. H., Crocker, J. and Diver, R. G. 1990. Distribution of thistles of the genus Onopordum in Australia. Plant Prot. Q. 5:23-27.

Carlson, M. 2008. Invasiveness Ranking System for Non-Native Plants of Alaska. USDA. Available at: http://www.fs.usda.gov/Internet/FSE_DOCUMENTS/fsbdev2_037575.pdf

Cavers, P.B. M. Qaderi, P. Threadgill & M. Steel. 2011. The Biology of Canadian Weeds. 147. *Onopordum acanthium* L. Can. J. Plant Sci. 91, 739-758.

Fuller, T. C. 1958. Scotch thistle (Onopordum acanthium) in California. Calif. State Dep. Agric. Bull. 47: 222-223

ISSG database <http://www.issg.org/database/species/ecology.asp?si=295&fr=1&sts=&lang=EN>. Accessed August 20, 2013.

Pettit, W. J., Briese, D. T., Walker, A., Woodburn, T. L. and Corey, S. 1996. Aspects of thistle population dynamics with reference to Onopordum. Plant Prot. Q. 11 (Suppl. 2): 232-235.

**Alberta non-native plant invasiveness ranking form**

(Adapted from Carlson et al. 2008)

| Scientific name: | *Zygophyllum fabago* |
| --- | --- |
| Common name: | Syrian Bean-caper |
| Assessor: | Shauna-Lee Chai |
| Reviewer: | John Kartesz |
| Date: | October 23, 2013 |

Outcome score:

1. Climatic Comparison

This species is present or may potentially establish in the following natural regions:

|  | Collected in Alberta regions | CLIMEX similarity in 1975 | CLIMEX similarity in 2050 |
| --- | --- | --- | --- |
| Boreal | No | 0.742 | 0.753 |
| Parkland | No | 0.762 | 0.804 |
| Foothills | No | 0.722 | 0.758 |
| Grassland | No | 0.829 | 0.861 |
| Rocky Mountains | No | 0.625 | 0.650 |
| Shield | No | 0.701 | 0.731 |

1. Invasiveness Ranking Total (Total answered^1^ points possible) Total score

| 1. Ecological impact | 40(40) | 24 |
| --- | --- | --- |
| 1. Biological characteristic and dispersal ability | 25(16) | 6 |
| 1. Ecological amplitude and distribution | 25(19) | 10 |
| 1. Feasibility of control | 10(7) | 6 |
| Outcome score | 100(82)^b^ | ^a^46 |
| Relative maximum score^2^ | 56 | *Modestly Invasive* |

^1^For questions answered “unknown” do not include point value for the question in parentheses for “Total answered points possible.”

^2^Calculated as a/b x 100.

1. Climatic Comparison:
   1. Has this species ever been collected or documented in Alberta?

__Yes – continue to 1.2

x No – continue to 2.1

1.2 Which natural region has it been collected or documented? Proceed to section B. Invasiveness Ranking.

__Boreal

__Rockies

__Grassland

__Foothills

__Parkland

__Shield

Documentation:

Sources of information: ANPC Rogues gallery, ACIMS, PLANTS database, GBIF

2.1 Is there a 70 percent or higher similarity (based on CLIMEX climate matching) between climates anywhere the species currently occurs and

a. Boreal - Yes

b. Rockies - No

c. Grassland - Yes

d. Foothills - Yes

e. Parkland - Yes

f. Shield - Yes

-If “no” is answered for all regions, reject species from consideration

Documentation:

Sources of information:

1. Invasiveness Ranking
2. Ecological Impact
   1. Impact on Natural Ecosystem Processes
3. No perceivable impact on ecosystem processes 0
4. Has the potential to influence ecosystem processes to a minor degree

(e.g., has a perceivable but mild influence on soil nutrient availability) 3

1. Has the potential to cause significant alteration of ecosystem processes (e.g., increases sedimentation rates along streams or coastlines, reduces open water

that are important to waterfowl) 7

1. May cause major, possibly irreversible, alteration or disruption of ecosystem processes (e.g., the species alters geomorphology; hydrology; or affects fire frequency, altering community composition; species fixes substantial levels of nitrogen in the soil making soil unlikely to support certain native plants or more likely to favor non-native species) 10

u. Unknown

Score: 3

Documentation: excludes native species and competes for water (Davison & Wargo 2001)

Identify ecosystem processes impacted:

Rationale:

Sources of information:

- 1. Impact on Natural Community Structure

1. No perceived impact; establishes in an existing layer without influencing its

structure 0

1. Has the potential to influence structure in one layer (e.g., changes the density

of one layer) 3

1. Has the potential to cause significant impact in at least one layer (e.g., creation

of a new layer or elimination of an existing layer) 7

1. Likely to cause major alteration of structure (e.g., covers canopy, eradicating

most or all layers below) 10

1. Unknown

Score: 7

Documentation: Grows to over 3 feet tall and displaces native species (Davison & Wargo 2001).

Identify type of impact or alteration:

Rationale:

Sources of information:

- 1. Impact on Natural Community Composition

1. No perceived impact; causes no apparent change in native populations 0
2. Has the potential to influence community composition (e.g., reduces the

number of individuals in one or more native species in the community) 3

1. Has the potential to significantly alter community composition (e.g., produces

a significant reduction in the population size of one or more native species in

the community) 7

1. Likely to cause major alteration in community composition (e.g., results in the extirpation of one or several native species, reducing biodiversity or change the community composition towards species exotic to the natural community) 10

u. Unknown

Score: 7

Documentation: Forms dense colonies that excludes other species. Syrian bean caper is related to both Puncture vine and African rue. (Davidson and Wargo 2001)

Identify type of impact or alteration:

Rationale:

Sources of information:

- 1. Impact on higher trophic levels (cumulative impact of this species on the animals,

fungi, microbes, and other organisms in the community it invades)

1. Negligible perceived impact 0
2. Has the potential to cause minor alteration 3
3. Has the potential to cause moderate alteration (minor reduction in

nesting/foraging sites, reduction in habitat connectivity, interference with

native pollinators, injurious components such as spines, toxins) 7

1. Likely to cause severe alteration of higher trophic populations (extirpation or endangerment of an existing native species/population, or significant reduction

in nesting or foraging sites) 10

u. Unknown

Score: 7

Documentation: not palatable to wildlife and cattle, decreases available forage (Davison & Wargo 2001)

Identify type of impact or alteration:

Rationale:

Sources of information:

Total Possible:40

Total:24

1. Biological Characteristics and Dispersal Ability
   1. Mode of reproduction
2. Not aggressive reproduction (few [0-10] seeds per plant and no

vegetative reproduction) 0

1. Somewhat aggressive (reproduces only by seeds (11-1,000/m2) 1
2. Moderately aggressive (reproduces vegetatively and/or by a moderate

amount of seed, <1,000/m2) 2

1. Highly aggressive reproduction (extensive vegetative spread and/or

many seeded, >1,000/m2) 3

u. Unknown

Score:3

Documentation: reproduces by rhizomes and seed (Davison & Wargo 2001)

Describe key reproductive characteristics (including seeds per plant):

Rationale:

Sources of information:

- 1. Innate potential for long-distance dispersal (bird dispersal, sticks to animal hair, buoyant fruits, wind-dispersal)

1. Does not occur (no long-distance dispersal mechanisms) 0
2. Infrequent or inefficient long-distance dispersal (occurs occasionally

despite lack of adaptations) 2

1. Numerous opportunities for long-distance dispersal (species has

adaptations such as pappus, hooked fruit-coats, etc.) 3

1. Unknown

Score:unknown

Documentation:

Identify dispersal mechanisms:

Rationale:

Sources of information:

- 1. Potential to be spread by human activities (both directly and indirectly – possible mechanisms include: commercial sales, use as forage/revegetation, spread along highways, transport on boats, contamination, etc.)

1. Does not occur 0
2. Low (human dispersal is infrequent or inefficient) 1
3. Moderate (human dispersal occurs) 2
4. High (there are numerous opportunities for dispersal to new areas) 3

u. Unknown

Score: unknown

Documentation:

Identify dispersal mechanisms:

Rationale:

Sources of information:

- 1. Allelopathic

1. no 0
2. yes 2
3. unknown

Score:0

Documentation:

Describe effect on adjacent plants:

Rationale:

Sources of information:

- 1. Competitive ability

a. Poor competitor for limiting factors 0

b. Moderately competitive for limiting factors 1

c. Highly competitive for limiting factors and/or nitrogen fixing ability 3

u. Unknown

Score:3

Documentation: drought tolerant-thick waxy leaves, extensive root system allows greater water uptake than in other species (Davison and Wargo 2001)

Evidence of competitive ability:

Rationale:

Sources of information:

- 1. Forms dense thickets, climbing or smothering growth habit, or otherwise taller than the surrounding vegetation

1. No 0
2. Forms dense thickets 1
3. Has climbing or smothering growth habit, or otherwise taller than the surrounding

vegetation 2

u. Unknown

Score:0

Documentation: Does not form thickets (Kartesz pers. comm.). Reduces native biodiversity by forming dense patches that compete with native plants for water and space. Thrives in dry desert alkaline soil (GoC 2013)

Describe growth form:

Rationale:

Sources of information:

- 1. Germination requirements

1. Requires open soil and disturbance to germinate 0
2. Can germinate in vegetated areas but in a narrow range or in special conditions 2
3. Can germinate in existing vegetation in a wide range of conditions 3

u. Unknown

Score:unknown

Documentation:

Describe germination requirements:

Rationale:

Sources of information:

- 1. Other species in the genus invasive in Alberta or elsewhere

1. No 0
2. Yes 3

u. Unknown

Score:0

Documentation:

Species:

Sources of information:

2.9 Aquatic, wetland, or riparian species

a. Not invasive in wetland communities 0

b. Invasive in riparian communities 1

c. Invasive in wetland communities 3

u. Unknown

Score:0

Documentation:

Describe type of habitat: dry or alkaline soil (Davison & Wargo 2001)

Rationale:

Sources of information:

Total Possible:16

Total:6

1. Distribution
   1. Is the species highly domesticated or a weed of agriculture
2. No 0
3. Is occasionally an agricultural pest 2
4. Has been grown deliberately, bred, or is known as a significant agricultural pest 4
5. Unknown

Score:2

Documentation: Does not appear to affect crops, but affects livestock.

Identify reason for selection, or evidence of weedy history:

Rationale:

Sources of information:

- 1. Known level of ecological impact in natural areas

1. Not known to cause impact in any other natural area 0
2. Known to cause impacts in natural areas, but in dissimilar habitats and

climate zones than exist in regions of Alberta 1

1. Known to cause low impact in natural areas in similar habitats and climate

zones to those present in Alberta 3

1. Known to cause moderate impact in natural areas in similar habitat and

climate zones 4

1. Known to cause high impact in natural areas in similar habitat and climate

zones 6

u. Unknown

Score:unknown

Documentation:

Identify type of habitat and states or provinces where it occurs:

Sources of information:

- 1. Role of anthropogenic and natural disturbance in establishment

1. Requires anthropogenic disturbances to establish 0
2. May occasionally establish in undisturbed areas but can readily establish in

areas with natural disturbances 3

1. Can establish independent of any known natural or anthropogenic disturbances 5

u. Unknown

Score:0

Documentation: Grows well in areas with disturbance-roadsides, pastures, gravel pits, overgrazed areas (Davison and Wargo 2001)

Identify type of disturbance:

Rationale:

Sources of information:

- 1. Current global distribution

1. Occurs in one or two continents or regions (e.g., Mediterranean region) 0
2. Extends over three or more continents 3
3. Extends over three or more continents, including successful introductions in

arctic or subarctic regions 5

u. Unknown

Score:3

Documentation: Has not been recorded in Canada (USDA PLANTS database)

Describe distribution: Native to Mediterranean and central Asia. Introduced to Australia and southern Europe and the USA (GoC 2013).

Rationale:

Sources of information:

- 1. Extent of the species Canada range and/or occurrence of formal state or provincial listing

1. 0-5 percent of the states/provinces 0
2. 6-20 percent of the states/provinces 2
3. 21-50 percent, and/or state/province listed as a problem weed

(e.g., “Noxious,” or “Invasive”) in 1 state or Canadian province 4

1. Greater than 50 percent, and/or identified as “Noxious” in 2 or more states or

Canadian provinces 5

u. Unknown

Score:5

Documentation: Noxious in California, Idaho, Washington, Oregon (USDA PLANTS database)

Identify provinces invaded: Not recorded in Canada

Rationale:

Sources of information:

Total possible:19

Total:10

1. Feasibility of Control
   1. Seed banks
2. Seeds remain viable in the soil for less than 3 years 0
3. Seeds remain viable in the soil for between 3 and 5 years 2
4. Seeds remain viable in the soil for 5 years and more 3

u. Unknown

Score:unknown

Documentation:

Identify longevity of seed bank

Rationale:

Sources of information:

- 1. Vegetative regeneration

1. No resprouting following removal of aboveground growth 0
2. Resprouting from ground-level meristems 1
3. Resprouting from extensive underground system 2
4. Any plant part is a viable propagule 3

u. Unknown

Score:2

Documentation: can generate from root fragments (GoC 2013)

Describe vegetative response:

Rationale:

Sources of information:

- 1. Level of effort required

1. Management is not required (e.g., species does not persist without repeated anthropogenic disturbance) 0
2. Management is relatively easy and inexpensive; requires a minor investment in human and financial resources 2
3. Management requires a major short-term investment of human and financial resources, or a moderate long-term investment 3
4. Management requires a major, long-term investment of human and financial resources 4

u. Unknown

Score:4

Documentation: difficult to control with herbicide and mechanical means creates root fragments that resprout (GoC 2013)

Identify types of control methods and time-term required:

Rationale:

Sources of information:

Total Possible:7

Total:6

Total for 4 sections Possible: 82

Total for 4 sections: 46

References:

Carlson, M. 2008. Invasiveness Ranking System for Non-Native Plants of Alaska. USDA. Available at: http://www.fs.usda.gov/Internet/FSE_DOCUMENTS/fsbdev2_037575.pdf

GoC. 2013. *Zygophyllum fabago* L (Syrian Bean-caper) - Fact Sheet [http://www.inspection.gc.ca/plants/plant-protection/invasive-plants/fact-sheets/zygophyllum-fabago/eng/1331820817035/1331820887749](GoC%202013.%20http://www.inspection.gc.ca/plants/plant-protection/invasive-plants/fact-sheets/zygophyllum-fabago/eng/1331820817035/1331820887749)

Davison, J., and Wargo, M. (2001) Syrian Beancaper: Another New Noxious Weed Threatens Nevada. University of Nevada Cooperative Extenson factsheet: FS-01-46.

Notes:

inhabits dry habitats

While different users will have different concepts of what constitutes various levels of invasiveness (e.g., what is “highly invasive” vs. “moderately invasive” may differ among management agencies), we divided the ranks into six blocks in Appendix A. We consider species with scores ≥80 as “Extremely Invasive” and species with scores 70–79 as “Highly Invasive;” both of these groups are composed of species estimated to be very threaten­ing to Alberta. Species with scores of 60–69 as “Moderately Invasive” and scores of 50–59 represent “Modestly Invasive” species; both of these groups still pose significant risks to ecosystems. Species with scores of 40–49 are “Weakly Invasive”, and <40 are considered “Very Weakly Invasive.” These last two groups generally have not been shown to significantly alter ecosystem processes and communities elsewhere and probably do not require as much attention as the other species.
